# Comparative analysis of the intestinal microbiome in *Rattus norvegicus* from different geographies

**DOI:** 10.3389/fmicb.2023.1283453

**Published:** 2023-11-03

**Authors:** Taif Shah, Yutong Hou, Jinyong Jiang, Zahir Shah, Yuhan Wang, Qian Li, Xiang Xu, Yixuan Wang, Binghui Wang, Xueshan Xia

**Affiliations:** ^1^Faculty of Life Science and Technology, Kunming University of Science and Technology, Kunming, Yunnan, China; ^2^Yunnan International Joint Laboratory of Vector Biology and Control & Yunnan Provincial Key Laboratory of Vector-Borne Diseases Control and Research of Yunnan Institute of Parasitic Diseases, Yunnan, China; ^3^College of Veterinary Sciences, The University of Agriculture, Peshawar, Pakistan; ^4^Research Institute of Forest Protection, Yunnan Academy of Forestry and Grassland, Kunming, Yunnan, China; ^5^School of Public Health, Kunming Medical University, Kunming, Yunnan, China

**Keywords:** *Rattus norvegicus*, Yunnan, intestines, microbiome, pathogens

## Abstract

Rat species *Rattus norvegicus*, also known as the brown street rat, is the most abundant mammal after humans in urban areas, where they co-exist with humans and domestic animals. The reservoir role of *R. norvegicus* of zoonotic pathogens in cities among rodent-borne diseases that could endanger the lives of humans and other mammals. Therefore, understanding the normal microbiome of *R. norvegicus* is crucial for understanding and preventing zoonotic pathogen transmission to humans and animals. We investigated the intestinal microbiome of free-living *R. norvegicus* collected from the Ruili, Nujiang, and Lianhe regions of Yunnan, China, using 16S rRNA gene sequence analysis. Proteobacteria, followed by Firmicutes, and Bacteroidetes were abundant in the intestines of *R. norvegicus*; however, bacterial compositions varied significantly between samples from different locations. Following a similar trend, Gammaproteobacteria, Bacilli, and Clostridia were among the top bacterial classes in most intestinal samples. The situation differed slightly for the Lianhe and Nujiang samples, although Phyla Bacteroidota and Spirochaetota were most prevalent. The Alpha diversity, Chao1, and Simpson indexes revealed microbial richness among the *R. norvegicus* samples. A slight variation was observed among the samples collected from Ruili, Nujiang, and Lianhe. At species levels, several opportunistic and zoonotic bacterial pathogens, including *Lactococcus garvieae*, *Uruburuella suis*, *Bartonella australis*, *Clostridium perfringens*, *Streptococcus azizii*, *Vibrio vulnificus*, etc., were revealed in the *R. norvegicus* intestines, implying the need for a regular survey to monitor and control rodent populations. In conclusion, we explored diverse microbial communities in *R. norvegicus* intestines captured from different regions. Further, we identified several opportunistic and potential bacterial pathogens, which still need to be tested for their underlying pathogenesis. The findings of our current study should be considered a warning to the health authorities to implement rat control and surveillance strategies globally.

## Introduction

Microbial communities, including archaea, bacteria, and fungi, coexist with the mammalian digestive tract, regulating host homeostasis, physiology, and immune function (Le Roy et al., 2019). Host-microbiome relationships are maintained throughout the host’s lifetime and are thus important for our understanding of life. Hosts and microbiome associations can benefit from the association in various ways (Brahe et al., 2016). Perturbations to these relationships in humans and animals have resulted in changes in the microbial structures (Pickard et al., 2017) that further influence host functions, leading to dysbiosis and increased susceptibility to disorders, including obesity, inflammatory bowel disease, autoimmune disorders, etc. (Brahe et al., 2016; Larabi et al., 2020). This raises several concerns for wild animal conservation initiatives regarding how changes in the natural environment may influence intestinal microbiome community compositions. Little research has been conducted on the microbiome communities along the gastrointestinal tract of wild animals, particularly rodents (Anders et al., 2021). So far, no study has looked into the intestinal microbial community compositions of free-living *R. norvegicus* from the Ruili, Nujiang, and Lianhe regions of Yunnan Province, China.

Rodent models have become crucial in studying the gastrointestinal tract microbiome compositions, particularly in the intestines. Due to limitations in human research, the rat was the first mammalian species domesticated for scientific purposes. *R. norvegicus* is a social animal that is large enough to be easily grasped and is regarded as one of the best rodent pets due to its calm nature. So far, scientists have frequently used different rat models, including diseased or genetically altered models, to understand host–microbe interactions (Fritz et al., 2013). Obesity, chronic respiratory disease, and tumors are common in older *R. norvegicus*, but their biological system is similar to that of mice, making them ideal for microbiome research (Franklin and Ericsson, 2017). In addition, rats’ physiological parameters are closely related to those of humans (Franklin and Ericsson, 2017). In contrast, mice have different living environments and immune systems from those observed in humans (Fritz et al., 2013).

Microbiome community compositions vary from individual to individual. The phyla Firmicutes and Bacteroidetes dominate the gut region of captive and wild rodents, accounting for approximately >80% of their abundance (Bensch et al., 2023). In contrast, the human microbiota mainly comprises Actinobacteria, Proteobacteria, Verrucomicrobia, Fusobacteria, Tenericutes, Spirochetes, etc. (Hillman et al., 2017). A comparative microbiome analysis in the guts of humans, mice, rats, and non-human primates revealed that the beta diversity of humans and non-human primates showed high similarity (Nagpal et al., 2018). In contrast, Prevotella and Clostridiales were abundant in rats, while Clostridiales and Oscillospira were abundant in mice. Furthermore, humans had more Bacteroides, Ruminococcaceae, and Clostridiales than rats and mice (Nagpal et al., 2018). A similar study reports obvious differences in the intestinal microbiome of two different Kunming mouse models, HFA-KM and HFA-C57BL/6 J (Zhang et al., 2014). Genus *Clostridium* dominated HFA-KM, whereas *Blautia* dominated HFA-C57BL/6J, implying that genotype differences may shape rodent intestinal microflora diversity. Another study revealed abundant Bacteroidetes, Firmicutes, and Proteobacteria along the digestive tract of healthy rats, whereas *Lactobacillus* and *Alistipes* primarily dominated the gut regions. It has also been shown that the upper digestive tract segments had more bacterial diversity than the lower. At the same time, large intestines had diverse and richer microbial profiles; however, microbial diversity in the gastric and duodenum is comparable (Li et al., 2017).

According to the available literature, wild rats are reservoirs for various pathogens that can severely threaten public health due to their close interactions with humans and domestic animals (Easterbrook et al., 2007; Shah et al., 2023). *R. norvegicus* is prevalent in urban environments and threatens public health through its destructive behavior. In a very recent study, we reported several opportunistic and pathogenic bacteria, including *Bordetella*, *Clostridium*, *Corynebacterium*, *Empedobacter*, *Helicobacter*, *Limosilactobacillus*, *Macrococcus*, *Neisseria zaloph*, *Porphyromonas*, *Pseudomonas*, *Rahnella*, *Ralstonia*, *Rhodococcus*, *Rickettsiella*, *Streptococcus*, etc. in the free-living urban *R. norvegicus* (Shah et al., 2023). *R. norvegicus* is also reported to be a host for a large number of blood-sucking ectoparasites, such as Polyplax lice, fleas, fur mites, mesostigmatid mites, etc., in the West Indies (Thille et al., 2019). Another study investigated free-living *R. norvegicus* for the presence of zoonotic viruses, parasites, and bacteria in Maryland, United States (Easterbrook et al., 2007). They detected antibodies in *R. norvegicus* against the *hepatitis E virus*, the *Seoul virus*, *Bartonella elizabethae*, *Leptospira interrogans*, and *Rickettsia typhi*. Due to the presence of many zoonotic pathogens, *R. norvegicus* may seriously threaten the lives of humans and other mammals.

Laboratory rats have been helpful animal models for studying intestinal microbiota for the last decade. However, more researchers have focused on studying microbial community profiles in the gut samples of laboratory rats (Fritz et al., 2013; Nagpal et al., 2018) and fewer on wild rats (Gurbanov et al., 2022). Therefore, we designed this study to investigate microbial community compositions in the intestines of this important rodent. We report diverse microbial profiles in the intestines of *R. norvegicus* collected from three different regions of Yunnan Province. We also revealed several opportunistic and zoonotic pathogens, highlighting the medical importance of these free-living rodents for public health.

## Materials and methods

### Study site selection and sampling

We captured 10 free-living adult rat species (*R. norvegicus*) from three different municipalities (i.e., two from Ruili, four from Nujiang, and four from Lianhe) in Yunnan Province using mousetrap cages between January 2023 and July 2023. The animal trap locations included dense vegetation near residential areas and farmland. All the trapped *R. norvegicus* were anesthetized with cervical dislocation, after which they were dissected. A total of 10 intestinal samples (one from each animal) were collected aseptically and stored at −80°C until further processing.

The experiment was conducted according to the guidelines for using laboratory animals, Faculty of Life Science and Technology, Kunming University of Science and Technology, Kunming, Yunnan Province of China (protocol no. 16048).

### Microbial DNA extraction

Genomic DNA was extracted from each intestinal tissue sample using the TIANamp Bacterial DNA Mini Kit (TIANGEN Bio., Co., Ltd., Beijing, China) following company protocol. Briefly, the tissue samples in the Eppendorf tubes were centrifuged, and the supernatant was discarded. The pellet was dissolved in GA buffer (200 μL), Proteinase K enzyme (20 μL), and 95% ethanol (220 μL), followed by brief centrifugation. After centrifugation, the solution was transferred to the column tube and then centrifuged. The effluent was discarded, and PW buffer (600 μL) was added to the column tube for centrifugation. The column tube was placed back onto a 1.5 mL Eppendorf tube before adding TE buffer (100 μL). Centrifugation was performed after holding the tube for two mins at room temperature, and DNA-containing effluent was collected. The extracted DNA integrity and purity were determined using agarose gel and a NanoDrop Spectrophotometer (Thermo Fisher, United States).

### Bacterial 16S rDNA gene sequencing and analysis

The Illumina 16S rDNA gene amplicon was used to prepare the sequencing samples. The variable V3-V4 region of the 16S rDNA gene was amplified for microbial profiling with PCR-specific primers: 338F (5′ACTCCTACGGGAGGCAGCA3′) and 806R (5′GGACTACHVGGGTWTCTAAT3′). The PCR reaction contained a 25 μL mixture, including 10 μL of PCR Mix (Thermo Fisher, United States), 2 μL of template gDNA, 0.5 μL of each forward and reverse primer, and 12 μL of ddH_2_O. The conditions set for the PCR machine were: initial denaturation at 95°C for 3 min, followed by 32 cycles of denaturation at 94°C for 15 s, annealing at 55°C for 30 s, extension at 72°C for 30 s, and final extension at 72°C for 7 min. The PCR-amplified product was confirmed on a 1.5% agarose gel (470 bp) and purified using the QIAquick Gel Extraction Kit (Qiagen, Germany). Sequencing libraries were made with the Illumina Library Prep Kit (Illumina, United States), following company protocol, and their quality was evaluated with a Qubit 2.0 Fluorometer (Thermo Fisher, United States) before sequencing on an Illumina HiSeq 2000 sequencer. According to the Illumina quality filtering protocol, low-quality reads containing adaptors, primer dimers, reads shorter than 230 bp, and sequences with low-quality scores (≤ Q20 score) were removed. Qualified reads were trimmed using the Illumina pipeline version 2.6, following company guidelines.

The quality reads in each sample were clustered using the Mothur algorithm and the UPARSE drive5 pipeline, and OTUs were defined by clustering 97% of the identity sequences. We screened each OTU representative sequence for microbial taxonomy annotation. The Silva database 138 and the Mothur algorithm were used to annotate and differentiate Ruili, Nujiang, and Lianhe data at various taxonomic levels. We summarized the OTU relative abundance after normalizing it using the sample with the fewest sequences by standard sequence number. After normalizing, we examined the relative abundance of alpha and beta diversity indexes.

### Evaluating the alpha diversity index

In the phyloseq package, the Alpha Diversity Index (Chao1, ACE, and Simpson) was calculated using cumulative sum-scaling normalized values. ANOVA and post-hoc Tukey tests were used for alpha diversity indices to determine significant differences between groups. The cleaned data was converted into relative abundance. Good coverage was chosen to describe the depth of the sequencing.

### Evaluating the beta diversity index

We used the beta diversity index to compare the similarity of various samples. The beta diversity was calculated based on the two methods (weighted and unweighted UniFrac). Principal Component Analysis (PCA) and Principal Coordinate Analysis (PCoA) were used to reduce the original variable dimensions and to visualize complex, multi-dimensional data, apart from showing the relationships among microbial community structures. First, we calculated a distance matrix between samples using weighted or unweighted UniFrac methods, which were then transformed into orthogonal axes. PCoA demonstrated the highest variation factor, followed by the second, third, and so on. Then, Adonis analysis was performed to observe location-wise variations in the microbial structures. USEARCH software was used for significant difference analysis.

### Statistical analysis

Analyses in this study are reported as the standard error of the mean (SEM), and differences in relative bacterial abundance between groups were examined using Mann–Whitney sum rank tests. Data with a *p*-value (*p* < 0.05) were considered statistically significant.

## Results

### Bacterial composition in the *Rattus norvegicus* intestines

All the intestinal tissues collected from the free-living urban *R. norvegicus* were subjected to microbial profile analyses. A total of 1,229,810 raw reads (pair-end sequences) were generated for all the samples, with an average of 122,981 reads for each sample. After sequence assembly and quality control filtering (removing primer dimers and ambiguous and low-quality reads), 1,106,641 cleaned tags were obtained with an average Q30 score of >93%, which were used for subsequent analysis (Supplementary Table S1). The Chao1 diversity index curves for all the intestinal tissues reached a certain sequencing level, whereas the Simpson curves failed to level off (Supplementary Figure S1). These findings suggest that the abundance community structure has already been captured, though more microbial phenotypes can be explored with deeper sequencing in the future.

**Figure 1:**
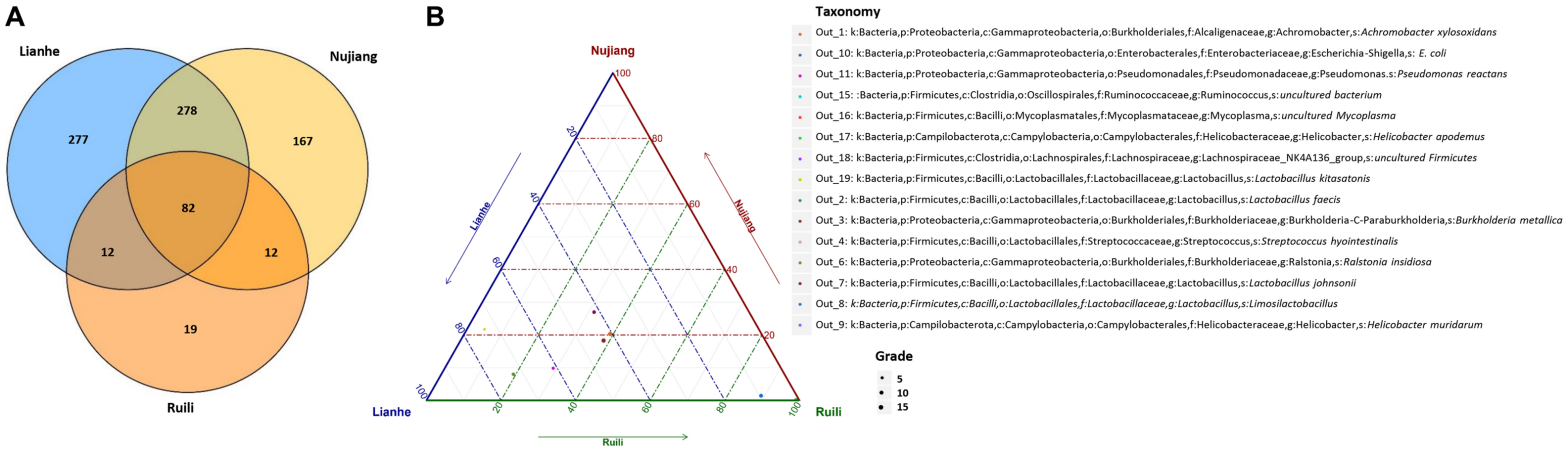
Clean tags from each *R. norvegicus* specimen were combined to generate OTUs for identifying differential microbial taxonomy levels. **(A)** The Venn diagram shows that among the 847 distinct OTUs, 19 were exclusive to the Ruili, 167 Nujiang, and 277 Lianhe groups, while 82 OTUs were shared. All these OTUs were then annotated to identify differential microbial taxonomy levels. **(B)** The top 15 OUTs abundance with different taxonomy levels.

Clean tags from each *R. norvegicus* intestinal tissue were combined to generate an Operational Taxonomic Unit (OTU) for identifying differential microbial taxonomy levels. Clustering at 97% identity produced 847 distinct OTUs, of which 19 OTUs were exclusive to the Ruili, 167 Nujiang, and 277 Lianhe areas, while 82 OTUs were shared among them (Figure 1A). All these OTUs were then annotated into 104 families, 168 genera, and 17 phyla. OTUs deep analysis revealed medically important opportunistic and highly pathogenic bacteria, including *Acinetobacter bereziniae* (OTU_227), *Bartonella australis* (OTU_23), *Burkholderia singularis* (OTU_1403), *Citrobacter koseri* (OTU_63), *Clostridium perfringens* (OTU_469), *Streptococcus hyointestinalis* (OTU_4), *Rickettsiella agriotidis* (OTU_1119), *Corynebacterium camporealensis* (OTU_805), *Mycoplasma haemomuris* (OTU_1089), *Corynebacterium pseudotuberculosis* (OTU_489), *Enterobacter cancerogenus* (OTU_32), *Streptococcus azizii* (OTU_139), *Streptococcus caballi* (OTU_240), *Pseudomonas mendocina* (OTU_269), *Vibrio vulnificus* (OTU_121), *Enterococcus faecalis* (OTU_189), *Escherichia coli* (OTU_10 and OTU_1436), etc., implying that rodents and their associated pathogens should be monitored regularly public health safety. However, the underlying pathogenic mechanisms of these bacteria need further investigation. The details about the OTU analysis and bacterial abundance at different taxonomic levels are shown in Figure 1B and Supplementary Table S2.

**Figure 2:**
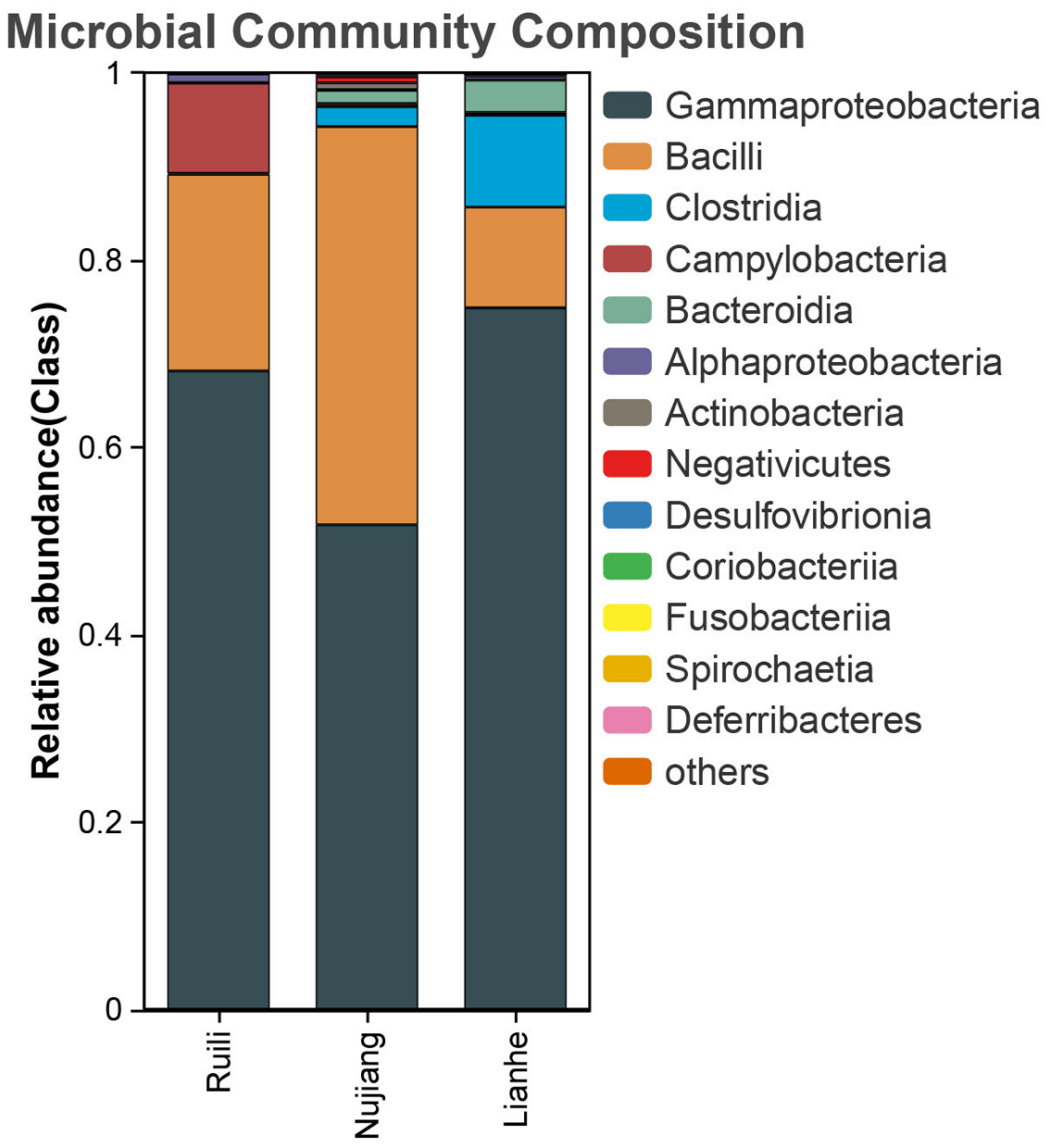
The top 14 relative abundance microbial class in the intestines of *R. norvegicus* collected from Ruili, Nujiang, and Lianhe regions.

**Figure 3:**
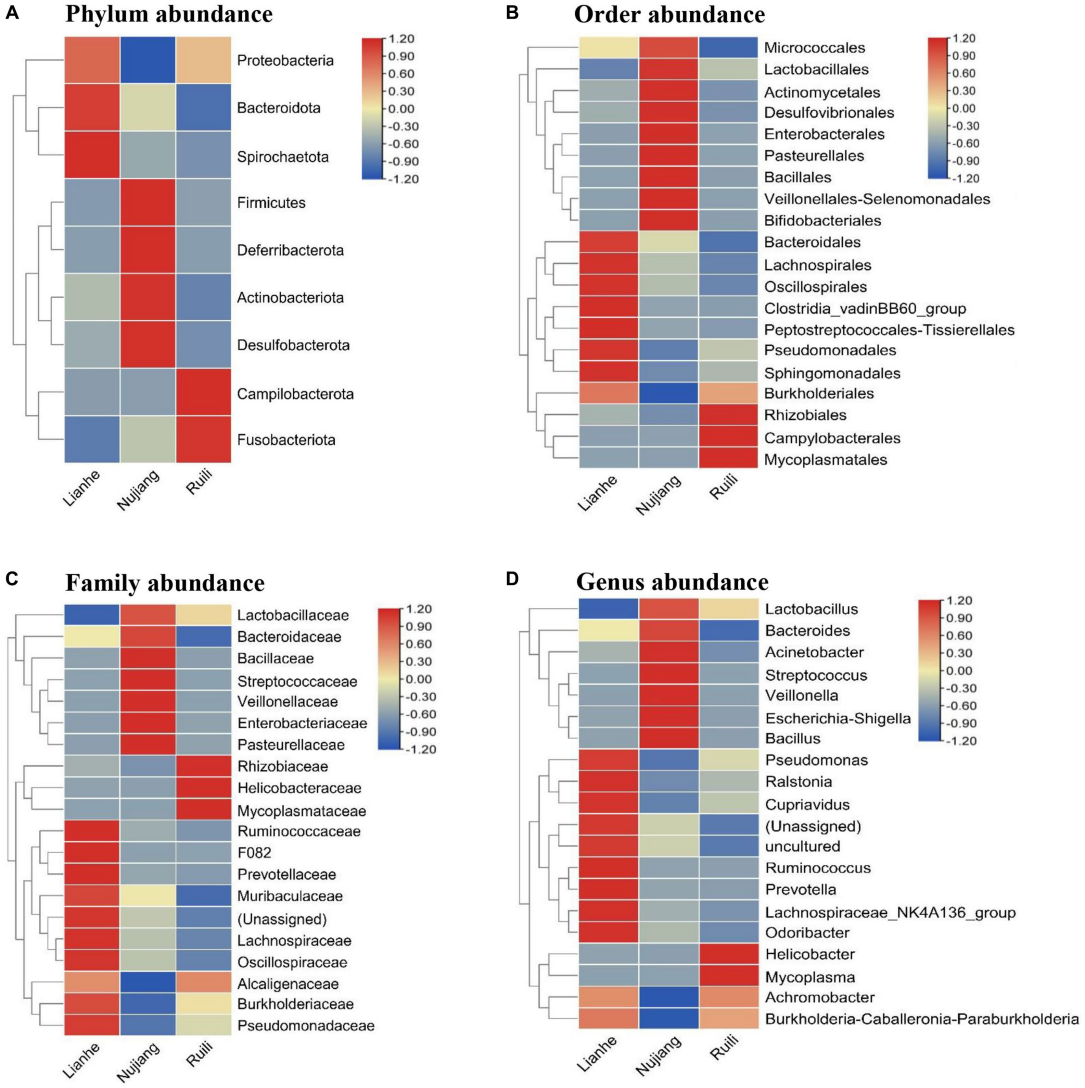
The relative microbial abundance at different taxonomic levels in the intestines of *R. norvegicus* collected from Lianhe, Nujiang and Ruili regions. **(A)** Microbial abundance at phylum level, **(B)** Microbial abundance at order level, **(C)** Microbial abundance at family level, and **(D)** Microbial abundance at genus level.

According to the relative abundance, Class Gammaproteobacteria, Bacilli, and Clostridia were the highest among the three groups (Figure 2). Following a similar trend, Gammaproteobacteria, Bacilli, and Clostridia were among the top bacterial classes in most samples (Supplementary Figure S2). The situation differed slightly for the Lianhe and Nujiang samples, although Phyla Bacteroidota and Spirochaetota were most prevalent. In contrast, the phylum Campilobacterota and Fusobacteriota were abundant in samples from Ruili. Overall, the highest number of phyla was reported in the Nujiang group (Figure 3A). Rhizobiales, Campylobacterales, and Mycoplasmatales were the richest order among the samples from Ruili, whereas Lactobacillales, Actinomycetales, and Desulfovibrionales were abundant in the Nujiang group (Figure 3B). Family-level classification showed Rhizobiaceae, Helicobacteraceae, and Mycoplasmataceae had the highest abundance in the Ruili group, whereas Lactobacillaceae, Bacteroidaceae, Bacillaceae, etc., were richest in the Nujiang group. Overall, the highest level of family abundance was reported in the Lianhe group and the lowest in the Ruili group (Figure 3C). Moreover, Genus *Helicobacter* and *Mycoplasma* were abundant in the Ruili group, whereas *Lactobacillus*, *Bacillus*, *Escherichia-Shigella*, etc., were richest in the Nujiang group (Figure 3D). The detailed values of the various taxonomic groups among the three groups are shown in Supplementary Table S3.

### Bacterial diversity analysis

**Figure 4:**
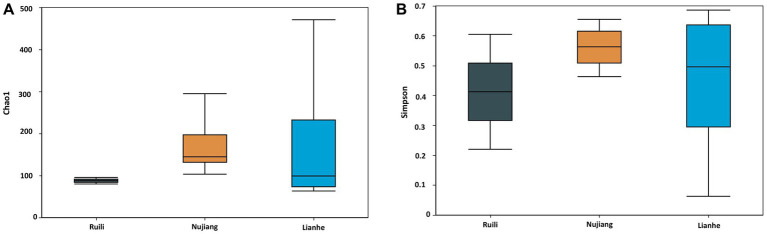
Chao 1 and Simpson indices represent the microbial abundance of different groups. **(A)** The Nujiang group had a significantly higher microbial species abundance than the Ruili and Lianhe. **(B)** The same pattern was observed for the Simpson indices, where Nujiang had higher bacterial richness and evenness compared to the other two groups.

The Alpha Diversity Index determined microbial diversity and richness differences among the *R. norvegicus* intestinal samples from various locations. The Chao1 and Simpson indexes revealed the richness of the bacterial community among the three groups. Both the Chao1 and Simpson indexes changed similarly across the three groups. Compared to Ruili, samples from Nujiang and Lianhe were the highest, with significantly more bacterial abundance (Figure 4A). The Simpson index revealed the highest bacterial abundance in the Nujiang group, followed by Lianhe and Ruili (Figure 4B). Compared to the other eight samples, the Chao1 diversity index revealed the highest bacterial richness in the sample (J04) from the Lianhe region. In contrast, samples H03 and P04 from Nujiang had almost no difference in bacterial richness (Supplementary Figure S3A). Moreover, the Simpson diversity index revealed bacterial community richness and evenness among samples from different demographics. Samples from Lianhe (L04) and Nujiang (N02) showed the highest bacterial richness (Supplementary Figure S3B). In contrast, sample J04 from the Lianhe had less bacterial richness compared to others in the same group.

### Variation in the bacterial communities

**Figure 5:**
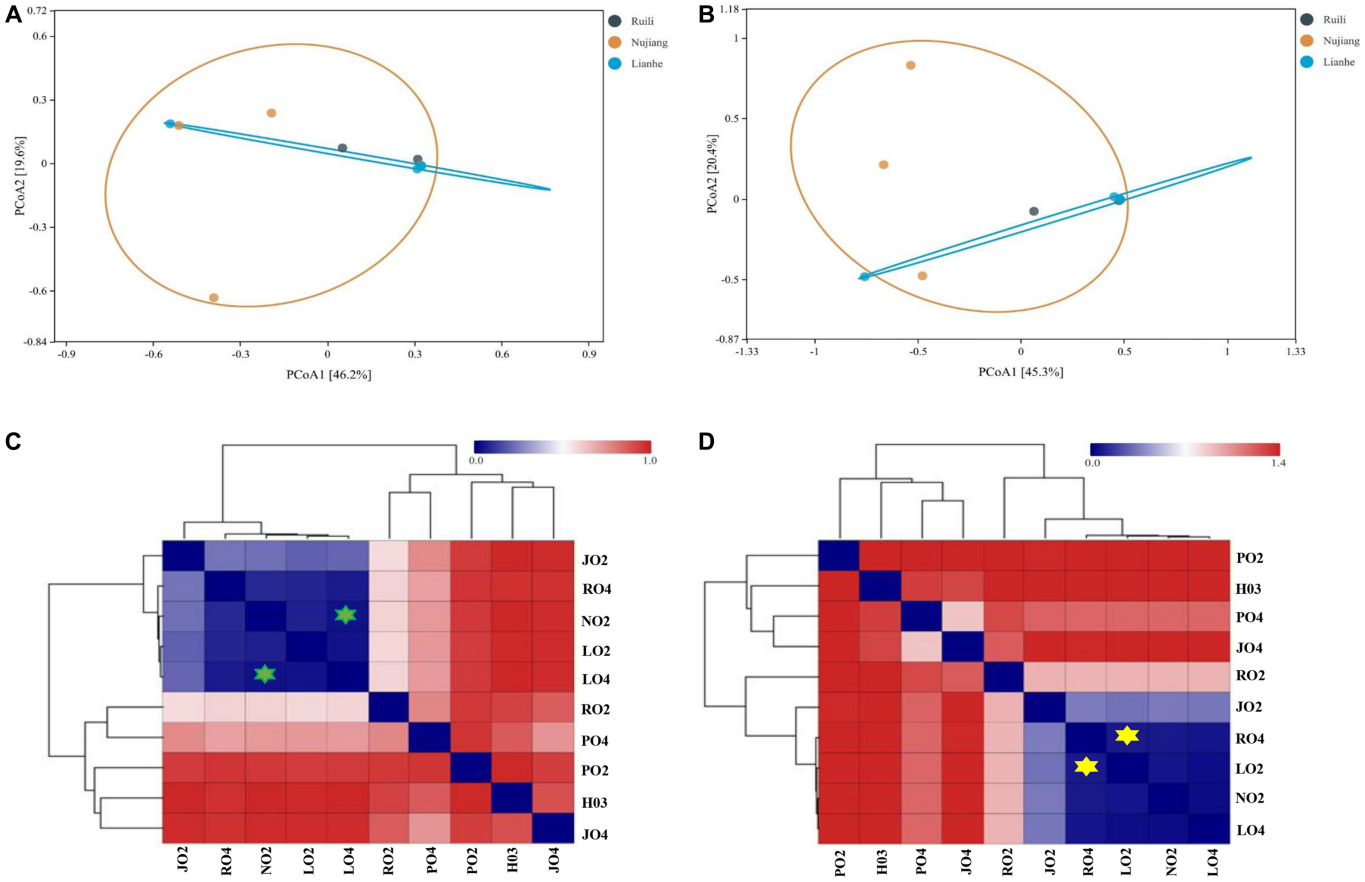
Principal Coordinate Analysis (PCoA) showing microbial differences in the intestines of *R. norvegicus* collected from Ruili, Nujiang, and Lianhe regions. **(A)** Microbial differences was determined using Bray Curtis distances matrix and **(B)** Euclidean distances matrix between samples from different locations. **(C)** Heatmap showing Bray Curtis distances between intestinal microbial communities **(D)** and Euclidean distances between *R. norvegicus* intestinal microbial communities.

Two distance metrics (Bray-Curtis and Euclidean) estimate microbial variation among different intestinal samples. A significant difference was observed in the *R. norvegicus* intestinal microbial communities among the three locations. PCoA visualized variation among the intestinal microbial community at various study sites. Using the Bray-Curtis distance, two PCoA plots coordinate percentage variation, i.e., PCoA1 (46.2.8%) and PCoA2 (19.6%) among the intestinal samples (Figure 5A). A slight variation was observed between the samples from different regions. In addition, the Euclidean distance metric estimates two PCoA plots for percentage variation: PCoA1 (45.3%) and PCoA2 (20.4%) among the intestinal samples (Figure 5B), implying that the variation may be attributed to differences in geographical locations. Heatmaps based on Bray-Curtis and Euclidean distances showed microbial variation among different intestinal samples. According to Bray-Curtis distance, the most similar lowest microbial difference was 0.0337, observed between samples N02 (Nujiang) and L04 (Lianhe), shown with green stars (Figure 5C), followed by 0.035 between L02 and L04 (samples from Lianhe), and 0.062 between L02 (Lianhe) and N02 (Nujiang), etc. (Supplementary Table S4). In addition, Euclidean distance showed similarity in the *R. norvegicus* intestinal microbial communities. The lowest difference (0.069) was observed between samples R04 (Ruili) and L02 (Lianhe), highlighted with yellow stars (Figure 5D), followed by R04 (Ruili) and N02 (Nujiang), suggesting that bacterial communities in these intestinal tissues were evolutionary similar. The detailed values of Euclidean distances are shown in Supplementary Table S5.

### Identification of potential OTU biomarkers

**Figure 6:**
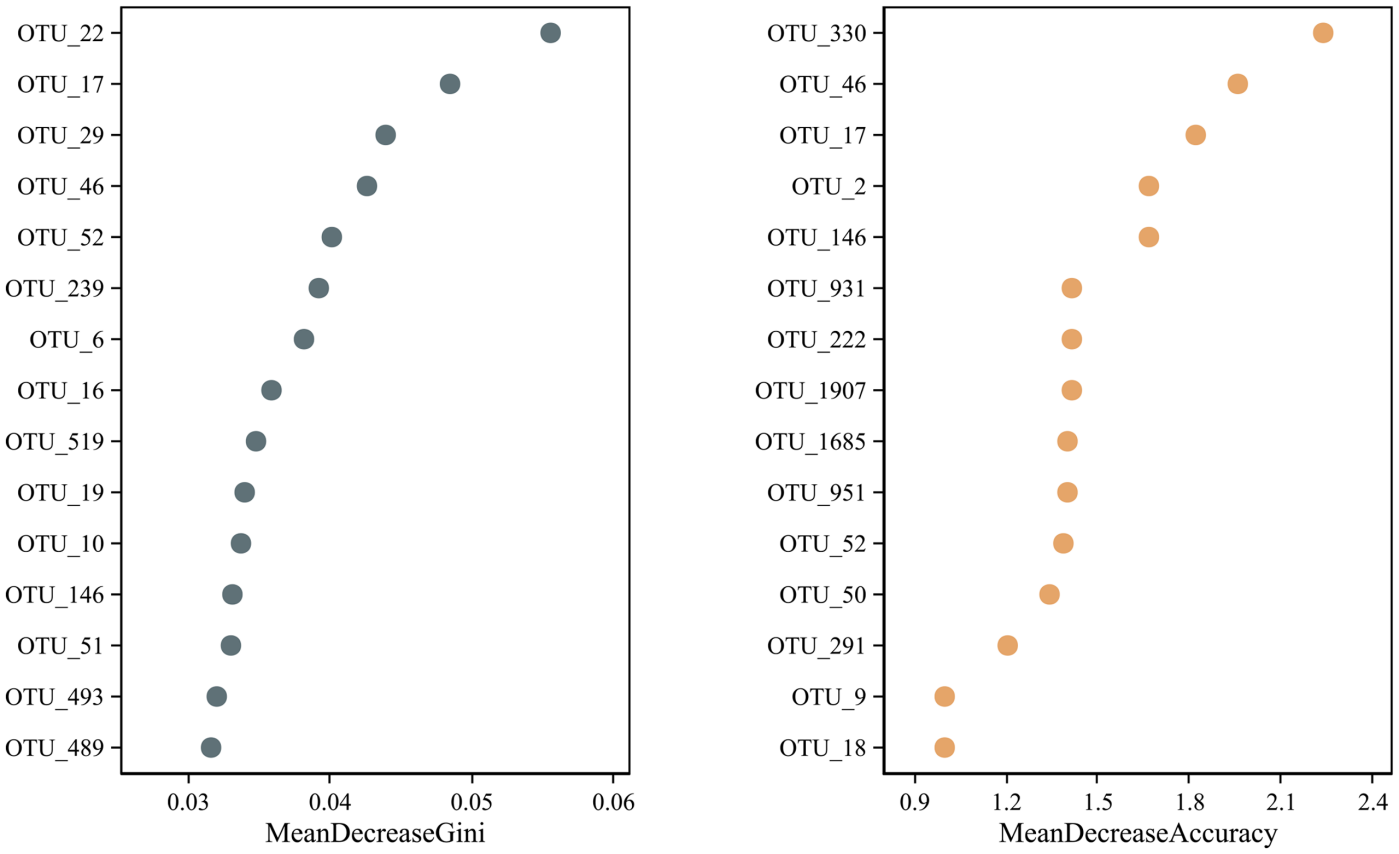
A random forest test identified fifteen potential OTU biomarkers in the intestines of *R. norvegicus*. The mean decrease in accuracy and the mean reduction in the Gini coefficient showed potential microbial OTU biomarkers in the *R. norvegicus* intestinal samples.

A random forest test identified potential microbial OTU biomarkers in the *R. norvegicus* intestinal samples. A random forest model cross-validation curve revealed 15 important OTU biomarkers (Supplementary Table S6). The mean decrease in accuracy and mean reduction in the Gini coefficient showed some important OTU distributions (Figure 6), validating potential microbial biomarkers in the *R. norvegicus* intestinal samples.

### Identification of potential bacterial pathogens

We screened and identified several opportunistic and potential bacterial pathogens in the intestines of *R. norvegicus* collected from the Ruili, Nujiang, and Lianhe regions. These pathogens were *Acinetobacter bereziniae*, associated with blood, urinary tract, and lung infections in humans. *Bartonella australis* was known to cause anemia syndrome and deaths in eastern grey kangaroos, whereas *Burkholderia singularis* causes cystic fibrosis in humans. *Citrobacter koseri* causes meningoencephalitis and multiple brain abscesses in humans. *Clostridium perfringens* is a famous anaerobic bacterial pathogen causing food poisoning, diarrhea, and gas gangrene in humans. Bacterial pathogens, *Corynebacterium camporealensis* and *Corynebacterium pseudotuberculosis,* are reportedly responsible for mastitis in sheep and necrotizing lymphadenitis in other animals. *Streptococcus azizii* and *Streptococcus caballi* cause meningoencephalitis, ventriculitis in mice, and laminitis in horses, respectively. *Pseudomonas mendocina* was associated with spinal infection in humans, whereas *Vibrio vulnificus* was linked with cholera in humans. *Enterococcus faecalis* and *E. coli* cause nosocomial infections and diarrhea in humans (Table 1).

**Table 1:** Shows some medically important bacterial pathogens with known pathogenesis.

Bacterial pathogens (this study)	Ruili	Nujiang	Lianhe	Disease descriptions	Reference database
*Acinetobacter bereziniae*	1.5	1.8	13.3	Opportunistic pathogens target blood, urinary tract, and lung in humans	BV-BRC
*Bacillus thuringiensis*	0.5	481	1.5	Opportunistic pathogen of animals (other than insects) causing necrosis and pulmonary infections	VFDB
*Bartonella australis*	729.5	0.8	0.5	Anemia syndrome and deaths of eastern grey kangaroos	BV-BRC
*Burkholderia singularis*	0	0	0.5	Cause meningoencephalitis and cystic fibrosis in humans	BV-BRC
*Citrobacter koseri*	301.5	41.5	2.8	Meningoencephalitis, multiple brain abscesses in humans	BV-BRC
*Clostridium perfringens*	2.5	6	4.5	Food poisoning, diarrhea, and gas gangrene in humans	VFDB
*Corynebacterium camporealensis*	3	0	0	Associated with mastitis in sheep	BV-BRC
*Corynebacterium pseudotuberculosis*	0	7.3	0	Necrotizing lymphadenitis in animals	VFDB
*Desulfovibrio fairfieldensis*	0	0	1.3	Damage intestinal epithelial barrier and activate intrinsic inflammation in humans	BV-BRC
*Enterococcus faecalis*	45	19.2	26.5	Nosocomial infection in humans	VFDB
*Escherichia coli*	60	16456.8	225.3	Cause diarrhea in humans	VFDB
*Mycoplasma haemomuris*	3.5	0	0	Cause anemia in rats and mice	BV-BRC
*Pseudomonas mendocina*	0	0.5	10.5	Cause spinal infection in humans	VFDB
*Streptococcus azizii*	4.5	15	30.8	Meningoencephalitis and ventriculitis in mice	BV-BRC
*Streptococcus caballi*	0	22.8	0	Associated with laminitis in horses	BV-BRC
*Vibrio vulnificus*	0	42.8	0	Intestinal disease (cholera) in humans	VFDB

The digits in the regional columns (i.e., Ruili, Nujiang, and Lianhe) represent the relative abundance of bacterial pathogens. VFDB: Virulence Factors of Pathogenic Bacteria Database (http://www.mgc.ac.cn/VFs/search_VFs.htm). BV-BRC: Bacterial and Viral Bioinformatics Resource Center (https://www.bv-brc.org/view/Bacteria/2)/EIsource.

## Discussion

Captive *R. norvegicus* is a preferred model in biomedical research for studying physiological parameters, behavior, and human diseases (Smith et al., 2019). The first *R. norvegicus* genomic sequence was published in 2004 (Gibbs et al., 2004) and yielded insights into mammalian evolution. It was the third sequenced genome after the human and the mouse. However, the rat intestinal microbiome has not been thoroughly studied, and our understanding of the microbial spatial structure remains limited. According to the authors, no previous study has investigated microbial community compositions and potential bacterial pathogens in the intestines of *R. norvegicus* collected from different regions of Yunnan Province. Therefore, we used high-throughput 16S rRNA gene sequencing to explore the intestinal microbial diversity of free-living *R. norvegicus*.

Our findings show that the intestinal microbiome of free-living urban *R. norvegicus* from the Ruili, Nujiang, and Lianhe regions was dominated by the phyla Proteobacteria and Firmicutes. The alpha and beta diversity compositions within groups differed significantly, which could be attributed to differences in sampling times, body sizes, and genders of the animals used. The gap between the Ruili, Nujiang, and Lianhe groups was more remarkable than within a group. Researchers have found significant differences in the bacterial compositions of *R. norvegicus* intestinal samples (He et al., 2020). In our study, Proteobacteria and Firmicutes were the two most abundant phyla in the intestines of *R. norvegicus*, which were consistent with previous findings from laboratory rats (Li et al., 2017), revealing a similar microbial profile in wild-type and laboratory rats; the phenomenon might be attributed to the host microbiota tropism. In addition, Lactobacillus constitutes a major component of the human intestines (Vemuri et al., 2018).

We found Lactobacillus abundance in the *R. norvegicus* intestines, consistent with the findings of He et al. (2020), who report abundant Lactobacillus in human guts, implying that *R. norvegicus* could be a possible experimental model for studying disease-related microbial alterations in humans (He et al., 2020). Firmicutes are the most common phylum in human digestive tracts, with Streptococcaceae being the most common family (Vemuri et al., 2018; Jiang et al., 2019). We discovered that the family Streptococcaceae was common within Firmicutes in *R. norvegicus* intestines, implying that the microbial composition of *R. norvegicus* and humans is strikingly similar. We found that the family Streptococcaceae was common within the phylum Firmicutes in the intestines, suggesting a remarkable similarity between *R. norvegicus* and the human microbiome structures.

*R. norvegicus* is a commensal synanthropic pest mammal species that lives primarily in forests and feeds on invertebrates. The rapid increase in human populations and industrialization have resulted in significant reductions in *R. norvegicus* natural habitats. As a result, this mammal is transforming into a commensal rodent, increasing opportunities for human-animal interaction and potential pathogen transmission to humans and other domestic mammals. *R. norvegicus* lives and feeds closer to humans, which may be a source of infectious diseases through cross-species transmission.

Our study annotated more sequences for notarizing, opportunistic, and highly pathogenic bacteria, particularly *S. azizii*, *S. ferus*, *S. caballi*, *Peptostreptococcus anaerobius*, *S. hyointestinalis*, *Peptostreptococcus anaerobius*, etc., in the *R. norvegicus* intestines. Therefore, effective disease prevention and rodent control measures should be taken at the animal-human interface. A better surveillance system is advised to further aid in the early detection of disease in humans and other domestic animals. The anaerobic spore-forming bacterium *Clostridium perfringens*, which was discovered in the guts of *R. norvegicus*, has been linked to acute gastrointestinal problems, necrotizing enterocolitis, and myonecrosis in humans (Yao and Annamaraju, 2023). Another *Clostridium celatum* isolated from human feces has been known to cause severe human infections (Agergaard et al., 2016). *Lactococcus lactis* subsp. hordniae identified in our study was previously involved in serious diseases such as endocarditis, peritonitis, and intra-abdominal infections in humans (Lahlou et al., 2023). A bacterial species, *Lactococcus garvieae*, appears to correlate with seasonal aquaculture outbreaks in humans (Wang et al., 2007). *Mycoplasma haemomuris* is a gram-negative extracellular obligate parasite of rat erythrocytes. This bacterial infection has been shown to affect tumor kinetics, reticuloendothelial function, erythrocyte shelf life, interferon production, and host response to viral and protozoal infections (Otto et al., 2015). We identified two genus *Escherichia-Shigella* in the *R. norvegicus* intestines, reportedly responsible for human gastrointestinal disorders (Belotserkovsky and Sansonetti, 2018). The commensal species *Neisseria subflava* identified in our current study is a rare cause of invasive diseases like meningitis, endocarditis, bacteremia, pericarditis, and septic arthritis in humans (Uwamino et al., 2017). *S. azizii* (associated with meningoencephalitis in newborn weanling C57BL/6 mice) was named in honor of American microbiologist Aziz, who provided many years of his life supporting Memorial Sloan Kettering Cancer Center in New York, United States (Braden et al., 2015). *Corynebacterium* species are facultative intracellular bacilli that can cause pneumonia and lower respiratory tract infections (Marques da Silva et al., 2021). The *Corynebacterium pseudotuberculosis* reported in this study is known for causing caseous lymphadenitis in ruminants, mastitis in dairy cattle, lymphangitis in horses, and edema in buffaloes (Marques da Silva et al., 2021). These findings indicate that *R. norvegicus* may transmit potential infectious pathogens to humans and other mammals via contaminated food and water. A study found a high prevalence of Bartonella in the blood of *R. norvegicus*, indicating that these rodents may transmit infectious diseases, particularly Bartonellosis and rat-bite fever, to humans and domestic animals (Hsieh et al., 2010). We identified *Bartonella australis* in the *R. norvegicus* intestines, indicating that Bartonella may pass from the rodent via excrement. In Guangdong Province, China, in 2019, a patient who had been bitten by a wild rat one week prior to the onset of rat-bite fever (caused by *Streptobacillus moniliformis*) symptoms (Mai et al., 2019). We also identified the same bacterial species, *Streptobacillus moniliformis*, in the *R. norvegicus* intestines, indicating a risk of transmitting the same pathogen to humans in light of the possibility that rat-bite fever could be transmitted by *R. norvegicus*, suggesting strict disease prevention strategies regarding pathogen transmission (Mai et al., 2019).

Our current study has several limitations. The first limitation of this study was that we did not identify the age and gender of the free-living urban rat species *R. norvegicus*, even though age and gender may affect microbial community structures in the intestines of these important rodents. Second, the sample size in our current study was small, so more samples are needed to investigate the diverse microbial community compositions in each segment of the digestive tract. Third, we did not examine how season, age, gender, diet, etc., affect microbiome profiles; thus, larger sample sizes in future studies should confirm our findings.

## Conclusion

In conclusion, we reported the intestinal microbial compositions of free-living *R. norvegicus*. We also revealed pathogenic bacteria in the intestinal samples of *R. norvegicus*, raising the possibility of these important rodents being exposed to humans and animals. Although intestinal samples cannot represent the entire microbiome of the digestive tract, future research must ensure sampling from every segment of the digestive tract to explore deep microbial profiles and their role in gut dysbiosis and disease progression.

## Data availability statement

The datasets presented in this study can be found in online repositories. The names of the repository/repositories and accession number(s) can be found below: NCBI – SRP463903.

## Ethics statement

The animal study was approved by the experiment was comconducted according to the guidelines for the use of laboratory animals, Faculty of Life Science and Technology, Kunming University of Science and Technology, Yunnan, China (protocol no. 16048). The study was conducted in accordance with the local legislation and institutional requirements.

## Author contributions

TS: Conceptualization, Writing – original draft, Writing – review & editing. YH: Writing – original draft. JJ: Writing – original draft. ZS: Writing – original draft. YuW: Writing – original draft. QL: Writing – original draft. XiX: Writing – original draft. YiW: Writing – original draft. BW: Conceptualization, Writing – original draft. XuX: Writing – original draft, Conceptualization, Funding acquisition, Supervision, Writing – review & editing.
