# Supplementary material for: Comparative analysis of the intestinal microbiome in Rattus norvegicus from different geographies
Source: Front Microbiol. 2023 Nov 3;14:1283453. doi: 10.3389/fmicb.2023.1283453 (PMC10655115; doi:10.3389/fmicb.2023.1283453)
Supplement: Supplementary file 1 [file Data_Sheet_1.docx]

**Table S1. Assembly and quality filtered cleaned tags obtained for downstream analyses**

| **Sample IDs** | **Locations** | **Total raw reads** | **Total clean tags** | **Q30 (%)** |
| --- | --- | --- | --- | --- |
| RO2 | Ruili | 123,004 | 110,199 | 94.3 |
| RO4 | Ruili | 120,208 | 111,299 | 94.1 |
| NO2 | Nujiang | 127,972 | 116,550 | 93.9 |
| PO2 | Nujiang | 129,972 | 116,810 | 94.3 |
| PO4 | Nujiang | 126,515 | 110,710 | 92.4 |
| H03 | Nujiang | 124,997 | 108,078 | 91.7 |
| LO2 | Lianhe | 126,424 | 116,242 | 93.8 |
| LO4 | Lianhe | 97,863 | 89,482 | 94.3 |
| JO2 | Lianhe | 122,749 | 109,823 | 93.2 |
| JO4 | Lianhe | 130,106 | 117,448 | 93.6 |
| **Total** | **--** | **1,229,810** | **1,106,641** | **93.56 (avg.)** |

**Table S2. Bacterial taxonomies among all the OTUs identified in this study**

| **OTU ID** | **Ruili** | **Nujiang** | **Lianhe** | **Taxonomy** |
| --- | --- | --- | --- | --- |
| OTU_100 | 101.5 | 0 | 8 | k:Bacteria,p:Firmicutes,c:Bacilli,o:Mycoplasmatales,f:Mycoplasmataceae,g:Ureaplasma,s:uncultured |
| OTU_1004 | 0 | 1 | 0.75 | k:Bacteria,p:Actinobacteriota,c:Coriobacteriia,o:Coriobacteriales,f:Eggerthellaceae,g:Enterorhabdus,s:uncultured |
| OTU_1005 | 0 | 0.25 | 1.25 | k:Bacteria,p:Firmicutes,c:Clostridia,:o:Borkfalkiales,f:uncultured |
| OTU_1006 | 0 | 0.5 | 2.75 | k:Bacteria,p:Actinobacteriota,c:Actinobacteria,o:Micrococcales,f:Brevibacteriaceae,g:Brevibacterium,s:uncultured |
| OTU_1007 | 0 | 0 | 2.25 | k:Bacteria,p:Proteobacteria,c:Gammaproteobacteria,o:Pseudomonadales,f:Moraxellaceae,g:Enhydrobacter,s:Moraxella osloensis |
| OTU_1011 | 0 | 0.75 | 1.5 | k:Bacteria,p:Bacteroidota,c:Bacteroidia,o:Bacteroidales,f:Muribaculaceae,g:uncultured |
| OTU_101 | 90.5 | 1 | 2 | k:Bacteria,p:Fusobacteriota,c:Fusobacteriia,o:Fusobacteriales,f:Fusobacteriaceae,g:Fusobacterium,s:uncultured |
| OTU_1012 | 0 | 0 | 1 | k:Bacteria,p:Firmicutes,c:Clostridia,o:Oscillospirales,f:Oscillospiraceae,g:uncultured |
| OTU_1013 | 0 | 0 | 0.75 | k:Bacteria,p:Firmicutes,c:Clostridia,o:Clostridiales,f:Oscillospiraceae,g:uncultured |
| OTU_1016 | 0 | 0 | 1.25 | k:Bacteria,p:Firmicutes,c:Clostridia,o:Lachnospirales,f:Lachnospiraceae,g:uncultured |
| OTU_1017 | 0 | 0.75 | 35.5 | k:Bacteria,p:Firmicutes,c:Clostridia,o:Lachnospirales,f:Lachnospiraceae,g:NK4A136,s:uncultured |
| OTU_1018 | 0 | 0 | 1.5 | k:Bacteria,p:Proteobacteria,c:Alphaproteobacteria,o:Rickettsiales,f:uncultured |
| OTU_1019 | 0 | 3.25 | 0.5 | k:Bacteria,p:Firmicutes,c:Clostridia,o:Oscillospirales,f:Oscillospiraceae,g:UCG-005,s:uncultured |
| OTU_1022 | 3 | 0 | 0 | k:Bacteria,p:Firmicutes,c:Clostridia,o:Peptostreptococcales-Tissierellales,g:Peptoniphilus,s:uncultured |
| OTU_1029 | 0 | 1.5 | 0 | k:Bacteria,p:Firmicutes,c:Clostridia,o:Lachnospirales,f:Lachnospiraceae,g:Lachnospiraceae ND3007,s:uncultured |
| OTU_102 | 4.5 | 4.75 | 42 | k:Bacteria,p:Proteobacteria,c:Alphaproteobacteria,o:Rhizobiales,f:Rhizobiaceae,g:Mesorhizobium,s:Mesorhizobium |
| OTU_103 | 0 | 73 | 12.75 | k:Bacteria,p:Firmicutes,c:Clostridia,o:Lachnospirales,f:Lachnospiraceae,g:PAC001103 |
| OTU_1032 | 0 | 4 | 0 | k:Bacteria,p:Proteobacteria,c:Gammaproteobacteria,o:Burkholderiales,f:Neisseriaceae,g:Neisseria,s:Neisseria subflava |
| OTU_1033 | 2 | 2 | 4 | k:Bacteria,p:Proteobacteria,c:Betaproteobacteria,o:Burkholderiales,f:Alcaligenaceae,g:Achromobacter,s:Achromobacter insuavis |
| OTU_1035 | 0 | 1 | 0 | k:Bacteria,p:Acidobacteriota,c:Holophagae,o:Subgroup 7 |
| OTU_10 | 60 | 16456.75 | 225.25 | k:Bacteria,p:Proteobacteria,c:Gammaproteobacteria,o:Enterobacterales,f:Enterobacteriaceae,g:Escherichia-Shigella,s:E. coli |
| OTU_1039 | 0 | 4.5 | 0 | k:Bacteria,p:Bacteroidota,c:Bacteroidia,o:Bacteroidales,f:Muribaculaceae,g:RIAY |
| OTU_1043 | 0 | 0.25 | 64.25 | k:Bacteria,p:Firmicutes,c:Clostridia,o:Clostridiale,f:uncultured |
| OTU_1049 | 0 | 1.5 | 0 | k:Bacteria,p:Bacteroidota,c:Bacteroidia,o:Bacteroidales,f:Muribaculaceae,g:PAC000198 |
| OTU_105 | 0 | 39 | 58.5 | k:Bacteria,p:Firmicutes,c:Clostridia,o:Oscillospirales,f:Oscillospiraceae,g:uncultured |
| OTU_106 | 0 | 47.5 | 0.5 | k:Bacteria,p:Proteobacteria,c:Gammaproteobacteria,o:Burkholderiales,f:Alcaligenaceae,g:Pelistega,s:Pelistega suis |
| OTU_1062 | 0 | 0 | 0.75 | k:Bacteria,p:Actinobacteriota,c:Actinobacteria,o:Corynebacteriales,f:Nocardiaceae,g:Gordonia |
| OTU_1067 | 0 | 0 | 1.5 | k:Bacteria,p:Firmicutes,c:Clostridia,o:Lachnospirales,f:Lachnospiraceae,g:Acetatifactor,s:uncultured |
| OTU_1069 | 0 | 0 | 0.75 | k:Bacteria,p:Firmicutes,c:Clostridia,o:Clostridiales,f:Oscillospiraceae,g:Oscillibacter,g:uncultured |
| OTU_1070 | 0 | 0.5 | 2.5 | k:Bacteria,p:Firmicutes,c:Clostridia,o:Oscillospirales,f:Oscillospiraceae,g:Oscillibacter,s:uncultured |
| OTU_107 | 0 | 54 | 0 | k:Bacteria,p:Proteobacteria,c:Gammaproteobacteria,o:Pasteurellales,f:Pasteurellaceae,g:Actinobacillus,s:Actinobacillus porcitonsillarum |
| OTU_1072 | 0 | 0 | 0.75 | k:Bacteria,p:Firmicutes,c:Clostridia,o:Lachnospirales,f:Lachnospiraceae,g:uncultured |
| OTU_1075 | 0 | 0 | 3.75 | k:Bacteria,p:Firmicutes,c:Clostridia,o:Oscillospirales,f:Oscillospiraceae,g:UCG-005 |
| OTU_1077 | 4 | 1.75 | 0 | k:Bacteria,p:Firmicutes,c:Bacilli,o:Lactobacillales,f:Leuconostocaceae,g:Weissella,s:Weissella confusa |
| OTU_1080 | 0 | 1.25 | 0 | k:Bacteria,p:Firmicutes,c:Clostridia,o:Oscillospirales,f:UCG-010 |
| OTU_108 | 0 | 83.75 | 0 | k:Bacteria,p:Firmicutes,c:Bacilli,o:Bacillales,f:Planococcaceae,g:Solibacillus,s:Solibacillus isronensis |
| OTU_1083 | 0 | 0.75 | 0 | k:Bacteria,p:Bacteroidota,c:Bacteroidia,o:Bacteroidales,f:Marinifilaceae,g:Butyricimonas,s:Butyricimonas synergistica |
| OTU_1089 | 3.5 | 0 | 0 | k:Bacteria,p:Firmicutes,c:Bacilli,o:Mycoplasmatales,f:Mycoplasmataceae,g:Mycoplasma,s:Mycoplasma haemomuris |
| OTU_1098 | 5.5 | 0 | 0 | k:Bacteria,p:Proteobacteria,c:Alphaproteobacteria,o:Sphingomonadales,f:Sphingomonadaceae,g:Blastomonas,s:Blastomonas natatoria |
| OTU_1107 | 0 | 1.75 | 0 | k:Bacteria,p:Bacteroidota,c:Bacteroidia,o:Bacteroidales,f:Prevotellaceae,g:Prevotella,s:uncultured |
| OTU_1117 | 0 | 3.25 | 0 | k:Bacteria,p:Actinobacteriota,c:Actinobacteria,o:Corynebacteriales,f:Corynebacteriaceae,g:Corynebacterium,s:Corynebacterium casei |
| OTU_1119 | 0 | 0 | 1 | k:Bacteria,p:Proteobacteria,c:Gammaproteobacteria,o:Diplorickettsiales,f:Diplorickettsiaceae,g:Rickettsiella,s:Rickettsiella agriotidis |
| OTU_1122 | 0 | 1 | 1.75 | k:Bacteria,p:Bacteroidota,c:Bacteroidia,o:Bacteroidales,f:Rikenellaceae,g:Rikenellaceae RC9,s:Uncultured |
| OTU_11 | 1371.5 | 486.75 | 2898 | k:Bacteria,p:Proteobacteria,c:Gammaproteobacteria,o:Pseudomonadales,f:Pseudomonadaceae,g:Pseudomonas.s:Pseudomonas reactans |
| OTU_1128 | 0 | 0 | 1.25 | k:Bacteria,p:Acidobacteriota,c:Acidobacteriae,o:Acidobacteriales,f:uncultured,g:uncultured |
| OTU_113 | 0.5 | 13.75 | 86.25 | k:Bacteria,p:Firmicutes,c:Clostridia,o:Oscillospirales,f:Oscillospiraceae,g:Colidextribacter,s:uncultured |
| OTU_1133 | 0 | 2 | 5.75 | k:Bacteria,p:Bacteroidota,c:Bacteroidia,o:Bacteroidales,f:Marinifilaceae,g:Odoribacter,s:Bacteroides splanchnicus |
| OTU_1134 | 1.5 | 0 | 0 | k:Bacteria,p:Proteobacteria,c:Gammaproteobacteria,o:Burkholderiales,f:Burkholderiaceae,g:Paraburkholderia,s:Paraburkholderia hayleyella |
| OTU_1137 | 0 | 1.75 | 0 | k:Bacteria,p:Firmicutes,c:Clostridia,o:Oscillospirales,f:Ruminococcaceae,g:Ruminococcus,s:Ruminococcoides bili |
| OTU_114 | 0 | 1.5 | 62 | k:Bacteria,p:Firmicutes,c:Clostridia,o:Oscillospirales,f:Oscillospiraceae,g:NK4A214 |
| OTU_1141 | 0 | 0 | 2.25 | k:Bacteria,p:Proteobacteria,c:Gammaproteobacteria,o:Oceanospirillales,f:Halomonadaceae,g:Halomonas,s:Halomonas campaniensis |
| OTU_1143 | 0 | 2.25 | 0 | k:Bacteria,p:Firmicutes,c:Clostridia,o:Lachnospirales,f:Lachnospiraceae,g:Acetitomaculum,s:uncultured |
| OTU_1145 | 0 | 0 | 3.5 | k:Bacteria,p:Actinobacteriota,c:Thermoleophilia,o:Gaiellales |
| OTU_1149 | 3 | 0 | 0 | k:Bacteria,p:Chloroflexi,c:Chloroflexia,o:Chloroflexales,f:Roseiflexaceae,g:uncultured |
| OTU_1159 | 0 | 1 | 0 | k:Bacteria,p:Proteobacteria,c:Alphaproteobacteria,o:Paracaedibacterales,f:Paracaedibacteraceae,g:uncultured |
| OTU_116 | 0 | 0.75 | 60 | k:Bacteria,p:Firmicutes,c:Clostridia,o:Lachnospirales,f:Lachnospiraceae,g:uncultured |
| OTU_1162 | 0 | 0 | 3 | k:Bacteria,p:Firmicutes,c:Clostridia,o:Borkfalkiales,f:Borkfalkiaceae,g:PAC001141 |
| OTU_1168 | 1.5 | 0 | 0 | k:Bacteria,p:Proteobacteria,c:Alphaproteobacteria,o:Rhizobiales,f:Beijerinckiaceae,g:Methylobacterium,s:Methylobacterium SY-2 |
| OTU_1169 | 0 | 2.5 | 0 | k:Bacteria,p:Firmicutes,c:Clostridia,o:Lachnospirales,f:Lachnospiraceae,g:uncultured |
| OTU_1171 | 0 | 0 | 2 | k:Bacteria,p:Firmicutes,c:Clostridia,o:Lachnospirales,f:Lachnospiraceae,g:Kineothrix,s:Kineothrix alysoides |
| OTU_117 | 1 | 7.75 | 127.5 | k:Bacteria,p:Firmicutes,c:Clostridia,o:Lachnospirales,f:Lachnospiraceae,g:[Eubacterium] xylanophilum |
| OTU_1178 | 0 | 1 | 14.75 | k:Bacteria,p:Firmicutes,c:Clostridia,o:Lachnospirales,f:Lachnospiraceae,g:Lachnospiraceae NK4A136 |
| OTU_1182 | 0 | 3.25 | 0.25 | k:Bacteria,p:Firmicutes,c:Clostridia,o:Oscillospirales,f:[Eubacterium] coprostanoligenes group |
| OTU_1186 | 0 | 0 | 2.75 | k:Bacteria,p:Firmicutes,c:Clostridia,o:Borkfalkiales,f:PAC001219,g:GQ448104 |
| OTU_1190 | 0 | 2 | 2.5 | k:Bacteria,p:Firmicutes,c:Clostridia,o:Oscillospirales,f:Oscillospiraceae,g:Oscillibacter,s:uncultured |
| OTU_119 | 0 | 14.25 | 91.5 | k:Bacteria,p:Firmicutes,c:Clostridia,o:Lachnospirales,f:Lachnospiraceae,g:Lachnospiraceae NK4A136,s:uncultured |
| OTU_1192 | 0 | 2.5 | 2.25 | k:Bacteria,p:Firmicutes,c:Clostridia,o:Lachnospirales,f:Lachnospiraceae,g:Lachnospiraceae NK4A136,s:uncultured |
| OTU_1196 | 0 | 2 | 0 | k:Bacteria,p:Firmicutes,c:Clostridia,o:Lachnospirales,f:Lachnospiraceae,g:Velocimicrobium,s:Acetivibrio ethanolgignens |
| OTU_1197 | 0 | 4.75 | 3 | k:Bacteria,p:Bacteroidota,c:Bacteroidia,o:Bacteroidales,f:Muribaculaceae,g:RIAY |
| OTU_1199 | 0 | 0 | 0.5 | k:Bacteria,p:Firmicutes,c:Clostridia,o:Lachnospirales,f:Lachnospiraceae,g:PAC002367 |
| OTU_120 | 0 | 0.25 | 36.25 | k:Bacteria,p:Firmicutes,c:Clostridia,o:Oscillospirales,f:Ruminococcaceae,g:Ruminococcus,s:uncultured |
| OTU_1203 | 0 | 0 | 0.75 | k:Bacteria,p:Firmicutes,c:Clostridia,o:Lachnospirales,f:Lachnospiraceae,g:Hominifimenecus,s:uncultured |
| OTU_1205 | 0 | 0 | 0.75 | k:Bacteria,p:Firmicutes,c:Clostridia,o:Clostridiales,f:Lachnospiraceae,g:PAC001118,s:uncultured |
| OTU_1206 | 0 | 0 | 3.75 | k:Bacteria,p:Firmicutes,c:Clostridia,o:Clostridiale,f:Oscillospiraceae,g:uncultured |
| OTU_1207 | 0 | 1 | 0 | k:Bacteria,p:Actinobacteriota,c:Coriobacteriia,o:Coriobacteriales,f:Eggerthellaceae,g:Enterorhabdus,s:uncultured |
| OTU_1208 | 3.5 | 7.5 | 5.5 | k:Bacteria,p:Firmicutes,c:Bacilli,o:Lactobacillales,f:Enterococcaceae,g:Enterococcus,s:uncultured |
| OTU_121 | 0 | 42.75 | 0 | k:Bacteria,p:Proteobacteria,c:Gammaproteobacteria,o:Vibrionales,f:Vibrionaceae,g:Vibrio,s:Vibrio vulnificus |
| OTU_1211 | 0 | 0 | 0.75 | k:Bacteria,p:Firmicutes,c:Clostridia,o:Oscillospirales,f:Oscillospiraceae,g:uncultured |
| OTU_1212 | 0 | 0 | 1.25 | k:Bacteria,p:Firmicutes,c:Clostridia,o:Clostridiales,f:Oscillospiraceae,g:Flintibacter,s:Flintibacter butyricus |
| OTU_1213 | 0 | 0 | 2.25 | k:Bacteria,p:Firmicutes,c:Clostridia,o:Oscillospirales,f:Ruminococcaceae,g:uncultured |
| OTU_1 | 50588 | 27488 | 52083 | k:Bacteria,p:Proteobacteria,c:Gammaproteobacteria,o:Burkholderiales,f:Alcaligenaceae,g:Achromobacter,s:Achromobacter xylosoxidans |
| OTU_1216 | 0 | 0 | 1.75 | k:Bacteria,p:Firmicutes,c:Clostridia,o:Borkfalkiales,f:PAC001219,g:GQ451249 |
| OTU_1218 | 0 | 3.5 | 1.25 | k:Bacteria,p:Firmicutes,c:Clostridia,o:Lachnospirales,f:Lachnospiraceae,g:Lachnospiraceae NK4A136,s:uncultured |
| OTU_1219 | 0 | 0 | 1 | k:Bacteria,p:Firmicutes,c:Bacilli,o:Lactobacillales,f:Lactobacillaceae,g:Lactobacillus,s:Ligilactobacillus faecis |
| OTU_1220 | 0 | 0.5 | 0 | k:Bacteria,p:Proteobacteria,c:Gammaproteobacteria,o:Enterobacterales,f:Enterobacteriaceae,g:Entomohabitans,s:Entomohabitans teleogrylli |
| OTU_122 | 0 | 2.25 | 99 | k:Bacteria,p:Firmicutes,c:Clostridia,o:Borkfalkiales,f:PAC001219,g:PAC001219 |
| OTU_1221 | 0 | 3.25 | 0 | k:Bacteria,p:Bacteroidota,c:Bacteroidia,o:Bacteroidales,f:Muribaculaceae,g:PAC001692 |
| OTU_1229 | 1.5 | 5 | 55.75 | k:Bacteria,p:Firmicutes,c:Bacilli,o:Lactobacillales,f:Lactobacillaceae,g:Lactobacillus,s:uncultured |
| OTU_1232 | 0 | 0 | 0.5 | k:Bacteria,p:Firmicutes,c:Clostridia,o:Clostridiales,f:Oscillospiraceae,g:uncultured |
| OTU_1234 | 0 | 0 | 0.5 | k:Bacteria,p:Firmicutes,c:Clostridia,o:Lachnospirales,f:Lachnospiraceae,g:Herbinix,s:uncultured |
| OTU_1236 | 0 | 2.75 | 0.5 | k:Bacteria,p:Proteobacteria,c:Gammaproteobacteria,o:Pseudomonadales,f:Pseudomonadaceae,g:Stutzerimonas,s:Stutzerimonas nitrititolerans |
| OTU_1240 | 0 | 1 | 1.75 | k:Bacteria,p:Firmicutes,c:Clostridia,o:Lachnospirales,f:Lachnospiraceae,g:uncultured |
| OTU_124 | 1 | 0.25 | 81.25 | k:Bacteria,p:Firmicutes,c:Clostridia,o:Borkfalkiales,f:Borkfalkiaceae,g:PAC001141 |
| OTU_1244 | 0 | 0 | 3 | k:Bacteria,p:Firmicutes,c:Clostridia,o:Lachnospirales,f:Lachnospiraceae,g:uncultured |
| OTU_125 | 0.5 | 0.25 | 80.25 | k:Bacteria,p:Firmicutes,c:Clostridia,o:Lachnospirales,f:Lachnospiraceae,g:Lachnospiraceae NK4A136,s:uncultured |
| OTU_1251 | 5 | 0.25 | 0 | k:Bacteria,p:Actinobacteriota,c:Coriobacteriia,o:Coriobacteriales,f:Coriobacteriaceae,g:Collinsella,s:Collinsella marseille |
| OTU_1253 | 0 | 0 | 2.5 | k:Bacteria,p:Firmicutes,c:Clostridia,o:Clostridiales,f:Oscillospiraceae,g:Flintibacter,s:uncultured |
| OTU_1254 | 0 | 0.75 | 153.75 | k:Bacteria,p:Firmicutes,c:Clostridia,o:Lachnospirales,f:Lachnospiraceae,g:PAC001588 |
| OTU_1255 | 0 | 0 | 2.5 | k:Bacteria,p:Firmicutes,c:Clostridia,o:Lachnospirales,f:Lachnospiraceae,g:uncultured |
| OTU_1256 | 0 | 1 | 3.75 | k:Bacteria,p:Bacteroidota,c:Bacteroidia,o:Bacteroidales,f:Muribaculaceae,g:RIAY |
| OTU_1260 | 0 | 0 | 1 | k:Bacteria,p:Firmicutes,c:Clostridia,o:Clostridiales,f:Lachnospiraceae,g:uncultured |
| OTU_126 | 0 | 0 | 40.75 | k:Bacteria,p:Bacteroidota,c:Bacteroidia,o:Bacteroidales,f:Bacteroidaceae,g:Bacteroides,s:uncultured |
| OTU_1266 | 0 | 0 | 2.75 | k:Bacteria,p:Firmicutes,c:Clostridia,o:Clostridia UCG-014 |
| OTU_1268 | 0 | 5.5 | 12 | k:Bacteria,p:Firmicutes,c:Clostridia,o:Oscillospirales,f:Ruminococcaceae,g:Incertae Sedis |
| OTU_127 | 0 | 1.5 | 32.25 | k:Bacteria,p:Bacteroidota,c:Bacteroidia,o:Bacteroidales,f:Muribaculaceae,g:PAC000198 |
| OTU_1274 | 0 | 0 | 3 | k:Bacteria,p:Firmicutes,c:Clostridia,o:Oscillospirales,f:Oscillospiraceae,g:Colidextribacter,s:uncultured |
| OTU_1277 | 0 | 0 | 0.5 | k:Bacteria,p:Firmicutes,c:Clostridia,o:Clostridiales,f:Oscillospiraceae,g:Eubacterium,s:uncultured |
| OTU_1278 | 0 | 0 | 1 | k:Bacteria,p:Firmicutes,c:Clostridia,o:Oscillospirales,f:Oscillospiraceae,g:Oscillibacter,s:uncultured |
| OTU_1282 | 0 | 0 | 1.25 | k:Bacteria,p:Firmicutes,c:Clostridia,o:Lachnospirales,f:Lachnospiraceae,g:Hungatella,s:Hungatella effluvii |
| OTU_1285 | 1.5 | 0 | 0 | k:Bacteria,p:Proteobacteria,c:Alphaproteobacteria,o:Rhizobiales,f:Rhizobiaceae,g:Aureimonas,s:Aureimonas altamirensis |
| OTU_1286 | 0 | 0 | 0.5 | k:Bacteria,p:Firmicutes,c:Clostridia,o:Lachnospirales,f:Lachnospiraceae,g:PAC001090 |
| OTU_1287 | 0 | 0 | 0.5 | k:Bacteria,p:Firmicutes,c:Clostridia,o:Oscillospirales,f:Oscillospiraceae |
| OTU_1288 | 0 | 0 | 0.5 | k:Bacteria,p:Firmicutes,c:Clostridia,o:Lachnospirales,f:Lachnospiraceae,g:Enterocloster,s uncultured |
| OTU_1289 | 0 | 0 | 0.5 | k:Bacteria,p:Firmicutes,c:Clostridia,o:Lachnospirales,f:Lachnospiraceae,g:RAYR |
| OTU_129 | 0.5 | 0 | 41 | k:Bacteria,p:Firmicutes,c:Clostridia,o:Borkfalkiales,f:Borkfalkiaceae,g:PAC001141 |
| OTU_1293 | 0 | 0 | 0.75 | k:Bacteria,p:Firmicutes,c:Clostridia,o:Clostridiales,f:Oscillospiraceae,g:Ruminococcus,s:uncultured |
| OTU_1295 | 0 | 0 | 1 | k:Bacteria,p:Firmicutes,c:Clostridia,o:Lachnospirales,f:Lachnospiraceae,g:RAYR |
| OTU_1296 | 0 | 0 | 32.5 | k:Bacteria,p:Firmicutes,c:Clostridia,o:Clostridiales,f:Oscillospiraceae,g:PAC000661 |
| OTU_1297 | 0 | 0.5 | 0 | k:Bacteria,p:Proteobacteria,c:Gammaproteobacteria,o:Burkholderiales,f:Comamonadaceae,g:Aquabacterium,s:uncultured |
| OTU_1305 | 0 | 0 | 0.5 | k:Bacteria,p:Firmicutes,c:Bacilli,o:Lactobacillales,f:Lactobacillaceae,g:Lactobacillus,s:Lactobacillus helveticus |
| OTU_1307 | 0 | 1.25 | 2 | k:Bacteria,p:Bacteroidota,c:Bacteroidia,o:Bacteroidales,f:Muribaculaceae,g:PAC001472 |
| OTU_1320 | 0 | 0.75 | 0 | k:Bacteria,p:Firmicutes,c:Clostridia,o:Clostridiales,f:Oscillospiraceae,g:PAC002155 |
| OTU_132 | 0 | 60.75 | 119.25 | k:Bacteria,p:Firmicutes,c:Clostridia,o:Lachnospirales,f:Lachnospiraceae,g:Lachnospiraceae NK4A136 group,s:uncultured |
| OTU_1326 | 0 | 1.25 | 0 | k:Bacteria,p:Firmicutes,c:Bacilli,o:Bacillales,f:Bacillaceae,g:Pseudoneobacillus,s:Pseudoneobacillus rhizosphaerae |
| OTU_133 | 3 | 6.5 | 59.25 | k:Bacteria,p:Proteobacteria,c:Gammaproteobacteria,o:Burkholderiales,f:Oxalobacteraceae,g:Herbaspirillum,s:Herbaspirillum huttiense |
| OTU_134 | 0 | 55.75 | 52.5 | k:Bacteria,p:Firmicutes,c:Clostridia,o:Oscillospirales,f:Oscillospiraceae,g:uncultured |
| OTU_1340 | 0 | 0.5 | 0 | k:Bacteria,p:Firmicutes,c:Clostridia,o:Clostridiales,f:Oscillospiraceae,g:PAC000661 |
| OTU_1346 | 0 | 0.5 | 0 | k:Bacteria,p:Actinobacteriota,c:Actinobacteria,o:Micrococcales,f:Micrococcaceae,g:Rothia,s:Rothia nasimurium |
| OTU_1348 | 0 | 0 | 0.5 | k:Bacteria,p:Firmicutes,c:Clostridia,o:Lachnospirales,f:Lachnospiraceae,g:Acetitomaculum,s:Acetitomaculum ruminis |
| OTU_1351 | 0 | 0.75 | 1.5 | k:Bacteria,p:Bacteroidota,c:Bacteroidia,o:Bacteroidales,f:Muribaculaceae,g:PAC002448 |
| OTU_1352 | 0 | 0 | 3.25 | k:Bacteria,p:Firmicutes,c:Clostridia,o:Lachnospirales,f:Lachnospiraceae,g:KE159810 |
| OTU_1355 | 0 | 0 | 3.25 | k:Bacteria,p:Firmicutes,c:Clostridia,o:Oscillospirales,f:Oscillospiraceae,g:uncultured |
| OTU_1357 | 0 | 0 | 0.75 | k:Bacteria,p:Firmicutes,c:Clostridia,o:Borkfalkiales,f:Borkfalkiaceae,g:PAC001371 |
| OTU_1360 | 0 | 0 | 0.75 | k:Bacteria,p:Bacteroidetes,c:Bacteroidia,o:Bacteroidales,f:Muribaculaceae,g:PAC001512,s:uncultured |
| OTU_136 | 0 | 0 | 39.75 | k:Bacteria,p:Firmicutes,c:Clostridia,o:Lachnospirales,f:Lachnospiraceae,g:PAC001408 |
| OTU_1363 | 0 | 0.75 | 8.25 | k:Bacteria,p:Firmicutes,c:Clostridia,o:Oscillospirales,f:Oscillospiraceae,g:Colidextribacter,s:uncultured |
| OTU_1364 | 0 | 0.75 | 0 | k:Bacteria,p:Proteobacteria,c:Gammaproteobacteria,o:Xanthomonadales,f:Xanthomonadaceae,g:Pseudoxanthomonas,s:Pseudoxanthomonas mexicana |
| OTU_1366 | 0 | 1.5 | 3.5 | k:Bacteria,p:Firmicutes,c:Bacilli,o:Lactobacillales,f:Lactobacillaceae,g:Lactobacillus,s:Limosilactobacillus ingluviei |
| OTU_1375 | 0 | 0 | 0.5 | k:Bacteria,p:Firmicutes,c:Clostridia,o:Clostridiales,f:Oscillospiraceae,g:uncultured |
| OTU_1377 | 0 | 0 | 0.75 | k:Bacteria,p:Firmicutes,c:Clostridia,o:Lachnospirales,f:Lachnospiraceae,g:uncultured |
| OTU_1383 | 0 | 0.25 | 0.5 | k:Bacteria,p:Actinobacteriota,c:Acidimicrobiia,o:IMCC26256 |
| OTU_1384 | 0 | 0 | 1 | k:Bacteria,p:Bacteroidota,c:Bacteroidia,o:Bacteroidales,f:Bacteroidaceae,g:Bacteroides,s:uncultured |
| OTU_1388 | 0 | 0.5 | 0 | k:Bacteria,p:Proteobacteria,c:Alphaproteobacteria,o:Rhodospirillales,f:uncultured |
| OTU_1397 | 0 | 0.5 | 0.5 | k:Bacteria,p:Firmicutes,c:Clostridia,o:Oscillospirales,f:Oscillospiraceae |
| OTU_1398 | 0 | 0 | 1.25 | k:Bacteria,p:Firmicutes,c:Clostridia,o:Clostridiales,f:Lachnospiraceae,g:uncultured |
| OTU_139 | 4.5 | 15 | 30.75 | k:Bacteria,p:Firmicutes,c:Bacilli,o:Lactobacillales,f:Streptococcaceae,g:Streptococcus,s:Streptococcus azizii |
| OTU_1401 | 0 | 0 | 0.5 | k:Bacteria,p:Firmicutes,c:Clostridia,o:Lachnospirales,f:Lachnospiraceae,g:KE159810 |
| OTU_1403 | 0 | 0 | 0.5 | k:Bacteria,p:Proteobacteria,c:Gammaproteobacteria,o:Burkholderiales,f:Burkholderiaceae,g:Burkholderia,s:Burkholderia singularis |
| OTU_1409 | 0 | 1 | 0 | k:Bacteria,p:Firmicutes,c:Clostridia,o:Christensenellales,f:Christensenellaceae,g:uncultured |
| OTU_1410 | 0 | 0 | 0.5 | k:Bacteria,p:Firmicutes,c:Clostridia,o:Oscillospirales,f:Ruminococcaceae,g:uncultured |
| OTU_141 | 13.5 | 5 | 43 | k:Bacteria,p:Proteobacteria,c:Alphaproteobacteria,o:Caulobacterales,f:Caulobacteraceae,g:Brevundimonas |
| OTU_1413 | 0 | 1 | 0.75 | k:Bacteria,p:Verrucomicrobiota,c:Verrucomicrobiae,o:Chthoniobacterales,f:Chthoniobacteraceae,g:Candidatus Udaeobacter |
| OTU_1419 | 0 | 1 | 0 | k:Bacteria,p:Acidobacteriota,c:Subgroup 5 |
| OTU_142 | 0.5 | 12.75 | 101 | k:Bacteria,p:Firmicutes,c:Clostridia,o:Borkfalkiales,f:Borkfalkiaceae,g:PAC001440,s:uncultured |
| OTU_1425 | 0 | 1 | 0 | k:Bacteria,p:Bacteroidota,c:Bacteroidia,o:Sphingobacteriales,f:Sphingobacteriaceae,g:Sphingobacterium |
| OTU_1427 | 0 | 0.5 | 0 | k:Bacteria,p:Firmicutes,c:Clostridia,o:Oscillospirales,f:[Eubacterium] coprostanoligenes |
| OTU_1436 | 0 | 0.5 | 0 | k:Bacteria,p:Proteobacteria,c:Gammaproteobacteria,o:Enterobacterales,f:Enterobacteriaceae,g:Escherichia-Shigella,s:E. coli |
| OTU_1438 | 0 | 1.25 | 0 | k:Bacteria,p:Firmicutes,c:Clostridia,o:Lachnospirales,f:Lachnospiraceae,g:Lachnospiraceae UCG-001,s:uncultured |
| OTU_144 | 36.5 | 159.25 | 29.75 | k:Bacteria,p:Firmicutes,c:Bacilli,o:Lactobacillales,f:Streptococcaceae,g:Streptococcus,s:uncultured |
| OTU_146 | 0 | 51.75 | 11.75 | k:Bacteria,p:Firmicutes,c:Bacilli,o:Staphylococcales,f:Staphylococcaceae,g:Staphylococcus,s:Staphylococcus equorum |
| OTU_1462 | 0 | 0.25 | 1.25 | k:Bacteria,p:Firmicutes,c:Clostridia,o:Monoglobales,f:Monoglobaceae,g:Monoglobus,s:uncultured |
| OTU_147 | 0 | 0 | 24 | k:Bacteria,p:Firmicutes,c:Bacilli,o:Acholeplasmatales,f:Acholeplasmataceae,g:Anaeroplasma |
| OTU_1476 | 0 | 0.75 | 16.75 | k:Bacteria,p:Firmicutes,c:Clostridia,o:Borkfalkiales,f:Borkfalkiaceae,g:PAC001360,s:uncultured |
| OTU_1487 | 2.5 | 0.25 | 0 | k:Bacteria,p:Bacteroidota,c:Bacteroidia,o:Chitinophagales,f:Chitinophagaceae |
| OTU_1489 | 0 | 1 | 0 | k:Bacteria,p:Actinobacteriota,c:Actinobacteria,o:Actinomycetales,f:Actinomycetaceae,g:Actinomyces,s:Actinomyces gerencseriae |
| OTU_149 | 8 | 9.25 | 24.5 | k:Bacteria,p:Proteobacteria,c:Gammaproteobacteria,o:Burkholderiales,f:Burkholderiaceae,g:Ralstonia,s:Ralstonia pickettii |
| OTU_1494 | 0 | 0.5 | 0 | k:Bacteria,p:Firmicutes,c:Clostridia,o:Lachnospirales,f:Lachnospiraceae,g:Fusimonas,s:uncultured |
| OTU_1495 | 0 | 2.5 | 0 | k:Bacteria,p:Firmicutes,c:Clostridia,o:Lachnospirales,f:Lachnospiraceae |
| OTU_150 | 0 | 6 | 14 | k:Bacteria,p:Proteobacteria,c:Gammaproteobacteria,o:Xanthomonadales,f:Xanthomonadaceae,g:Stenotrophomonas |
| OTU_1507 | 0 | 0 | 0.75 | k:Bacteria,p:Firmicutes,c:Clostridia,o:Oscillospirales,f:Oscillospiraceae,g:Merdimmobilis,s:uncultured |
| OTU_1509 | 0 | 0 | 0.5 | k:Bacteria,p:Firmicutes,c:Clostridia,o:Lachnospirales,f:Lachnospiraceae,g:PAC001165 |
| OTU_151 | 0 | 0 | 83.5 | k:Bacteria,p:Firmicutes,c:Clostridia,o:Borkfalkiales,f:Borkfalkiaceae,g:PAC001360 |
| OTU_1512 | 0 | 0 | 0.75 | k:Bacteria,p:Firmicutes,c:Clostridia,o:Borkfalkiales,f:Borkfalkiaceae,g:uncultured |
| OTU_1513 | 0 | 0 | 0.75 | k:Bacteria,p:Firmicutes,c:Clostridia,o:Oscillospirales,f:Oscillospiraceae,g:V9D2013 group |
| OTU_1520 | 0 | 0 | 1 | k:Bacteria,p:Firmicutes,c:Bacilli,o:uncultured |
| OTU_152 | 5 | 10.75 | 37 | k:Bacteria,p:Firmicutes,c:Clostridia,o:Clostridiales,f:Clostridiaceae,g:Clostridium,s:Clostridium celatum |
| OTU_15 | 2.5 | 7.75 | 1017 | k:Bacteria,p:Firmicutes,c:Clostridia,o:Oscillospirales,f:Ruminococcaceae,g:Ruminococcus,s:uncultured |
| OTU_1528 | 0 | 0 | 2 | k:Bacteria,p:Firmicutes,c:Clostridia,o:Oscillospirales,f:Oscillospiraceae,g:uncultured |
| OTU_1532 | 0 | 77.75 | 3.25 | k:Bacteria,p:Firmicutes,c:Clostridia,o:Lachnospirales,f:Lachnospiraceae |
| OTU_1534 | 0 | 0 | 1 | k:Bacteria,p:Verrucomicrobiota,c:Verrucomicrobiae,o:Pedosphaerales,f:Pedosphaeraceae,g:uncultured |
| OTU_1535 | 0 | 1 | 7.5 | k:Bacteria,p:Firmicutes,c:Clostridia,o:Lachnospirales,f:Lachnospiraceae,g:Lachnospiraceae NK4A136,s:uncultured |
| OTU_154 | 0 | 0 | 24.25 | k:Bacteria,p:Proteobacteria,c:Alphaproteobacteria,o:Paracaedibacterales,f:Paracaedibacteraceae,g:uncultured |
| OTU_1540 | 0 | 0.75 | 0 | k:Bacteria,p:Bacteroidota,c:Bacteroidia,o:Sphingobacteriales,f:Sphingobacteriaceae,g:Sphingobacterium,s:uncultured |
| OTU_1543 | 0 | 3.5 | 0 | k:Bacteria,p:Firmicutes,c:Clostridia,o:Lachnospirales,f:Lachnospiraceae,g:Blautia,s:uncultured |
| OTU_1545 | 0 | 1.75 | 0 | k:Bacteria,p:Proteobacteria,c:Gammaproteobacteria,o:Burkholderiales,f:Comamonadaceae,g:Pseudacidovorax |
| OTU_1547 | 0 | 1.25 | 0 | k:Bacteria,p:Firmicutes,c:Clostridia,o:Lachnospirales,f:Lachnospiraceae,g:KE159571 |
| OTU_1549 | 0 | 0.5 | 0 | k:Bacteria,p:Firmicutes,c:Clostridia,o:Lachnospirales,f:Lachnospiraceae,g:QUHQ |
| OTU_1551 | 0 | 1 | 0 | k:Bacteria,p:Verrucomicrobiota,c:Verrucomicrobiae,o:Chthoniobacterales,f:Chthoniobacteraceae,g:Candidatus Udaeobacter |
| OTU_1553 | 0 | 0 | 3 | k:Bacteria,p:Firmicutes,c:Bacilli,o:Izemoplasmatales,f:PAC001265 |
| OTU_155 | 2 | 54.25 | 9.25 | k:Bacteria,p:Firmicutes,c:Bacilli,o:Staphylococcales,f:Staphylococcaceae,g:Staphylococcus,s:Staphylococcus sciuri |
| OTU_1555 | 0 | 0.5 | 0 | k:Bacteria,p:Firmicutes,c:Bacilli,o:Lactobacillales,f:Lactobacillaceae,g:Lactobacillus,s:Lactobacillus colini |
| OTU_1556 | 0 | 1.25 | 0 | k:Bacteria,p:Firmicutes,c:Clostridia,o:Oscillospirales,f:Oscillospiraceae,g:uncultured |
| OTU_1558 | 0 | 0.5 | 0 | k:Bacteria,p:Firmicutes,c:Clostridia,o:Lachnospirales,f:Lachnospiraceae,g:PAC001116 |
| OTU_156 | 0 | 1.25 | 68.75 | k:Bacteria,p:Firmicutes,c:Clostridia,o:Borkfalkiales,f:Borkfalkiaceae,g:PAC001141 |
| OTU_1562 | 0 | 0 | 0.5 | k:Bacteria,p:Firmicutes,c:Clostridia,o:Lachnospirales,f:Lachnospiraceae,g:Porcincola,s:uncultured |
| OTU_1563 | 0 | 0.5 | 0 | k:Bacteria,p:Firmicutes,c:Clostridia,o:Lachnospirales,f:Lachnospiraceae,g:KE159600 |
| OTU_1565 | 0 | 0 | 0.5 | k:Bacteria,p:Firmicutes,c:Clostridia,o:Oscillospirales,f:UCG-010 |
| OTU_1569 | 0 | 1.25 | 0 | k:Bacteria,p:Actinobacteriota,c:Actinobacteria,o:Micrococcales,f:Intrasporangiaceae,g:Ornithinimicrobium |
| OTU_157 | 0 | 32 | 0 | k:Bacteria,p:Actinobacteriota,c:Coriobacteriia,o:Coriobacteriales,f:Eggerthellaceae |
| OTU_158 | 0 | 18.5 | 38.5 | k:Bacteria,p:Bacteroidota,c:Bacteroidia,o:Bacteroidales,f:Muribaculaceae,g:Muribaculum,s:Muribaculum gordoncarteri |
| OTU_159 | 0 | 0 | 53.5 | k:Bacteria,p:Spirochaetota,c:Spirochaetia,o:Spirochaetales,f:Spirochaetaceae,g:Treponema,s:Treponema succinifaciens |
| OTU_1592 | 0 | 0.75 | 0 | k:Bacteria,p:Proteobacteria,c:Alphaproteobacteria,o:Reyranellales,f:Reyranellaceae,g:Reyranella |
| OTU_1593 | 0 | 0.75 | 0.75 | k:Bacteria,p:Firmicutes,c:Clostridia,o:Oscillospirales,f:Butyricicoccaceae,g:Butyricicoccus,s:uncultured |
| OTU_1594 | 0 | 1.25 | 0 | k:Bacteria,p:Bacteroidota,c:Bacteroidia,o:Bacteroidales,f:Muribaculaceae,g:RIAY |
| OTU_1599 | 0 | 0.75 | 0 | k:Bacteria,p:Actinobacteriota,c:Actinobacteria,o:Corynebacteriales,f:Nocardiaceae,g:Rhodococcus,s:Rhodococcus jostii |
| OTU_1604 | 0 | 3 | 0 | k:Bacteria,p:Bacteroidota,c:Bacteroidia,o:Bacteroidales,f:Bacteroidaceae,g:Bacteroides,s:uncultured |
| OTU_1606 | 0 | 5 | 1.25 | k:Bacteria,p:Firmicutes,c:Clostridia,o:Lachnospirales,f:Lachnospiraceae,g:RAYR |
| OTU_1608 | 1 | 0 | 0.5 | k:Bacteria,p:Firmicutes,c:Bacilli,o:Lactobacillales,f:Lactobacillaceae,g:Limosilactobacillus,s:Limosilactobacillus coleohominis |
| OTU_1611 | 0 | 0 | 1 | k:Bacteria,p:Bacteroidota,c:Bacteroidia,o:Bacteroidales,f:Muribaculaceae,g:PAC000198 |
| OTU_1613 | 0 | 0 | 1.75 | k:Bacteria,p:Firmicutes,c:Clostridia,o:Lachnospirales,f:Lachnospiraceae,g:uncultured |
| OTU_16 | 2011.5 | 2.25 | 2 | k:Bacteria,p:Firmicutes,c:Bacilli,o:Mycoplasmatales,f:Mycoplasmataceae,g:Mycoplasma,s:Mycoplasma sualvi |
| OTU_1615 | 0 | 3 | 1.75 | k:Bacteria,p:Bacteroidota,c:Bacteroidia,o:Bacteroidales,f:Rikenellaceae,g:Alistipes,s:uncultured |
| OTU_1616 | 0 | 5 | 3.5 | k:Bacteria,p:Firmicutes,c:Clostridia,o:Oscillospirales,f:Oscillospiraceae |
| OTU_1617 | 0 | 0 | 0.75 | k:Bacteria,p:Firmicutes,c:Clostridia,o:Lachnospirales,f:Lachnospiraceae,g:RAYR |
| OTU_1620 | 0 | 0 | 2.75 | k:Bacteria,p:Firmicutes,c:Clostridia,o:Borkfalkiales,f:PAC001219,g:PAC001219 |
| OTU_1621 | 0 | 0 | 1 | k:Bacteria,p:Firmicutes,c:Clostridia,o:Borkfalkiales,f:PAC001219,g:AB243283 |
| OTU_1623 | 0 | 0 | 0.5 | k:Bacteria,p:Firmicutes,c:Clostridia,o:Clostridiales,f:Lachnospiraceae,g:Sellimonas,s:Sellimonas caecigallum |
| OTU_1625 | 0 | 0 | 0.5 | k:Bacteria,p:Bacteroidota,c:Bacteroidia,o:Bacteroidales,f:Muribaculaceae,g:PAC001472 |
| OTU_1626 | 0 | 0 | 3.25 | k:Bacteria,p:Bacteroidota,c:Bacteroidia,o:Bacteroidales,f:Muribaculaceae,g:Duncaniella,s:Duncaniella muris |
| OTU_1627 | 0 | 0 | 0.75 | k:Bacteria,p:Actinobacteriota,c:Coriobacteriia,o:Coriobacteriales,f:Eggerthellaceae |
| OTU_1628 | 0 | 0 | 0.75 | k:Bacteria,p:Firmicutes,c:Clostridia,o:Clostridiales,f:Oscillospiraceae,g:Zongyangia,s:uncultured |
| OTU_1629 | 0 | 0 | 0.75 | k:Bacteria,p:Firmicutes,c:Bacilli,o:Lactobacillales,f:Lactobacillaceae,g:Ligilactobacillus,s:Ligilactobacillus faecis |
| OTU_1630 | 0 | 0 | 0.5 | k:Bacteria,p:Firmicutes,c:Clostridia,o:Oscillospirales,f:Ruminococcaceae,g:uncultured |
| OTU_163 | 0.5 | 1.75 | 120.5 | k:Bacteria,p:Firmicutes,c:Clostridia,o:Lachnospirales,f:Lachnospiraceae,g:Lachnospiraceae NK4A136 group,s:uncultured |
| OTU_1633 | 0 | 0 | 0.5 | k:Bacteria,p:Bacteroidota,c:Bacteroidia,o:Bacteroidales,f:Muribaculaceae,g:PAC001472 |
| OTU_1634 | 0 | 0 | 0.5 | k:Bacteria,p:Firmicutes,c:Clostridia,o:Borkfalkiales,f:PAC001219,g:PAC001219 |
| OTU_1640 | 0 | 4 | 13.25 | k:Bacteria,p:Firmicutes,c:Clostridia,o:Lachnospirales,f:Lachnospiraceae,g:Roseburia,s:uncultured |
| OTU_164 | 0 | 39.5 | 0 | k:Bacteria,p:Bacteroidota,c:Bacteroidia,o:Bacteroidales,f:Rikenellaceae,g:Alistipes,s:uncultured |
| OTU_1642 | 0 | 0 | 1 | k:Bacteria,p:Firmicutes,c:Clostridia,o:Borkfalkiales,f:PAC001219,g:GQ448104 |
| OTU_1647 | 0 | 4.75 | 1 | k:Bacteria,p:Bacteroidota,c:Bacteroidia,o:Bacteroidales,f:Bacteroidaceae,g:Bacteroides,s:Bacteroides ovatus |
| OTU_1652 | 0 | 0 | 4 | k:Bacteria,p:Proteobacteria,c:Alphaproteobacteria,o:Rhizobiales,f:Devosiaceae,g:Devosia |
| OTU_1656 | 0 | 0 | 5.75 | k:Bacteria,p:Firmicutes,c:Clostridia,o:Lachnospirales,f:Lachnospiraceae,g:KE159810 |
| OTU_1659 | 2.5 | 0.25 | 4.5 | k:Bacteria,p:Firmicutes,c:Clostridia,o:Lachnospirales,f:Lachnospiraceae,g:RAYR |
| OTU_1660 | 0 | 0 | 2.75 | k:Bacteria,p:Firmicutes,c:Clostridia,f:Hungateiclostridiaceae,g:Ruminiclostridium,s:uncultured |
| OTU_166 | 0 | 0 | 33.5 | k:Bacteria,p:Bacteroidota,c:Bacteroidia,o:Bacteroidales,f:Bacteroidaceae,g:Bacteroides,s:uncultured |
| OTU_1661 | 0 | 0 | 0.75 | k:Bacteria,p:Firmicutes,c:Bacilli,o:RF39 |
| OTU_1662 | 0 | 0 | 7.25 | k:Bacteria,p:Firmicutes,c:Clostridia,o:Borkfalkiales,f:Borkfalkiaceae,g:PAC001360 |
| OTU_1666 | 0 | 0 | 0.5 | k:Bacteria,p:Firmicutes,c:Bacilli,o:Lactobacillales,f:Lactobacillaceae,g:Lactobacillus,s:Lactobacillus johnsonii |
| OTU_1668 | 0 | 0 | 0.75 | k:Bacteria,p:Firmicutes,c:Bacilli,o:RF39 |
| OTU_1669 | 0 | 0 | 4 | k:Bacteria,p:Firmicutes,c:Clostridia,o:Oscillospirales,f:Ruminococcaceae,g:uncultured |
| OTU_167 | 0 | 19.75 | 0.25 | k:Bacteria,p:Firmicutes,c:Clostridia,o:Lachnospirales,f:Lachnospiraceae,g:Tuzzerella,s:uncultured |
| OTU_1671 | 0 | 0 | 1 | k:Bacteria,p:Firmicutes,c:Clostridia,o:Lachnospirales,f:Lachnospiraceae,g:Acetatifactor,s:uncultured |
| OTU_1674 | 0 | 0 | 0.5 | k:Bacteria,p:Bacteroidota,c:Bacteroidia,o:Bacteroidales,f:Rikenellaceae,g:Alistipes,s:Alistipes dk3620 |
| OTU_1678 | 0 | 0 | 1 | k:Bacteria,p:Bacteroidetes,c:Bacteroidia,o:Bacteroidales,f:Bacteroidaceae,g:Phocaeicola,s:Phocaeicola sartorii |
| OTU_168 | 0.5 | 2 | 30 | k:Bacteria,p:Firmicutes,c:Clostridia,o:Lachnospirales,f:Lachnospiraceae,g:Bacteroides,s:Bacteroides pectinophilus |
| OTU_1681 | 0 | 0 | 1 | k:Bacteria,p:Firmicutes,c:Bacilli,o:Erysipelotrichales,f:Erysipelatoclostridiaceae,g:Erysipelotrichaceae UCG,s:Lactobacillus |
| OTU_1682 | 0 | 0 | 1 | k:Bacteria,p:Firmicutes,c:Clostridia,o:Borkfalkiales,f:Borkfalkiaceae,g:PAC001360 |
| OTU_1683 | 0 | 0 | 1.75 | k:Bacteria,p:Firmicutes,c:Clostridia,o:Borkfalkiales,f:Borkfalkiaceae,g:uncultured |
| OTU_1685 | 2 | 0.25 | 275 | k:Bacteria,p:Campilobacterota,c:Campylobacteria,o:Campylobacterales,f:Helicobacteraceae,g:Helicobacter,s:Helicobacter rodentium |
| OTU_1687 | 0 | 0 | 1.5 | k:Bacteria,p:Bacteroidota,c:Bacteroidia,o:Bacteroidales,f:Muribaculaceae,g:PAC001112 |
| OTU_1688 | 0 | 0 | 0.5 | k:Bacteria,p:Firmicutes,c:Clostridia,o:Borkfalkiales,f:Borkfalkiaceae,g:uncultured |
| OTU_1689 | 0.5 | 0 | 0.5 | k:Bacteria,p:Firmicutes,c:Bacilli,o:Lactobacillales,f:Enterococcaceae,g:Enterococcus,s:Enterococcus diestrammenae |
| OTU_1695 | 0 | 0 | 2.5 | k:Bacteria,p:Firmicutes,c:Clostridia,o:Oscillospirales,f:Oscillospiraceae |
| OTU_1696 | 0 | 0.25 | 5.25 | k:Bacteria,p:Firmicutes,c:Clostridia,o:Clostridia UCG-014 |
| OTU_1697 | 0 | 1.75 | 3.25 | k:Bacteria,p:Bacteroidota,c:Bacteroidia,o:Bacteroidales,f:Muribaculaceae,g:RIAY |
| OTU_1699 | 0 | 0 | 1.5 | k:Bacteria,p:Proteobacteria,c:Gammaproteobacteria,o:Burkholderiales,f:Ralstonia,g:Ralstonia,s:Ralstonia insidiosa |
| OTU_1701 | 0 | 0 | 0.5 | k:Bacteria,p:Firmicutes,c:Bacilli,o:Lactobacillales,f:Lactobacillaceae,g:Lactobacillus,s:Lactobacillus johnsonii |
| OTU_1706 | 0 | 0.25 | 0.5 | k:Bacteria,p:Proteobacteria,c:Alphaproteobacteria,o:Rhizobiales,f:Beijerinckiaceae |
| OTU_171 | 0 | 21.5 | 30 | k:Bacteria,p:Firmicutes,c:Clostridia,o:Oscillospirales |
| OTU_1712 | 0 | 0.75 | 0.25 | k:Bacteria,p:Firmicutes,c:Clostridia,o:Oscillospirales,f:Butyricicoccaceae,g:Butyricicoccus,s:uncultured |
| OTU_1715 | 186 | 81.25 | 78.5 | k:Bacteria,p:Firmicutes,c:Bacilli,o:Lactobacillales,f:Lactobacillaceae,g:Lactobacillus,s:Lactobacillus intestinalis |
| OTU_1717 | 1 | 0 | 0 | k:Bacteria,p:Firmicutes,c:Clostridia,o:Borkfalkiales,f:Borkfalkiaceae,g:PAC001371 |
| OTU_1719 | 0 | 2 | 0 | k:Bacteria,p:Bacteroidetes,c:Bacteroidia,o:Bacteroidales,f:Muribaculaceae,g:PAC001765 |
| OTU_172 | 0 | 11.5 | 30.75 | k:Bacteria,p:Firmicutes,c:Clostridia,o:Lachnospirales,f:Lachnospiraceae,g:uncultured |
| OTU_1721 | 0 | 2 | 0 | k:Bacteria,p:Firmicutes,c:Bacilli,o:Erysipelotrichales,f:Erysipelotrichaceae |
| OTU_1725 | 0 | 0 | 0.5 | k:Bacteria,p:Firmicutes,c:Clostridia,o:Clostridiales,f:Oscillospiraceae,g:Agathobaculum,s:uncultured |
| OTU_1727 | 0 | 2.5 | 0.25 | k:Bacteria,p:Firmicutes,c:Clostridia,o:Oscillospirales,f:Ruminococcaceae,g:uncultured |
| OTU_1728 | 0 | 0 | 0.75 | k:Bacteria,p:Firmicutes,c:Clostridia,o:Oscillospirales,f:Butyricicoccaceae,g:Butyricicoccus,s:uncultured |
| OTU_17 | 1600.5 | 6 | 1.75 | k:Bacteria,p:Campilobacterota,c:Campylobacteria,o:Campylobacterales,f:Helicobacteraceae,g:Helicobacter,s:Helicobacter apodemus |
| OTU_173 | 2.5 | 3.75 | 19.5 | k:Bacteria,p:Proteobacteria,c:Gammaproteobacteria,o:Burkholderiales,f:Comamonadaceae,g:Delftia,s:Delftia acidovorans |
| OTU_1733 | 0 | 4 | 0.25 | k:Bacteria,p:Firmicutes,c:Clostridia,o:Lachnospirales,f:Lachnospiraceae,g:uncultured |
| OTU_1737 | 0 | 0 | 0.5 | k:Bacteria,p:Firmicutes,c:Clostridia,o:Oscillospirales,f:Ruminococcaceae |
| OTU_1738 | 0 | 0 | 8.25 | k:Bacteria,p:Firmicutes,c:Clostridia,o:Borkfalkiales,f:PAC001219,g:PAC001219 |
| OTU_1740 | 0 | 16.25 | 23.75 | k:Bacteria,p:Firmicutes,c:Clostridia,o:Lachnospirales,f:Lachnospiraceae,g:Lachnospiraceae NK4A136,s:uncultured |
| OTU_1741 | 0 | 0 | 7.5 | k:Bacteria,p:Firmicutes,c:Clostridia,o:Oscillospirales,f:Oscillospiraceae |
| OTU_174 | 0.5 | 23 | 172.5 | k:Bacteria,p:Firmicutes,c:Clostridia,o:Oscillospirales,f:Oscillospiraceae,g:uncultured |
| OTU_1745 | 0 | 0 | 3 | k:Bacteria,p:Bacteroidetes,c:Bacteroidia,o:Bacteroidales,f:Muribaculaceae,g:PAC000198 |
| OTU_1747 | 0 | 0.25 | 0.5 | k:Bacteria,p:Proteobacteria,c:Gammaproteobacteria,o:Burkholderiales,f:Ralstonia,g:Cupriavidus,s:Cupriavidus gilardii |
| OTU_1748 | 0 | 0.25 | 0.25 | k:Bacteria,p:Firmicutes,c:Clostridia,o:Lachnospirales,f:Lachnospiraceae,g:PAC001516 |
| OTU_1749 | 0 | 8.25 | 0 | k:Bacteria,p:Firmicutes,c:Clostridia,o:Clostridiales,f:Lachnospiraceae,g:Dorea,s:Dorea formicigenerans |
| OTU_175 | 0 | 1.5 | 69.5 | k:Bacteria,p:Firmicutes,c:Clostridia,o:Borkfalkiales,f:Borkfalkiaceae,g:PAC001360 |
| OTU_1753 | 0 | 0 | 10 | k:Bacteria,p:Bacteroidota,c:Bacteroidia,o:Bacteroidales,f:Bacteroidaceae,g:Bacteroides,s:uncultured |
| OTU_1756 | 0 | 0 | 1 | k:Bacteria,p:Firmicutes,c:Clostridia,o:Clostridiales,f:Lachnospiraceae,g:AB185516,s:uncultured |
| OTU_176 | 0 | 4.5 | 40.75 | k:Bacteria,p:Firmicutes,c:Clostridia,o:Monoglobales,f:Monoglobaceae,g:Monoglobus,s:uncultured |
| OTU_1765 | 0 | 0 | 0.5 | k:Bacteria,p:Firmicutes,c:Clostridia,o:Clostridiales,f:Oscillospiraceae,g:PAC000661 |
| OTU_1767 | 0 | 0 | 5 | k:Bacteria,p:Firmicutes,c:Clostridia,o:Lachnospirales,f:Lachnospiraceae,g:Lachnospiraceae NK4A136 group,s:uncultured |
| OTU_1768 | 0 | 0 | 0.5 | k:Bacteria,p:Firmicutes,c:Clostridia,o:Clostridiales,f:Oscillospiraceae,g:Pseudoflavonifractor,s:uncultured |
| OTU_1769 | 0 | 0 | 1.25 | k:Bacteria,p:Firmicutes,c:Clostridia,o:Clostridiales,f:Lachnospiraceae,g:uncultured |
| OTU_1770 | 0 | 0 | 0.75 | k:Bacteria,p:Bacteroidetes,c:Bacteroidia,o:Bacteroidales,f:Muribaculaceae,g:PAC000198 |
| OTU_1771 | 4.5 | 1 | 34.25 | k:Bacteria,p:Proteobacteria,c:Alphaproteobacteria,o:Rhizobiales,f:Rhizobiaceae,g:Mesorhizobium,s:Mesorhizobium australicum |
| OTU_1774 | 0 | 0 | 3.5 | k:Bacteria,p:Firmicutes,c:Clostridia,o:Clostridiales,f:Oscillospiraceae,g:PAC000661 |
| OTU_1777 | 0 | 0 | 1.25 | k:Bacteria,p:Firmicutes,c:Clostridia,o:Clostridiales,f:Lachnospiraceae,g:Enterocloster,s:Lachnoclostridium pacaense |
| OTU_1780 | 0 | 0 | 3 | k:Bacteria,p:Firmicutes,c:Clostridia,o:Oscillospirales,f:Ruminococcaceae,g:Incertae sedis,s:uncultured |
| OTU_1782 | 0 | 1.75 | 0 | k:Bacteria,p:Bacteroidota,c:Bacteroidia,o:Bacteroidales,f:Muribaculaceae |
| OTU_1783 | 0 | 0 | 1.25 | k:Bacteria,p:Actinobacteriota,c:Coriobacteriia,o:Coriobacteriales,f:Eggerthellaceae,g:Enterorhabdus,s:uncultured |
| OTU_1789 | 0 | 0 | 0.75 | k:Bacteria,p:Firmicutes,c:Clostridia,o:Clostridiales,f:Lachnospiraceae,g:Velocimicrobium,s:uncultured |
| OTU_1790 | 0 | 0 | 2 | k:Bacteria,p:Bacteroidetes,c:Bacteroidia,o:Bacteroidales,f:Muribaculaceae,g:PAC001074 |
| OTU_179 | 0 | 26.75 | 47.75 | k:Bacteria,p:Bacteroidota,c:Bacteroidia,o:Bacteroidales,f:Muribaculaceae,g:PAC001112 |
| OTU_1794 | 0 | 1.75 | 0 | k:Bacteria,p:Firmicutes,c:Bacilli,o:Lactobacillales,f:Streptococcaceae,g:Streptococcus,s:Streptococcus ferus |
| OTU_1795 | 0 | 0 | 4.75 | k:Bacteria,p:Bacteroidota,c:Bacteroidia,o:Bacteroidales,f:Muribaculaceae |
| OTU_1796 | 0 | 3.5 | 0.25 | k:Bacteria,p:Desulfobacterota,c:Desulfovibrionia,o:Desulfovibrionales,f:Desulfovibrionaceae,g:Desulfovibrio,s:uncultured |
| OTU_1797 | 0 | 0 | 2.25 | k:Bacteria,p:Firmicutes,c:Clostridia,o:Lachnospirales,f:Lachnospiraceae,g:Blautia,s:uncultured |
| OTU_1799 | 0 | 0 | 1.5 | k:Bacteria,p:Firmicutes,c:Clostridia,o:Clostridiales,f:Lachnospiraceae,g:Schaedlerella,s:uncultured |
| OTU_1800 | 0 | 1.75 | 0 | k:Bacteria,p:Firmicutes,c:Clostridia,o:Lachnospirales,f:Lachnospiraceae,g:Frisingicoccus,s:Frisingicoccus caecimuris |
| OTU_180 | 0 | 18 | 1.25 | k:Bacteria,p:Bacteroidota,c:Bacteroidia,o:Bacteroidales,f:Bacteroidaceae,g:Bacteroides,s:Bacteroides caccae |
| OTU_1801 | 0 | 0.5 | 0 | k:Bacteria,p:Proteobacteria,c:Gammaproteobacteria,o:Pasteurellales,f:Pasteurellaceae,g:Haemophilus,s:Haemophilus parainfluenzae |
| OTU_1802 | 0 | 0 | 0.5 | k:Bacteria,p:Firmicutes,c:Clostridia,o:Clostridiales,f:Lachnospiraceae,g:Blautia,s:Blautia hydrogenotrophica |
| OTU_1805 | 0 | 0 | 3.75 | k:Bacteria,p:Bacteroidota,c:Bacteroidia,o:Bacteroidales,f:Muribaculaceae |
| OTU_1807 | 0 | 0 | 1.5 | k:Bacteria,p:Firmicutes,c:Clostridia,o:Lachnospirales,f:Lachnospiraceae,g:uncultured |
| OTU_181 | 0 | 35 | 10.5 | k:Bacteria,p:Firmicutes,c:Clostridia,o:Lachnospirales,f:Lachnospiraceae,g:Roseburia,s:uncultured |
| OTU_1811 | 0 | 0.5 | 0 | k:Bacteria,p:Cyanobacteria,c:Vampirivibrionia,o:Gastranaerophilales,f:FR888536 |
| OTU_1816 | 0 | 0 | 0.5 | k:Bacteria,p:Firmicutes,c:Clostridia,o:Borkfalkiales,f:Borkfalkiaceae,g:uncultured |
| OTU_1817 | 0 | 0 | 0.5 | k:Bacteria,p:Bacteroidetes,c:Bacteroidia,o:Bacteroidales,f:Bacteroidaceae,g:Bacteroides,s:uncultured |
| OTU_1818 | 0 | 1.5 | 8 | k:Bacteria,p:Firmicutes,c:Clostridia,o:Clostridiales,f:Lachnospiraceae,g:Enterocloster,s:uncultured |
| OTU_1821 | 0 | 0 | 0.75 | k:Bacteria,p:Firmicutes,c:Clostridia,o:Clostridiales,f:Lachnospiraceae,g:PAC002042 |
| OTU_1822 | 0 | 1.75 | 0 | k:Bacteria,p:Cyanobacteria,c:Vampirivibrionia,o:Gastranaerophilales,f:FR888536 |
| OTU_1826 | 0 | 0 | 0.75 | k:Bacteria,p:Firmicutes,c:Clostridia,o:Monoglobales,f:Monoglobaceae,g:Monoglobus,s:uncultured |
| OTU_1827 | 0 | 3 | 3 | k:Bacteria,p:Firmicutes,c:Clostridia,o:Clostridiales,f:Oscillospiraceae,g:PAC000661 |
| OTU_1828 | 0 | 0 | 1.5 | k:Bacteria,p:Desulfobacterota,c:Desulfovibrionia,o:Desulfovibrionales,f:Desulfovibrionaceae,g:uncultured |
| OTU_1829 | 0 | 3.25 | 0 | k:Bacteria,p:Firmicutes,c:Negativicutes,o:Veillonellales-Selenomonadales,f:Veillonellaceae,g:Dialister,s:uncultured |
| OTU_1830 | 0 | 0 | 0.5 | k:Bacteria,p:Firmicutes,c:Clostridia,o:Clostridiales,f:Lachnospiraceae,g:RAYR,s:uncultured |
| OTU_1834 | 0 | 0 | 1 | k:Bacteria,p:Firmicutes,c:Clostridia,o:Lachnospirales,f:Lachnospiraceae,g:uncultured |
| OTU_1835 | 0 | 1.5 | 0 | k:Bacteria,p:Desulfobacterota,c:Desulfovibrionia,o:Desulfovibrionales,f:Desulfovibrionaceae,g:Desulfovibrio,s:bacterium New Zealand D |
| OTU_1837 | 0 | 9.25 | 0.75 | k:Bacteria,p:Fusobacteriota,c:Fusobacteriia,o:Fusobacteriales,f:Fusobacteriaceae,g:Fusobacterium |
| OTU_1841 | 0 | 0 | 0.75 | k:Bacteria,p:Actinobacteriota,c:Actinobacteria,o:Propionibacteriales,f:Nocardioidaceae,g:Nocardioides,s:Nocardioides ginkgobilobae |
| OTU_1845 | 0 | 1.5 | 0 | k:Bacteria,p:Actinobacteriota,c:Coriobacteriia,o:Coriobacteriales,f:Eggerthellaceae,g:Slackia,s:Slackia isoflavoniconvertens |
| OTU_18 | 4.5 | 1.5 | 1138 | k:Bacteria,p:Firmicutes,c:Clostridia,o:Lachnospirales,f:Lachnospiraceae,g:Lachnospiraceae NK4A136,s:uncultured |
| OTU_1846 | 0 | 0 | 0.5 | k:Bacteria,p:Elusimicrobiota,c:Elusimicrobia,o:Elusimicrobiales,f:Elusimicrobiaceae,g:Elusimicrobium,s:uncultured |
| OTU_185 | 0 | 44.5 | 4 | k:Bacteria,p:Bacteroidetes,c:Bacteroidia,o:Bacteroidales,f:Muribaculaceae,g:Duncaniella,s:Duncaniella muris |
| OTU_1851 | 0 | 0 | 0.5 | k:Bacteria,p:Proteobacteria,c:Gammaproteobacteria,o:Burkholderiales,f:Burkholderiaceae,g:Burkholderia,s:Pseudomonas mesoacidophila |
| OTU_1852 | 0 | 0 | 1 | k:Bacteria,p:Firmicutes,c:Bacilli,o:Staphylococcales,f:Staphylococcaceae,g:Jeotgalicoccus,s:Jeotgalicoccus pinnipedialis |
| OTU_186 | 0 | 3.25 | 26 | k:Bacteria,p:Firmicutes,c:Clostridia,o:Borkfalkiales,f:PAC001219,g:f;PAC001219 |
| OTU_1866 | 0 | 0 | 0.5 | k:Bacteria,p:Firmicutes,c:Clostridia,o:Clostridiales,f:Lachnospiraceae,g:Fusimonas,s:uncultured |
| OTU_1867 | 0 | 3.5 | 0 | k:Bacteria,p:Firmicutes,c:Clostridia,o:Christensenellales,f:Christensenellaceae,g:Christensenellaceae R-7,s:uncultured |
| OTU_1870 | 0 | 0 | 1.5 | k:Bacteria,p:Bacteroidetes,c:Bacteroidia,o:Bacteroidales,f:Muribaculaceae,g:RIAY |
| OTU_1873 | 0 | 0 | 0.75 | k:Bacteria,p:Verrucomicrobiota,c:Verrucomicrobiae,o:Chthoniobacterales,f:Chthoniobacteraceae,g:Candidatus Udaeobacter |
| OTU_1875 | 0 | 0 | 0.5 | k:Bacteria,p:Firmicutes,c:Clostridia,o:Clostridiales,f:Lachnospiraceae,g:PAC001199 |
| OTU_1876 | 0 | 7.5 | 0 | k:Bacteria,p:Firmicutes,c:Clostridia,o:Lachnospirales,f:Lachnospiraceae,g:Ruminococcus torques,s:uncultured |
| OTU_1880 | 0 | 1.5 | 0 | k:Bacteria,p:Firmicutes,c:Clostridia,o:Peptococcales,f:Peptococcaceae,g:uncultured |
| OTU_188 | 0 | 55.5 | 0 | k:Bacteria,p:Actinobacteriota,c:Actinobacteria,o:Micrococcales,f:Micrococcaceae,g:Glutamicibacter |
| OTU_1881 | 0 | 1.5 | 0.5 | k:Bacteria,p:Firmicutes,c:Bacilli,o:Erysipelotrichales,f:Erysipelotrichaceae,g:Turicibacter,s:uncultured |
| OTU_1882 | 0 | 0 | 0.5 | k:Bacteria,p:Bacteroidota,c:Bacteroidia,o:Bacteroidales,f:Muribaculaceae |
| OTU_1883 | 0 | 1 | 0.25 | k:Bacteria,p:Firmicutes,c:Clostridia,o:Oscillospirales,f:Ruminococcaceae,g:uncultured |
| OTU_1884 | 0 | 0 | 1.5 | k:Bacteria,p:Firmicutes,c:Clostridia,o:Oscillospirales,f:Ruminococcaceae,g:Harryflintia,s:uncultured |
| OTU_1886 | 0 | 2.75 | 0.25 | k:Bacteria,p:Firmicutes,c:Clostridia,o:Clostridiales,f:Oscillospiraceae,g:Harryflintia,s:uncultured |
| OTU_1890 | 0 | 6.25 | 0 | k:Bacteria,p:Proteobacteria,c:Gammaproteobacteria,o:Pseudomonadales,f:Moraxellaceae,g:Acinetobacter,s:Acinetobacter johnsonii |
| OTU_1891 | 0 | 0 | 1.5 | k:Bacteria,p:Firmicutes,c:Clostridia,o:Clostridiales,f:Lachnospiraceae,g:RAZD |
| OTU_1892 | 0 | 0.5 | 0 | k:Bacteria,p:Proteobacteria,c:Gammaproteobacteria,o:Enterobacterales,f:Morganellaceae,g:Providencia,s:Providencia rettgeri |
| OTU_1895 | 0 | 0 | 1.25 | k:Bacteria,p:Firmicutes,c:Bacilli,o:Lactobacillales,f:Lactobacillaceae,g:Lactobacillus,s:Lactobacillus gasseri |
| OTU_1896 | 0 | 1 | 0 | k:Bacteria,p:Acidobacteriota,c:Blastocatellia,o:Pyrinomonadales,f:Pyrinomonadaceae,g:RB41,s:uncultured |
| OTU_189 | 45 | 19.25 | 26.5 | k:Bacteria,p:Firmicutes,c:Bacilli,o:Lactobacillales,f:Enterococcaceae,g:Enterococcus,s:Enterococcus faecalis |
| OTU_190 | 0 | 19.5 | 0 | k:Bacteria,p:Firmicutes,c:Clostridia,o:Oscillospirales,f:Ruminococcaceae,g:uncultured |
| OTU_1901 | 0 | 0 | 1.5 | k:Bacteria,p:Firmicutes,c:Clostridia,o:Christensenellales,f:Christensenellaceae,g:Christensenellaceae R-7 group,s:uncultured |
| OTU_1903 | 0 | 0 | 0.75 | k:Bacteria,p:Firmicutes,c:Clostridia,o:Oscillospirales,f:Oscillospiraceae |
| OTU_1904 | 0 | 0.5 | 1.75 | k:Bacteria,p:Firmicutes,c:Clostridia,o:Clostridiales,f:Lachnospiraceae,g:PAC002153 |
| OTU_1907 | 0 | 0 | 16 | k:Bacteria,p:Firmicutes,c:Clostridia,o:Lachnospirales,f:Lachnospiraceae,g:[Eubacterium] xylanophilum group,s:uncultured |
| OTU_1908 | 0 | 0 | 0.5 | k:Bacteria,p:Firmicutes,c:Clostridia,o:Clostridiales,f:Oscillospiraceae,g:Pseudoflavonifractor,s:uncultured |
| OTU_191 | 1.5 | 13.75 | 23.25 | k:Bacteria,p:Firmicutes,c:Clostridia,o:Oscillospirales,f:Oscillospiraceae |
| OTU_1915 | 0 | 0 | 0.5 | k:Bacteria,p:Firmicutes,c:Clostridia,o:Oscillospirales,f:Oscillospiraceae |
| OTU_19 | 80 | 362.5 | 1180 | k:Bacteria,p:Firmicutes,c:Bacilli,o:Lactobacillales,f:Lactobacillaceae,g:Lactobacillus,s:Lactobacillus kitasatonis |
| OTU_1918 | 0 | 0 | 0.5 | k:Bacteria,p:Firmicutes,c:Clostridia,o:Borkfalkiales,f:Borkfalkiaceae,g:uncultured |
| OTU_1921 | 0 | 0 | 1 | k:Bacteria,p:Firmicutes,c:Clostridia,o:Clostridiales,f:Lachnospiraceae,g:Eubacterium,s:uncultured |
| OTU_1926 | 0 | 0 | 1 | k:Bacteria,p:Firmicutes,c:Clostridia,o:Borkfalkiales,f:PAC001219,g:JX095379 |
| OTU_1927 | 0 | 1.75 | 0 | k:Bacteria,p:Firmicutes,c:Clostridia,o:Christensenellales,f:Christensenellaceae,g:Christensenella,s:Christensenella minuta |
| OTU_1930 | 0 | 0.5 | 1.75 | k:Bacteria,p:Actinobacteriota,c:Actinobacteria,o:Micrococcales,f:Promicromonosporaceae |
| OTU_193 | 0 | 10.75 | 24 | k:Bacteria,p:Firmicutes,c:Clostridia,o:Lachnospirales,f:Lachnospiraceae,g:Lachnospirale,s:uncultured |
| OTU_1932 | 0 | 2.25 | 0.75 | k:Bacteria,p:Firmicutes,c:Clostridia,o:Clostridiales,f:Lachnospiraceae,g:GU324393 |
| OTU_1933 | 0 | 0.5 | 0 | k:Bacteria,p:Firmicutes,c:Clostridia,o:Peptostreptococcales-Tissierellales,g:Anaerococcus,s:uncultured |
| OTU_1937 | 0 | 2.75 | 0 | k:Bacteria,p:Bacteroidota,c:Bacteroidia,o:Bacteroidales,f:Muribaculaceae |
| OTU_1938 | 0 | 0 | 1.25 | k:Bacteria,p:Firmicutes,c:Clostridia,o:Oscillospirales,f:Ruminococcaceae,g:Incertae sedis,s:uncultured |
| OTU_194 | 0 | 11.5 | 5.25 | k:Bacteria,p:Proteobacteria,c:Gammaproteobacteria,o:Rhodocyclales,f:Rhodocyclaceae,g:Azospira,s:Azospira oryzae |
| OTU_1941 | 0 | 0 | 1 | k:Bacteria,p:Firmicutes,c:Clostridia,o:Oscillospirales,f:Ruminococcaceae,g:Ruminococcus,s:uncultured |
| OTU_1943 | 0 | 0 | 0.75 | k:Bacteria,p:Bacteroidota,c:Ignavibacteria,o:Ignavibacteriales,f:Ignavibacteriaceae,g:Ignavibacterium,s:uncultured |
| OTU_1948 | 0 | 0 | 14.25 | k:Bacteria,p:Firmicutes,c:Clostridia,o:Clostridiales,f:Lachnospiraceae,g:Enterocloster,s:uncultured |
| OTU_1950 | 0 | 0 | 1 | k:Bacteria,p:Actinobacteriota,c:Coriobacteriia,o:Coriobacteriales,f:Eggerthellaceae,g:Enterorhabdus,s:uncultured |
| OTU_1953 | 0 | 0 | 4.25 | k:Bacteria,p:Firmicutes,c:Clostridia,o:Lachnospirales,f:Lachnospiraceae,g:uncultured |
| OTU_1955 | 0 | 1.25 | 0 | k:Bacteria,p:Firmicutes,c:Clostridia,o:Peptococcales,f:Peptococcaceae,g:Peptococcus,s:uncultured |
| OTU_1957 | 0 | 0 | 0.5 | k:Bacteria,p:Firmicutes,c:Clostridia,o:Clostridiales,f:Oscillospiraceae,g:Pseudoflavonifractor,s:uncultured |
| OTU_196 | 17 | 16.25 | 19 | k:Bacteria,p:Firmicutes,c:Bacilli,o:Lactobacillales,f:Streptococcaceae,g:Lactococcus,s:Lactococcus hordniae |
| OTU_1962 | 0 | 0 | 0.5 | k:Bacteria,p:Firmicutes,c:Clostridia,o:Oscillospirales,f:Oscillospiraceae |
| OTU_1964 | 0 | 0 | 1 | k:Bacteria,p:Bacteroidota,c:Bacteroidia,o:Bacteroidales,f:Muribaculaceae |
| OTU_1968 | 0 | 0 | 2 | k:Bacteria,p:Firmicutes,c:Clostridia,o:Lachnospirales,f:Lachnospiraceae,g:uncultured |
| OTU_1969 | 0 | 0.75 | 0 | k:Bacteria,p:Proteobacteria,c:Gammaproteobacteria,o:Burkholderiales,f:Comamonadaceae,g:Acidovorax,s:Acidovorax antarcticus |
| OTU_1974 | 0 | 1.5 | 0 | k:Bacteria,p:Actinobacteriota,c:Actinobacteria,o:Actinomycetales,f:Actinomycetaceae,g:Flaviflexus,s:uncultured |
| OTU_1975 | 0 | 0 | 0.75 | k:Bacteria,p:Firmicutes,c:Clostridia,o:Christensenellales,f:Christensenellaceae,g:Christensenellaceae R-7 group,s:uncultured |
| OTU_1978 | 0 | 0.75 | 0 | k:Bacteria,p:Actinobacteriota,c:Coriobacteriia,o:Coriobacteriales,f:Atopobiaceae,g:Olsenella |
| OTU_1980 | 0 | 0 | 1.25 | k:Bacteria,p:Desulfobacterota,c:Desulfovibrionia,o:Desulfovibrionales,f:Desulfovibrionaceae,g:Desulfovibrio,s:Desulfovibrio fairfieldensis |
| OTU_198 | 0 | 14.5 | 4.75 | k:Bacteria,p:Actinobacteriota,c:Coriobacteriia,o:Coriobacteriales,f:uncultured |
| OTU_1983 | 0 | 0 | 0.5 | k:Bacteria,p:Bacteroidota,c:Bacteroidia,o:Bacteroidales |
| OTU_1984 | 0 | 1 | 0 | k:Bacteria,p:Actinobacteriota,c:Coriobacteriia,o:Coriobacteriales,f:Atopobiaceae |
| OTU_1986 | 0 | 3 | 0 | k:Bacteria,p:Firmicutes,c:Bacilli,o:Erysipelotrichales,f:Erysipelatoclostridiaceae,g:Erysipelatoclostridium,s:Thomasclavelia ramosa |
| OTU_1987 | 0 | 0 | 0.5 | k:Bacteria,p:Bacteroidota,c:Bacteroidia,o:Bacteroidales,f:Muribaculaceae |
| OTU_1989 | 0 | 4.75 | 36 | k:Bacteria,p:Firmicutes,c:Clostridia,o:Oscillospirales,f:Oscillospiraceae,g:uncultured |
| OTU_199 | 0 | 0 | 39.25 | k:Bacteria,p:Firmicutes,c:Clostridia,o:Clostridia UCG-014 |
| OTU_1991 | 0 | 0 | 0.5 | k:Bacteria,p:Firmicutes,c:Clostridia,o:Borkfalkiales,f:Borkfalkiaceae,g:PAC001371,s:uncultured |
| OTU_1994 | 0 | 0 | 3.5 | k:Bacteria,p:Firmicutes,c:Bacilli,o:RF39 |
| OTU_1996 | 0 | 0.25 | 1.25 | k:Bacteria,p:Firmicutes,c:Clostridia,o:Clostridiales,f:Lachnospiraceae,g:PAC001092 |
| OTU_1998 | 0 | 0 | 0.5 | k:Bacteria,p:Proteobacteria,c:Gammaproteobacteria,o:Enterobacterales,f:Enterobacteriaceae,g:Enterobacter,s:Enterobacter vonholyi |
| OTU_2002 | 0 | 0 | 0.75 | k:Bacteria,p:Firmicutes,c:Clostridia,o:Clostridiales,f:Lachnospiraceae,g:PAC001043 |
| OTU_20 | 1 | 0.75 | 780 | k:Bacteria,p:Bacteroidota,c:Bacteroidia,o:Bacteroidales,f:F082,g:uncultured |
| OTU_2003 | 0 | 0 | 0.5 | k:Bacteria,p:Firmicutes,c:Bacilli,o:Lactobacillales,f:Lactobacillaceae,g:Lactobacillus,s:Lactobacillus johnsonii |
| OTU_2005 | 0 | 1 | 0 | k:Bacteria,p:Firmicutes,c:Bacilli,o:Lactobacillales,f:Lactobacillaceae,g:Lactobacillus,s:Latilactobacillus sakei subsp. carnosus |
| OTU_204 | 0 | 28.5 | 0 | k:Bacteria,p:Actinobacteriota,c:Coriobacteriia,o:Coriobacteriales,f:Atopobiaceae,g:Olsenella,s:uncultured |
| OTU_205 | 0.5 | 34.75 | 0.75 | k:Bacteria,p:Firmicutes,c:Clostridia,o:Lachnospirales,f:Lachnospiraceae,g:Roseburia,s:uncultured |
| OTU_206 | 0 | 21 | 2 | k:Bacteria,p:Bacteroidota,c:Bacteroidia,o:Bacteroidales,f:Muribaculaceae |
| OTU_207 | 0 | 2 | 11.75 | k:Bacteria,p:Firmicutes,c:Clostridia,o:Oscillospirales,f:UCG-010 |
| OTU_208 | 0 | 0.25 | 46 | k:Bacteria,p:Bacteroidota,c:Bacteroidia,o:Bacteroidales,f:Muribaculaceae |
| OTU_209 | 0 | 2.75 | 41.75 | k:Bacteria,p:Firmicutes,c:Clostridia,o:Lachnospirales,f:Lachnospiraceae,g:uncultured |
| OTU_210 | 0 | 0 | 17 | k:Bacteria,p:Firmicutes,c:Clostridia,o:Oscillospirales,f:Ruminococcaceae,g:uncultured |
| OTU_211 | 0.5 | 1.5 | 46.25 | k:Bacteria,p:Firmicutes,c:Clostridia,o:Clostridiales,f:Mogibacterium,g:Lentihominibacter,s:uncultured |
| OTU_212 | 0 | 24.75 | 38.75 | k:Bacteria,p:Firmicutes,c:Clostridia,o:Oscillospirales,f:Oscillospiraceae,g:Oscillibacter,s:uncultured |
| OTU_213 | 0 | 60 | 2.25 | k:Bacteria,p:Firmicutes,c:Clostridia,o:Lachnospirales,f:Lachnospiraceae,g:Roseburia |
| OTU_21 | 2 | 61.75 | 922.25 | k:Bacteria,p:Firmicutes,c:Clostridia,o:Lachnospirales,f:Lachnospiraceae,g:Lachnospiraceae,s:Lachnospiraceae bacterium |
| OTU_216 | 0 | 0 | 44.5 | k:Bacteria,p:Bacteroidota,c:Bacteroidia,o:Bacteroidales,f:Muribaculaceae |
| OTU_217 | 1 | 0 | 57.5 | k:Bacteria,p:Firmicutes,c:Clostridia,o:Oscillospirales,f:Oscillospiraceae,g:uncultured |
| OTU_218 | 0 | 20.5 | 0 | k:Bacteria,p:Bacteroidota,c:Bacteroidia,o:Bacteroidales,f:Muribaculaceae |
| OTU_219 | 0 | 18.25 | 1.5 | k:Bacteria,p:Bacteroidota,c:Bacteroidia,o:Bacteroidales,f:Muribaculaceae |
| OTU_220 | 0 | 37.75 | 6.75 | k:Bacteria,p:Desulfobacterota,c:Desulfovibrionia,o:Desulfovibrionales,f:Desulfovibrionaceae,g:Desulfovibrio,s:uncultured |
| OTU_221 | 0 | 21.25 | 0 | k:Bacteria,p:Firmicutes,c:Clostridia,o:Clostridiales,f:Lachnospiraceae,g:Fusicatenibacter,s:Fusicatenibacter saccharivorans |
| OTU_222 | 0 | 0.5 | 20 | k:Bacteria,p:Firmicutes,c:Clostridia,o:Oscillospirales,f:Oscillospiraceae,g:NK4A214 |
| OTU_22 | 166.5 | 28.5 | 395.75 | k:Bacteria,p:Proteobacteria,c:Gammaproteobacteria,o:Burkholderiales,f:Burkholderiaceae,g:Cupriavidus,s:Cupriavidus metallidurans |
| OTU_225 | 0 | 16.75 | 0 | k:Bacteria,p:Fusobacteriota,c:Fusobacteriia,o:Fusobacteriales,f:Leptotrichiaceae,g:Streptobacillus,s:Streptobacillus moniliformis |
| OTU_227 | 1.5 | 1.75 | 13.25 | k:Bacteria,p:Proteobacteria,c:Gammaproteobacteria,o:Pseudomonadales,f:Moraxellaceae,g:Acinetobacter,s:Acinetobacter bereziniae |
| OTU_228 | 0 | 23.75 | 44.5 | k:Bacteria,p:Bacteroidota,c:Bacteroidia,o:Bacteroidales,f:Muribaculaceae |
| OTU_230 | 0 | 12.75 | 0 | k:Bacteria,p:Proteobacteria,c:Gammaproteobacteria,o:Burkholderiales,f:Neisseriaceae,g:Neisseria,s:Neisseria weixii |
| OTU_23 | 729.5 | 0.75 | 0.5 | k:Bacteria,p:Proteobacteria,c:Alphaproteobacteria,o:Rhizobiales,f:Rhizobiaceae,g:Bartonella,s:Bartonella australis |
| OTU_232 | 8 | 15.25 | 3 | k:Bacteria,p:Firmicutes,c:Bacilli,o:Lactobacillales,f:Enterococcaceae,g:Vagococcus,s:Vagococcus xieshaowenii |
| OTU_234 | 0 | 8 | 29.75 | k:Bacteria,p:Bacteroidota,c:Bacteroidia,o:Bacteroidales,f:Muribaculaceae |
| OTU_239 | 23 | 47.5 | 5.75 | k:Bacteria,p:Firmicutes,c:Bacilli,o:Staphylococcales,f:Gemellaceae,g:Gemella,s:Gemella sanguinis |
| OTU_240 | 0 | 22.75 | 0 | k:Bacteria,p:Firmicutes,c:Bacilli,o:Lactobacillales,f:Streptococcaceae,g:Streptococcus,s:Streptococcus caballi |
| OTU_242 | 0 | 6.25 | 19.5 | k:Bacteria,p:Bacteroidota,c:Bacteroidia,o:Bacteroidales,f:Muribaculaceae |
| OTU_243 | 0 | 42 | 0 | k:Bacteria,p:Deferribacterota,c:Deferribacteres,o:Deferribacterales,f:Deferribacteraceae,g:Mucispirillum |
| OTU_245 | 0 | 0 | 20.25 | k:Bacteria,p:Bacteroidota,c:Bacteroidia,o:Bacteroidales,f:Rs-E47 termite group |
| OTU_246 | 0 | 2.75 | 12 | k:Bacteria,p:Firmicutes,c:Bacilli,o:Acholeplasmatales,f:Acholeplasmataceae,g:Anaeroplasma,s:uncultured |
| OTU_247 | 0 | 9.25 | 22.75 | k:Bacteria,p:Firmicutes,c:Clostridia,o:Oscillospirales,f:Oscillospiraceae,g:UCG-005,s:uncultured |
| OTU_249 | 0 | 0 | 19 | k:Bacteria,p:Firmicutes,c:Clostridia,o:Borkfalkiales,f:PAC001219,g:GQ448104 |
| OTU_250 | 0 | 26.25 | 7.25 | k:Bacteria,p:Firmicutes,c:Clostridia,o:Clostridiales,f:Lachnospiraceae,g:Enterocloster,s:uncultured |
| OTU_25 | 0 | 619 | 0 | k:Bacteria,p:Firmicutes,c:Negativicutes,o:Veillonellales-Selenomonadales,f:Veillonellaceae,g:Veillonella,s:uncultured |
| OTU_251 | 0 | 4 | 24.75 | k:Bacteria,p:Bacteroidota,c:Bacteroidia,o:Bacteroidales,f:Muribaculaceae |
| OTU_252 | 0 | 32.75 | 0 | k:Bacteria,p:Bacteroidota,c:Bacteroidia,o:Bacteroidales,f:Porphyromonadaceae,g:Porphyromonas,s:uncultured |
| OTU_253 | 0 | 0 | 12.75 | k:Bacteria,p:Proteobacteria,c:Alphaproteobacteria,o:Sphingomonadales,f:Sphingomonadaceae |
| OTU_2 | 76.5 | 20941.5 | 3616.5 | k:Bacteria,p:Firmicutes,c:Bacilli,o:Lactobacillales,f:Lactobacillaceae,g:Lactobacillus,s:Lactobacillus faecis |
| OTU_254 | 0 | 17.75 | 0 | k:Bacteria,p:Firmicutes,c:Bacilli,o:Erysipelotrichales,f:Erysipelotrichaceae,g:Allobaculum,s:uncultured |
| OTU_256 | 0 | 6.75 | 11.75 | k:Bacteria,p:Proteobacteria,c:Alphaproteobacteria,o:Rhizobiales,f:Rhizobiaceae,g:Allorhizobium-Neorhizobium-Pararhizobium-Rhizobium |
| OTU_257 | 0 | 54.5 | 23 | k:Bacteria,p:Firmicutes,c:Clostridia,o:Clostridiales,f:Lachnospiraceae,g:KE159797 |
| OTU_260 | 0 | 0 | 20.25 | k:Bacteria,p:Firmicutes,c:Clostridia,o:Lachnospirales,f:Lachnospiraceae,g:uncultured |
| OTU_26 | 0 | 405.5 | 0 | k:Bacteria,p:Bacteroidota,c:Bacteroidia,o:Bacteroidales,f:Bacteroidaceae,g:Bacteroides,uncultured |
| OTU_261 | 0 | 0 | 32.25 | k:Bacteria,p:Bacteroidota,c:Bacteroidia,o:Bacteroidales,f:Prevotellaceae,g:Prevotellaceae UCG-001,s:uncultured |
| OTU_262 | 0 | 19.25 | 31.75 | k:Bacteria,p:Firmicutes,c:Clostridia,o:Lachnospirales,f:Lachnospiraceae,g:uncultured |
| OTU_263 | 0 | 3.75 | 19.25 | k:Bacteria,p:Proteobacteria,c:Alphaproteobacteria,o:Rhizobiales,f:Xanthobacteraceae |
| OTU_264 | 0 | 12.75 | 5 | k:Bacteria,p:Actinobacteriota,c:Coriobacteriia,o:Coriobacteriales,f:Eggerthellaceae,g:Enterorhabdus,s:uncultured |
| OTU_266 | 0 | 21 | 3.75 | k:Bacteria,p:Actinobacteriota,c:Actinobacteria,o:Micrococcales,f:Dermabacteraceae,g:Brachybacterium |
| OTU_267 | 0 | 12.75 | 3.75 | k:Bacteria,p:Firmicutes,c:Clostridia,o:Oscillospirales,f:Ruminococcaceae,g:Incertae Sedis,s:uncultured |
| OTU_269 | 0 | 0.5 | 10.5 | k:Bacteria,p:Proteobacteria,c:Gammaproteobacteria,o:Pseudomonadales,f:Pseudomonadaceae,g:Pseudomonas,s:Pseudomonas mendocina |
| OTU_270 | 2 | 24.75 | 0 | k:Bacteria,p:Actinobacteriota,c:Actinobacteria,o:Micrococcales,f:Brevibacteriaceae,g:Brevibacterium |
| OTU_27 | 0.5 | 481 | 1.5 | k:Bacteria,p:Firmicutes,c:Bacilli,o:Bacillales,f:Bacillaceae,g:Bacillus,s:Bacillus thuringiensis |
| OTU_272 | 0 | 15.25 | 31 | k:Bacteria,p:Bacteroidota,c:Bacteroidia,o:Bacteroidales,f:Muribaculaceae |
| OTU_273 | 0 | 28 | 9.25 | k:Bacteria,p:Bacteroidota,c:Bacteroidia,o:Bacteroidales,f:Muribaculaceae |
| OTU_275 | 0 | 9 | 45.75 | k:Bacteria,p:Firmicutes,c:Clostridia,o:Lachnospirales,f:Lachnospiraceae,g:Lachnospiraceae NK4A136,s:uncultured |
| OTU_277 | 0 | 15.75 | 0.5 | k:Bacteria,p:Actinobacteriota,c:Actinobacteria,o:Corynebacteriales,f:Corynebacteriaceae,g:Corynebacterium,s:Corynebacterium zhongnanshanii |
| OTU_278 | 0 | 0.75 | 58.25 | k:Bacteria,p:Firmicutes,c:Clostridia,o:Oscillospirales,f:Oscillospiraceae |
| OTU_279 | 0 | 18.25 | 0.25 | k:Bacteria,p:Firmicutes,c:Bacilli,o:Staphylococcales,f:Staphylococcaceae,g:Jeotgalicoccus |
| OTU_280 | 0 | 0.5 | 11.75 | k:Bacteria,p:Firmicutes,c:Clostridia,o:Oscillospirales,f:Ruminococcaceae,g:uncultured |
| OTU_28 | 0.5 | 309.25 | 26.25 | k:Bacteria,p:Proteobacteria,c:Gammaproteobacteria,o:Pseudomonadales,f:Moraxellaceae,g:Acinetobacter,s:Acinetobacter lwoffii |
| OTU_282 | 0 | 0 | 36.75 | k:Bacteria,p:Firmicutes,c:Clostridia,o:Clostridiales,f:Lachnospiraceae,g:PAC001043 |
| OTU_283 | 0 | 0 | 18.75 | k:Bacteria,p:Bacteroidota,c:Bacteroidia,o:Bacteroidales,f:Muribaculaceae |
| OTU_286 | 0 | 0.25 | 15 | k:Bacteria,p:Firmicutes,c:Clostridia,o:Oscillospirales,f:Ruminococcaceae,g:uncultured |
| OTU_288 | 1 | 5.5 | 47.75 | k:Bacteria,p:Firmicutes,c:Clostridia,o:Clostridia vadinBB60 |
| OTU_290 | 0 | 6 | 8 | k:Bacteria,p:Actinobacteriota,c:Coriobacteriia,o:Coriobacteriales,f:Eggerthellaceae |
| OTU_291 | 0 | 13 | 10.5 | k:Bacteria,p:Proteobacteria,c:Gammaproteobacteria,o:Pseudomonadales,f:Moraxellaceae,g:Acinetobacter,s:Acinetobacter haemolyticus |
| OTU_293 | 0 | 3.25 | 13 | k:Bacteria,p:Firmicutes,c:Clostridia,o:Oscillospirales,f:Ruminococcaceae,g:uncultured |
| OTU_294 | 0.5 | 51.75 | 110.5 | k:Bacteria,p:Firmicutes,c:Clostridia,o:Lachnospirales,f:Lachnospiraceae,g:Lachnospiraceae NK4A136 |
| OTU_295 | 28.5 | 1 | 0.5 | k:Bacteria,p:Firmicutes,c:Bacilli,o:Mycoplasmatales,f:Mycoplasmataceae,g:Mycoplasma,s:Metamycoplasma sualvi |
| OTU_296 | 0 | 14.5 | 0 | k:Bacteria,p:Actinobacteriota,c:Coriobacteriia,o:Coriobacteriales,f:Coriobacteriaceae,g:Collinsella,s:uncultured |
| OTU_29 | 51.5 | 33.75 | 167 | k:Bacteria,p:Proteobacteria,c:Alphaproteobacteria,o:Sphingomonadales,f:Sphingomonadaceae,g:Sphingomonas,s:Sphingomonas pituitosa |
| OTU_297 | 0 | 0 | 19 | k:Bacteria,p:Bacteroidota,c:Bacteroidia,o:Bacteroidales,f:Muribaculaceae |
| OTU_300 | 0 | 13.25 | 0.25 | k:Bacteria,p:Firmicutes,c:Clostridia,o:Lachnospirales,f:Lachnospiraceae,g:Blautia,s:uncultured |
| OTU_301 | 0 | 1.75 | 6.75 | k:Bacteria,p:Proteobacteria,c:Alphaproteobacteria,o:Rhodobacterales,f:Rhodobacteraceae,g:Paracoccus,s:Paracoccus sulfuroxidans |
| OTU_302 | 0 | 4 | 9.25 | k:Bacteria,p:Firmicutes,c:Clostridia,o:Peptostreptococcales-Tissierellales,f:Anaerovoracaceae,g:[Eubacterium] brachy group,s:uncultured |
| OTU_303 | 0 | 0 | 10.5 | k:Bacteria,p:Firmicutes,c:Clostridia,o:Borkfalkiales,f:PAC001219,g:GQ448104 |
| OTU_304 | 0 | 17.5 | 0 | k:Bacteria,p:Bacteroidota,c:Bacteroidia,o:Bacteroidales,f:Prevotellaceae |
| OTU_305 | 0 | 7.5 | 6.5 | k:Bacteria,p:Firmicutes,c:Clostridia,o:Peptostreptococcales-Tissierellales,f:Anaerovoracaceae,g:Family XIII AD3011 group,s:uncultured |
| OTU_306 | 4.5 | 11.75 | 1.5 | k:Bacteria,p:Proteobacteria,c:Alphaproteobacteria,o:Caulobacterales,f:Caulobacteraceae,g:Brevundimonas,s:Brevundimonas diminuta |
| OTU_307 | 0 | 1.5 | 31.5 | k:Bacteria,p:Bacteroidota,c:Bacteroidia,o:Bacteroidales,f:Muribaculaceae |
| OTU_308 | 0 | 7.25 | 6.75 | k:Bacteria,p:Bacteroidota,c:Bacteroidia,o:Bacteroidales,f:Bacteroidaceae,g:Bacteroides,s:uncultured |
| OTU_31 | 0 | 296 | 0 | k:Bacteria,p:Proteobacteria,c:Gammaproteobacteria,o:Pasteurellales,f:Pasteurellaceae,g:Actinobacillus,s:uncultured |
| OTU_311 | 0 | 11 | 0 | k:Bacteria,p:Firmicutes,c:Clostridia,o:Lachnospirales,f:Lachnospiraceae,g:Roseburia,s:uncultured |
| OTU_313 | 0 | 4 | 5.75 | k:Bacteria,p:Firmicutes,c:Clostridia,o:Peptostreptococcales-Tissierellales,f:Peptostreptococcaceae,g:Peptostreptococcus,s:Peptostreptococcus anaerobius |
| OTU_314 | 0 | 14 | 15 | k:Bacteria,p:Firmicutes,c:Clostridia,o:Lachnospirales,f:Lachnospiraceae,g:uncultured |
| OTU_316 | 4 | 0.75 | 7.25 | k:Bacteria,p:Firmicutes,c:Clostridia,o:Clostridiales,f:Lachnospiraceae,g:Suipraeoptans,s:Suipraeoptans intestinalis |
| OTU_318 | 0 | 0 | 9.25 | k:Bacteria,p:Firmicutes,c:Clostridia,o:Clostridia UCG-014 |
| OTU_319 | 0 | 12 | 0 | k:Bacteria,p:Bacteroidota,c:Bacteroidia,o:Bacteroidales,f:Bacteroidaceae,g:Bacteroides,s:Bacteroides fluxus |
| OTU_32 | 59.5 | 302.5 | 9.25 | k:Bacteria,p:Proteobacteria,c:Gammaproteobacteria,o:Enterobacterales,f:Enterobacteriaceae,g:Enterobacter,s:Enterobacter cancerogenus |
| OTU_322 | 0 | 14.25 | 0 | k:Bacteria,p:Firmicutes,c:Bacilli,o:Lactobacillales,f:Lactobacillaceae,g:Lactobacillus,s:Limosilactobacillus mucosae |
| OTU_324 | 0 | 1.75 | 6 | k:Bacteria,p:Bacteroidota,c:Bacteroidia,o:Bacteroidales,f:Tannerellaceae,g:Parabacteroides,s:Parabacteroides goldsteinii |
| OTU_326 | 0 | 7.75 | 0 | k:Bacteria,p:Bacteroidota,c:Bacteroidia,o:Bacteroidales,f:Muribaculaceae |
| OTU_328 | 0.5 | 0 | 16 | k:Bacteria,p:Firmicutes,c:Clostridia,o:Lachnospirales,f:Lachnospiraceae,g:uncultured |
| OTU_330 | 0 | 28 | 0 | k:Bacteria,p:Bacteroidota,c:Bacteroidia,o:Bacteroidales,f:Muribaculaceae |
| OTU_331 | 0 | 4.75 | 25.75 | k:Bacteria,p:Bacteroidota,c:Bacteroidia,o:Bacteroidales,f:Muribaculaceae |
| OTU_33 | 1.5 | 204.5 | 30 | k:Bacteria,p:Actinobacteriota,c:Actinobacteria,o:Actinomycetales,f:Actinomycetaceae,g:Actinomyces |
| OTU_334 | 0 | 0 | 26.75 | k:Bacteria,p:Firmicutes,c:Clostridia,o:Lachnospirales,f:Lachnospiraceae,g:Lachnospiraceae NK4A136 group,s:uncultured |
| OTU_336 | 0 | 16 | 3.5 | k:Bacteria,p:Firmicutes,c:Clostridia,o:Lachnospirales,f:Lachnospiraceae,g:uncultured |
| OTU_337 | 0 | 24.5 | 0 | k:Bacteria,p:Bacteroidota,c:Bacteroidia,o:Bacteroidales,f:Muribaculaceae |
| OTU_3 | 9874 | 4932.75 | 11078 | k:Bacteria,p:Proteobacteria,c:Gammaproteobacteria,o:Burkholderiales,f:Burkholderiaceae,g:Burkholderia,s:Burkholderia metallica |
| OTU_338 | 0 | 2.25 | 14.25 | k:Bacteria,p:Firmicutes,c:Clostridia,o:Clostridiales,f:Lachnospiraceae,g:PAC001116 |
| OTU_339 | 0 | 0 | 8.25 | k:Bacteria,p:Cyanobacteria,c:Vampirivibrionia,o:Obscuribacterales,f:Obscuribacteraceae |
| OTU_341 | 0 | 4.5 | 13.75 | k:Bacteria,p:Firmicutes,c:Clostridia,o:Oscillospirales,f:Ruminococcaceae,g:uncultured |
| OTU_344 | 0 | 0 | 9.75 | k:Bacteria,p:Firmicutes,c:Clostridia,o:Clostridiales,f:Lachnospiraceae,g:Eisenbergiella,s:uncultured |
| OTU_348 | 0 | 8.5 | 3.75 | k:Bacteria,p:Firmicutes,c:Clostridia,o:Lachnospirales,f:Lachnospiraceae,g:Lachnospiraceae NK4A136,s:uncultured |
| OTU_349 | 0 | 0.25 | 11 | k:Bacteria,p:Firmicutes,c:Clostridia,o:Oscillospirales,f:Butyricicoccaceae,g:UCG009,s:uncultured |
| OTU_350 | 0 | 17.75 | 0.25 | k:Bacteria,p:Bacteroidota,c:Bacteroidia,o:Bacteroidales,f:Bacteroidaceae,g:Bacteroides,s:Bacteroides eggerthii |
| OTU_35 | 19.5 | 18.75 | 185.5 | k:Bacteria,p:Proteobacteria,c:Gammaproteobacteria,o:Burkholderiales,f:Burkholderiaceae,f:Ralstonia,g:Cupriavidus,s:Cupriavidus basilensis |
| OTU_353 | 0 | 3 | 24 | k:Bacteria,p:Firmicutes,c:Clostridia,o:Oscillospirales,f:Ruminococcaceae,g:uncultured |
| OTU_354 | 0 | 8.75 | 0.5 | k:Bacteria,p:Actinobacteriota,c:Coriobacteriia,o:Coriobacteriales,f:Atopobiaceae |
| OTU_355 | 0 | 6.75 | 21.5 | k:Bacteria,p:Firmicutes,c:Clostridia,o:Clostridiales,f:Lachnospiraceae,g:PAC001116 |
| OTU_356 | 0 | 5.25 | 8.25 | k:Bacteria,p:Firmicutes,c:Bacilli,o:Lactobacillales,f:Enterococcaceae,g:Vagococcus,s:Vagococcus fessus |
| OTU_359 | 21.5 | 6.5 | 0 | k:Bacteria,p:Bacteroidota,c:Bacteroidia,o:Bacteroidales,f:Bacteroidaceae,g:Bacteroides,s:uncultured |
| OTU_360 | 0 | 10 | 0 | k:Bacteria,p:Actinobacteriota,c:Actinobacteria,o:Bifidobacteriales,f:Bifidobacteriaceae,g:Bifidobacterium |
| OTU_36 | 0 | 183 | 0 | k:Bacteria,p:Campilobacterota,c:Campylobacteria,o:Campylobacterales,f:Campylobacteraceae,g:Campylobacter,s:Campylobacter hyointestinalis |
| OTU_361 | 0 | 8.25 | 8 | k:Bacteria,p:Patescibacteria,c:Saccharimonadia,o:Saccharimonadales,f:Saccharimonadaceae,g:Candidatus Saccharimonas,s:uncultured |
| OTU_363 | 0 | 0.25 | 8.25 | k:Bacteria,p:Bacteroidota,c:Bacteroidia,o:Bacteroidales,f:Muribaculaceae |
| OTU_364 | 0 | 0 | 10.25 | k:Bacteria,p:Firmicutes,c:Clostridia,o:Lachnospirales,f:Lachnospiraceae,g:uncultured |
| OTU_365 | 0 | 0 | 6.5 | k:Bacteria,p:Chloroflexi,c:JG30-KF-CM66 |
| OTU_366 | 0 | 0 | 13.25 | k:Bacteria,p:Firmicutes,c:Clostridia,o:Lachnospirales,f:Lachnospiraceae,g:Blautia,s:uncultured |
| OTU_368 | 0 | 6.25 | 2 | k:Bacteria,p:Firmicutes,c:Clostridia,o:Lachnospirales,f:Lachnospiraceae,g:Roseburia |
| OTU_369 | 0 | 0.75 | 14 | k:Bacteria,p:Firmicutes,c:Clostridia,o:Monoglobales,f:Monoglobaceae,g:Monoglobus,s:uncultured |
| OTU_37 | 0 | 0 | 357 | k:Bacteria,p:Bacteroidota,c:Bacteroidia,o:Bacteroidales,f:Prevotellaceae,g:Prevotella,s:uncultured |
| OTU_370 | 0 | 3.5 | 4.5 | k:Bacteria,p:Actinobacteriota,c:Coriobacteriia,o:Coriobacteriales,f:Eggerthellaceae,g:Enterorhabdus,s:uncultured |
| OTU_371 | 0 | 22.75 | 0 | k:Bacteria,p:Firmicutes,c:Bacilli,o:Erysipelotrichales,f:Erysipelotrichaceae,g:Allobaculum |
| OTU_373 | 0 | 0.5 | 6.5 | k:Bacteria,p:Firmicutes,c:Clostridia,o:Clostridiales,f:Lachnospiraceae,g:RAYR |
| OTU_375 | 0 | 0.5 | 20.75 | k:Bacteria,p:Firmicutes,c:Clostridia,o:Lachnospirales,f:Lachnospiraceae,g:uncultured |
| OTU_376 | 0 | 2 | 22 | k:Bacteria,p:Firmicutes,c:Clostridia,o:Oscillospirales,f:Oscillospiraceae,g:uncultured |
| OTU_378 | 0 | 0.25 | 11.25 | k:Bacteria,p:Bacteroidota,c:Bacteroidia,o:Bacteroidales,f:Muribaculaceae |
| OTU_38 | 0 | 2.5 | 257.75 | k:Bacteria,p:Firmicutes,c:Clostridia,o:Lachnospirales,f:Lachnospiraceae,g:Lachnospiraceae NK4A136 group,s:uncultured |
| OTU_386 | 0 | 6 | 1 | k:Bacteria,p:Firmicutes,c:Clostridia,o:Oscillospirales,f:Ruminococcaceae,g:Ruminococcus |
| OTU_388 | 0 | 3.5 | 4.75 | k:Bacteria,p:Actinobacteriota,c:Coriobacteriia,o:Coriobacteriales,f:Eggerthellaceae,g:Enterorhabdus |
| OTU_390 | 12 | 0 | 19.75 | k:Bacteria,p:Proteobacteria,c:Alphaproteobacteria,o:Rhizobiales,f:Rhizobiaceae,g:Allorhizobium-Neorhizobium-Pararhizobium-Rhizobium |
| OTU_391 | 0 | 2 | 16.5 | k:Bacteria,p:Firmicutes,c:Clostridia,o:Oscillospirales,f:Ruminococcaceae,g:[Eubacterium] siraeum group,s:uncultured |
| OTU_39 | 17 | 95 | 107.75 | k:Bacteria,p:Actinobacteriota,c:Actinobacteria,o:Micrococcales,f:Micrococcaceae,g:Rothia |
| OTU_393 | 17 | 0 | 0.75 | k:Bacteria,p:Proteobacteria,c:Alphaproteobacteria,o:Rhizobiales,f:Xanthobacteraceae,g:uncultured |
| OTU_395 | 0 | 0.25 | 19.5 | k:Bacteria,p:Bacteroidota,c:Bacteroidia,o:Bacteroidales,f:Muribaculaceae |
| OTU_400 | 0 | 1.75 | 4.5 | k:Bacteria,p:Proteobacteria,c:Gammaproteobacteria,o:Burkholderiales,f:Oxalobacteraceae,g:Oxalobacter,s:uncultured |
| OTU_4 | 0 | 17244.25 | 0.25 | k:Bacteria,p:Firmicutes,c:Bacilli,o:Lactobacillales,f:Streptococcaceae,g:Streptococcus,s:Streptococcus hyointestinalis |
| OTU_403 | 0 | 8.5 | 0 | k:Bacteria,p:Bacteroidota,c:Bacteroidia,o:Bacteroidales,f:Bacteroidaceae,g:Phocaeicola,s:Phocaeicola vulgatus |
| OTU_405 | 0 | 0.5 | 10.5 | k:Bacteria,p:Firmicutes,c:Clostridia,o:Lachnospirales,f:Lachnospiraceae,g:uncultured |
| OTU_407 | 0 | 10.75 | 4 | k:Bacteria,p:Firmicutes,c:Clostridia,o:Lachnospirales,f:Lachnospiraceae,g:Lachnospiraceae NK4A136 |
| OTU_41 | 0 | 262.25 | 0 | k:Bacteria,p:Proteobacteria,c:Gammaproteobacteria,o:Pasteurellales,f:Pasteurellaceae |
| OTU_414 | 0 | 13.75 | 2 | k:Bacteria,p:Bacteroidota,c:Bacteroidia,o:Bacteroidales,f:Muribaculaceae |
| OTU_415 | 6 | 0 | 5.75 | k:Bacteria,p:Firmicutes,c:Bacilli,o:Erysipelotrichales,f:Erysipelotrichaceae |
| OTU_422 | 0 | 4 | 0 | k:Bacteria,p:Actinobacteriota,c:Coriobacteriia,o:Coriobacteriales,f:Eggerthellaceae,g:Enterorhabdus,s:uncultured |
| OTU_423 | 0 | 0 | 6.75 | k:Bacteria,p:Actinobacteriota,c:Coriobacteriia,o:Coriobacteriales,f:Eggerthellaceae,g:Enterorhabdus,s:uncultured |
| OTU_425 | 0 | 5.25 | 15 | k:Bacteria,p:Bacteroidota,c:Bacteroidia,o:Bacteroidales,f:Muribaculaceae |
| OTU_426 | 0 | 6.25 | 0 | k:Bacteria,p:Bacteroidota,c:Bacteroidia,o:Flavobacteriales,f:Weeksellaceae |
| OTU_427 | 0 | 8.75 | 0 | k:Bacteria,p:Bacteroidota,c:Bacteroidia,o:Bacteroidales,f:Muribaculaceae |
| OTU_430 | 0 | 0.25 | 6 | k:Bacteria,p:Bacteroidota,c:Bacteroidia,o:Bacteroidales,f:Muribaculaceae |
| OTU_43 | 0 | 124 | 0 | k:Bacteria,p:Campilobacterota,c:Campylobacteria,o:Campylobacterales,f:Helicobacteraceae,g:Helicobacter,s:Helicobacter cholecystus |
| OTU_431 | 0 | 1 | 7.75 | k:Bacteria,p:Firmicutes,c:Clostridia,o:Borkfalkiales,f:PAC001219,g:PAC001219 |
| OTU_434 | 0 | 0 | 6.5 | k:Bacteria,p:Chloroflexi,c:KD4-96 |
| OTU_437 | 0 | 3.75 | 2.25 | k:Bacteria,p:Bacteroidota,c:Bacteroidia,o:Bacteroidales,f:Tannerellaceae,g:Parabacteroides,s:uncultured |
| OTU_439 | 0 | 0 | 7 | k:Bacteria,p:Firmicutes,c:Clostridia,o:Clostridiales,f:Lachnospiraceae,g:Fusimonas,s:uncultured |
| OTU_44 | 0 | 3.75 | 191 | k:Bacteria,p:Firmicutes,c:Clostridia,o:Clostridiales,f:Lachnospiraceae,g:KE159571 |
| OTU_442 | 0 | 5.5 | 0 | k:Bacteria,p:Spirochaetota,c:Spirochaetia,o:Spirochaetales,f:Spirochaetaceae,g:Treponema,s:uncultured |
| OTU_443 | 0 | 3.25 | 4.75 | k:Bacteria,p:Firmicutes,c:Clostridia,o:Borkfalkiales,f:PAC001219,g:PAC001219 |
| OTU_446 | 0 | 4.75 | 0 | k:Bacteria,p:Actinobacteriota,c:Coriobacteriia,o:Coriobacteriales,f:Coriobacteriaceae,g:Collinsella,s:Collinsella intestinalis |
| OTU_447 | 0 | 9.25 | 0 | k:Bacteria,p:Bacteroidota,c:Bacteroidia,o:Bacteroidales,f:Prevotellaceae,g:Prevotella,s:uncultured |
| OTU_448 | 0 | 0 | 17 | k:Bacteria,p:Bacteroidota,c:Bacteroidia,o:Bacteroidales,f:Muribaculaceae |
| OTU_449 | 0 | 5.25 | 4 | k:Bacteria,p:Desulfobacterota,c:Desulfovibrionia,o:Desulfovibrionales,f:Desulfovibrionaceae,g:Desulfovibrio,s:uncultured |
| OTU_451 | 0 | 0 | 7.75 | k:Bacteria,p:Firmicutes,c:Clostridia,o:Lachnospirales,f:Lachnospiraceae,g:Lachnospiraceae NK4A136,s:uncultured |
| OTU_452 | 0.5 | 0 | 16 | k:Bacteria,p:Firmicutes,c:Clostridia,o:Clostridiales,f:Lachnospiraceae,g:PAC002368 |
| OTU_453 | 0 | 0 | 3.5 | k:Bacteria,p:Actinobacteriota,c:Actinobacteria,o:Micrococcales,f:Micrococcaceae |
| OTU_455 | 0 | 11 | 0 | k:Bacteria,p:Firmicutes,c:Negativicutes,o:Veillonellales-Selenomonadales,f:Veillonellaceae,g:Megasphaera,s:Megasphaera elsdenii |
| OTU_457 | 0 | 0 | 4.75 | k:Bacteria,p:Firmicutes,c:Clostridia,o:Borkfalkiales,f:PAC001219,g:JX095379 |
| OTU_459 | 0 | 3.75 | 14.25 | k:Bacteria,p:Firmicutes,c:Clostridia,o:Lachnospirales,f:Lachnospiraceae,g:GCA-900066575,s:uncultured |
| OTU_460 | 0 | 3.5 | 0 | k:Bacteria,p:Firmicutes,c:Clostridia,o:Oscillospirales,f:[Eubacterium] coprostanoligenes group |
| OTU_46 | 1.5 | 8.75 | 274.25 | k:Bacteria,p:Firmicutes,c:Clostridia,o:Lachnospirales,f:Lachnospiraceae,g:uncultured |
| OTU_462 | 0 | 0 | 12.75 | k:Bacteria,p:Firmicutes,c:Clostridia,o:Lachnospirales,f:Lachnospiraceae,g:Blautia,s:uncultured |
| OTU_463 | 0 | 2 | 6 | k:Bacteria,p:Firmicutes,c:Clostridia,o:Lachnospirales,f:Lachnospiraceae,g:uncultured |
| OTU_465 | 0 | 0 | 5.5 | k:Bacteria,p:Firmicutes,c:Clostridia,o:Borkfalkiales,f:Borkfalkiaceae,g:FJ848448 |
| OTU_467 | 0 | 8.25 | 27.5 | k:Bacteria,p:Firmicutes,c:Clostridia,o:Oscillospirales,f:Oscillospiraceae,g:uncultured |
| OTU_469 | 2.5 | 6 | 4.5 | k:Bacteria,p:Firmicutes,c:Clostridia,o:Clostridiales,f:Clostridiaceae,g:Clostridium sensu stricto 1,s:Clostridium perfringens |
| OTU_470 | 0 | 0 | 3 | k:Bacteria,p:Desulfobacterota,c:Desulfovibrionia,o:Desulfovibrionales,f:Desulfovibrionaceae,g:uncultured |
| OTU_471 | 0 | 11.5 | 9 | k:Bacteria,p:Bacteroidota,c:Bacteroidia,o:Bacteroidales,f:Rikenellaceae,g:Alistipes,s:uncultured |
| OTU_473 | 12.5 | 0.75 | 0 | k:Bacteria,p:Firmicutes,c:Clostridia,o:Peptostreptococcales-Tissierellales,f:Peptostreptococcaceae,g:Terrisporobacter,s:Terrisporobacter petrolearius |
| OTU_474 | 0 | 3.5 | 0.25 | k:Bacteria,p:Firmicutes,c:Clostridia,o:Clostridiales,f:Oscillospiraceae,g:Paludihabitans,s:uncultured |
| OTU_476 | 0 | 10 | 0 | k:Bacteria,p:Bacteroidota,c:Bacteroidia,o:Bacteroidales,f:Muribaculaceae |
| OTU_477 | 0 | 2 | 3 | k:Bacteria,p:Actinobacteriota,c:Coriobacteriia,o:Coriobacteriales,f:Eggerthellaceae,g:Enterorhabdus,s:uncultured |
| OTU_481 | 6 | 0.75 | 0 | k:Bacteria,p:Proteobacteria,c:Alphaproteobacteria,o:Paracaedibacterales,f:Paracaedibacteraceae,g:uncultured |
| OTU_484 | 0 | 8 | 0 | k:Bacteria,p:Bacteroidota,c:Bacteroidia,o:Bacteroidales,f:Rikenellaceae,g:Rikenellaceae RC9 gut group |
| OTU_485 | 0 | 0.25 | 7.75 | k:Bacteria,p:Firmicutes,c:Clostridia,o:Oscillospirales,f:Ruminococcaceae,g:Incertae Sedis,s:uncultured |
| OTU_488 | 0 | 1.25 | 15.75 | k:Bacteria,p:Bacteroidota,c:Bacteroidia,o:Bacteroidales,f:Muribaculaceae |
| OTU_489 | 0 | 7.25 | 0 | k:Bacteria,p:Actinobacteriota,c:Actinobacteria,o:Corynebacteriales,f:Corynebacteriaceae,g:Corynebacterium,s:Corynebacterium pseudotuberculosis |
| OTU_490 | 0 | 0.75 | 3 | k:Bacteria,p:Desulfobacterota,c:Desulfovibrionia,o:Desulfovibrionales,f:Desulfovibrionaceae,g:Desulfovibrio,s:uncultured |
| OTU_49 | 0.5 | 152 | 110 | k:Bacteria,p:Firmicutes,c:Clostridia,o:Lachnospirales,f:Lachnospiraceae,g:Lachnospiraceae NK4A136 group,s:uncultured |
| OTU_492 | 0 | 1 | 10.5 | k:Bacteria,p:Firmicutes,c:Clostridia,o:Peptococcales,f:Peptococcaceae,g:uncultured |
| OTU_493 | 1 | 3 | 10 | k:Bacteria,p:Proteobacteria,c:Gammaproteobacteria,o:Xanthomonadales,f:Xanthomonadaceae,g:Stenotrophomonas,s:Stenotrophomonas maltophilia |
| OTU_495 | 0 | 6.75 | 0 | k:Bacteria,p:Firmicutes,c:Bacilli,o:Bacillales,f:Planococcaceae,g:Sporosarcina |
| OTU_496 | 0 | 0 | 13.75 | k:Bacteria,p:Firmicutes,c:Clostridia,o:Clostridiales,f:Lachnospiraceae,g:PAC001116 |
| OTU_498 | 0 | 7 | 8 | k:Bacteria,p:Bacteroidota,c:Bacteroidia,o:Bacteroidales,f:Muribaculaceae |
| OTU_500 | 0 | 7.5 | 0 | k:Bacteria,p:Bacteroidota,c:Bacteroidia,o:Bacteroidales,f:Muribaculaceae |
| OTU_50 | 0 | 101 | 8.75 | k:Bacteria,p:Firmicutes,c:Clostridia,o:Clostridiales,f:Oscillospiraceae,g:Eubacterium,s:uncultured |
| OTU_503 | 0 | 3.75 | 0.5 | k:Bacteria,p:Desulfobacterota,c:Desulfovibrionia,o:Desulfovibrionales,f:Desulfovibrionaceae,g:Desulfovibrio,s:uncultured |
| OTU_506 | 0 | 0 | 9.75 | k:Bacteria,p:Firmicutes,c:Clostridia,o:Clostridia UCG-014 |
| OTU_507 | 0 | 9.25 | 12 | k:Bacteria,p:Bacteroidota,c:Bacteroidia,o:Bacteroidales,f:Muribaculaceae,g:PAC001062 |
| OTU_508 | 5.5 | 0 | 0 | k:Bacteria,p:Actinobacteriota,c:Actinobacteria,o:Actinomycetales,f:Actinomycetaceae,g:Actinomyces |
| OTU_510 | 0 | 5.75 | 0.5 | k:Bacteria,p:Bacteroidota,c:Bacteroidia,o:Bacteroidales,f:Muribaculaceae |
| OTU_51 | 6.5 | 316.75 | 0 | k:Bacteria,p:Proteobacteria,c:Gammaproteobacteria,o:Pasteurellales,f:Pasteurellaceae,g:Rodentibacter |
| OTU_511 | 0 | 2.75 | 2 | k:Bacteria,p:Bacteroidota,c:Bacteroidia,o:Flavobacteriales,f:Flavobacteriaceae,g:uncultured |
| OTU_512 | 0 | 11.25 | 0 | k:Bacteria,p:Proteobacteria,c:Gammaproteobacteria,o:Burkholderiales,f:Sutterellaceae,g:Parasutterella |
| OTU_513 | 0 | 0 | 7 | k:Bacteria,p:Firmicutes,c:Clostridia,o:Borkfalkiales,f:Borkfalkiaceae,g:PAC001141 |
| OTU_514 | 0 | 0 | 7.5 | k:Bacteria,p:Firmicutes,c:Clostridia,o:Borkfalkiales,f:Borkfalkiaceae,g:PAC001441 |
| OTU_515 | 0 | 2 | 5.25 | k:Bacteria,p:Bacteroidota,c:Bacteroidia,o:Bacteroidales,f:Muribaculaceae,g:RIAY |
| OTU_517 | 0 | 1 | 4.25 | k:Bacteria,p:Firmicutes,c:Clostridia,o:Oscillospirales,f:UCG-010 |
| OTU_519 | 8 | 2.5 | 3.25 | k:Bacteria,p:Proteobacteria,c:Gammaproteobacteria,o:Enterobacterales,f:Hafniaceae,g:Hafnia,s:Hafnia alvei |
| OTU_520 | 0 | 0.75 | 3 | k:Bacteria,p:Verrucomicrobiota,c:Chlamydiae,o:Chlamydiales.f:EU488135,g:EU488135,s:uncultured |
| OTU_521 | 0 | 0 | 19.5 | k:Bacteria,p:Firmicutes,c:Clostridia,o:Clostridiales,f:Oscillospiraceae,g:PAC000661,s:uncultured |
| OTU_52 | 26 | 39.25 | 92.25 | k:Bacteria,p:Firmicutes,c:Clostridia,o:Peptostreptococcales,f:Peptostreptococcaceae,g:Paraclostridium,s:Paraclostridium benzoelyticum |
| OTU_523 | 0 | 4.5 | 3.75 | k:Bacteria,p:Actinobacteriota,c:Coriobacteriia,o:Coriobacteriales,f:Eggerthellaceae,g:Adlercreutzia |
| OTU_524 | 0 | 8.75 | 0 | k:Bacteria,p:Firmicutes,c:Bacilli,o:Staphylococcales,f:Staphylococcaceae,g:Macrococcus |
| OTU_526 | 0 | 1 | 6.5 | k:Bacteria,p:Firmicutes,c:Clostridia,o:Oscillospirales,f:Oscillospiraceae,g:uncultured |
| OTU_527 | 0 | 3.75 | 0 | k:Bacteria,p:Proteobacteria,c:Gammaproteobacteria,o:Burkholderiales,f:Neisseriaceae,g:Uruburuella,s:Uruburuella suis |
| OTU_528 | 0 | 8.5 | 0 | k:Bacteria,p:Firmicutes,c:Clostridia,o:Lachnospirales,f:Lachnospiraceae,g:Blautia,s:Blautia hansenii |
| OTU_529 | 0 | 0 | 6.75 | k:Bacteria,p:Bacteroidota,c:Bacteroidia,o:Bacteroidales,f:Rikenellaceae,g:Alistipes,s:uncultured |
| OTU_53 | 0 | 100.25 | 23 | k:Bacteria,p:Firmicutes,c:Clostridia,o:Lachnospirales,f:Lachnospiraceae,g:uncultured |
| OTU_532 | 0 | 1.5 | 11 | k:Bacteria,p:Firmicutes,c:Clostridia,o:Clostridia UCG-014 |
| OTU_533 | 0 | 0 | 11.25 | k:Bacteria,p:Bacteroidota,c:Bacteroidia,o:Bacteroidales,f:Muribaculaceae |
| OTU_535 | 0 | 2.75 | 5.25 | k:Bacteria,p:Firmicutes,c:Clostridia,o:Oscillospirales,f:Oscillospiraceae,g:uncultured |
| OTU_539 | 0 | 0.25 | 16.5 | k:Bacteria,p:Bacteroidota,c:Bacteroidia,o:Bacteroidales,f:Muribaculaceae |
| OTU_540 | 0 | 0 | 5 | k:Bacteria,p:Proteobacteria,c:Alphaproteobacteria,o:Azospirillales,f:Azospirillaceae,g:Azospirillum,s:Azospirillum fermentarium |
| OTU_541 | 0 | 0 | 6 | k:Bacteria,p:Firmicutes,c:Bacilli,o:RF39 |
| OTU_545 | 0 | 1.25 | 14.5 | k:Bacteria,p:Firmicutes,c:Clostridia,o:Oscillospirales,f:Ruminococcaceae,g:Harryflintia,s:uncultured |
| OTU_55 | 0 | 252.75 | 1 | k:Bacteria,p:Actinobacteriota,c:Actinobacteria,o:Bifidobacteriales,f:Bifidobacteriaceae,g:Bifidobacterium |
| OTU_551 | 2 | 6.5 | 2 | k:Bacteria,p:Actinobacteriota,c:Actinobacteria,o:Actinomycetales,f:Actinomycetaceae,g:Actinomyces |
| OTU_557 | 0 | 11.25 | 4.25 | k:Bacteria,p:Bacteroidota,c:Bacteroidia,o:Bacteroidales,f:Muribaculaceae |
| OTU_560 | 0 | 0.5 | 11.75 | k:Bacteria,p:Firmicutes,c:Clostridia,o:Oscillospirales,f:Ruminococcaceae |
| OTU_56 | 0 | 1 | 171 | k:Bacteria,p:Bacteroidota,c:Bacteroidia,o:Bacteroidales,f:Bacteroidaceae,g:Phocaeicola,s:Phocaeicola sartorii |
| OTU_564 | 0 | 0 | 4.25 | k:Bacteria,p:Firmicutes,c:Clostridia,o:Oscillospirales,f:[Eubacterium] coprostanoligenes group |
| OTU_566 | 0 | 0 | 2.5 | k:Bacteria,p:Firmicutes,c:Clostridia,o:Oscillospirales,f:Oscillospiraceae |
| OTU_567 | 0 | 0 | 4.5 | k:Bacteria,p:Firmicutes,c:Clostridia,o:Oscillospirales,f:Ruminococcaceae,g:uncultured |
| OTU_57 | 0 | 146.5 | 2.25 | k:Bacteria,p:Desulfobacterota,c:Desulfovibrionia,o:Desulfovibrionales,f:Desulfovibrionaceae,g:Desulfovibrio,s:uncultured |
| OTU_570 | 26.5 | 17.75 | 33.5 | k:Bacteria,p:Firmicutes,c:Clostridia,o:Peptostreptococcales-Tissierellales,f:Peptostreptococcaceae,g:Paeniclostridium,s:Eubacterium tenue |
| OTU_571 | 0 | 0.25 | 6.5 | k:Bacteria,p:Firmicutes,c:Clostridia,o:Lachnospirales,f:Lachnospiraceae,g:uncultured |
| OTU_573 | 0 | 6.25 | 1.25 | k:Bacteria,p:Firmicutes,c:Clostridia,o:Peptococcales,f:Peptococcaceae,g:uncultured |
| OTU_577 | 0 | 4.5 | 0 | k:Bacteria,p:Fusobacteriota,c:Fusobacteriia,o:Fusobacteriales,f:Fusobacteriaceae,g:Fusobacterium,s:uncultured |
| OTU_58 | 0 | 0 | 103.25 | k:Bacteria,p:Bacteroidota,c:Bacteroidia,o:Bacteroidales,f:Rikenellaceae,g:Alistipes,s:uncultured |
| OTU_583 | 0 | 2.5 | 0.25 | k:Bacteria,p:Firmicutes,c:Clostridia,o:Lachnospirales,f:Lachnospiraceae,g:ASF356 |
| OTU_584 | 0 | 0 | 4.75 | k:Bacteria,p:Proteobacteria,c:Alphaproteobacteria,o:Rhizobiales,f:Beijerinckiaceae,g:Bosea |
| OTU_586 | 0 | 4.25 | 6.25 | k:Bacteria,p:Bacteroidota,c:Bacteroidia,o:Bacteroidales,f:Muribaculaceae |
| OTU_588 | 0 | 0.5 | 5.5 | k:Bacteria,p:Bacteroidota,c:Bacteroidia,o:Bacteroidales,f:Rikenellaceae,g:Rikenellaceae RC9 gut group,s:uncultured |
| OTU_590 | 1.5 | 2.5 | 3.25 | k:Bacteria,p:Firmicutes,c:Bacilli,o:Lactobacillales,f:Streptococcaceae,g:Lactococcus,s:Lactococcus garvieae |
| OTU_591 | 0 | 2.5 | 4 | k:Bacteria,p:Bacteroidota,c:Bacteroidia,o:Bacteroidales,f:Muribaculaceae |
| OTU_594 | 0 | 1.75 | 0 | k:Bacteria,p:Patescibacteria,c:Saccharimonadia,o:Saccharimonadales |
| OTU_595 | 0 | 10.5 | 0 | k:Bacteria,p:Bacteroidota,c:Bacteroidia,o:Bacteroidales,f:Tannerellaceae,g:Parabacteroides,s:Parabacteroides merdae |
| OTU_600 | 0 | 0 | 4.25 | k:Bacteria,p:Firmicutes,c:Clostridia,o:Clostridiales,f:Lachnospiraceae,g:PAC001408 |
| OTU_601 | 0 | 0 | 4.75 | k:Bacteria,p:Firmicutes,c:Clostridia,o:uncultured |
| OTU_603 | 0 | 3.5 | 0 | k:Bacteria,p:Proteobacteria,c:Gammaproteobacteria,o:Pseudomonadales,f:Pseudomonadaceae,g:Pseudomonas,s:Pseudomonas palmensis |
| OTU_606 | 0 | 9.5 | 0 | k:Bacteria,p:Bacteroidota,c:Bacteroidia,o:Bacteroidales,f:Muribaculaceae |
| OTU_612 | 0 | 3.25 | 0 | k:Bacteria,p:Bacteroidota,c:Bacteroidia,o:Bacteroidales,f:Porphyromonadaceae,g:Porphyromonas,s:Porphyromonas cangingivalis |
| OTU_613 | 0 | 2.5 | 15.75 | k:Bacteria,p:Firmicutes,c:Clostridia,o:Clostridiales,f:Lachnospiraceae,g:Enterocloster,s:uncultured |
| OTU_614 | 0 | 0 | 8.5 | k:Bacteria,p:Firmicutes,c:Clostridia,o:Borkfalkiales,f:PAC001219,g:PAC001219 |
| OTU_615 | 0 | 6.5 | 1 | k:Bacteria,p:Firmicutes,c:Clostridia,o:Clostridiales,f:Lachnospiraceae,g:AF287775 |
| OTU_616 | 0 | 8 | 10.25 | k:Bacteria,p:Bacteroidota,c:Bacteroidia,o:Bacteroidales,f:Tannerellaceae,g:Parabacteroides,s:uncultured |
| OTU_618 | 0 | 1.25 | 2.25 | k:Bacteria,p:Firmicutes,c:Clostridia,o:Clostridiales,f:Lachnospiraceae,g:Eisenbergiella |
| OTU_622 | 0 | 6.5 | 3.75 | k:Bacteria,p:Firmicutes,c:Clostridia,o:Lachnospirales,f:Lachnospiraceae,g:uncultured |
| OTU_623 | 0 | 3.25 | 1.5 | k:Bacteria,p:Firmicutes,c:Clostridia,o:Lachnospirales,f:Lachnospiraceae,g:Roseburia,s:uncultured |
| OTU_6 | 1570.5 | 668.25 | 5854 | k:Bacteria,p:Proteobacteria,c:Gammaproteobacteria,o:Burkholderiales,f:Burkholderiaceae,g:Ralstonia,s:Ralstonia insidiosa |
| OTU_626 | 0 | 0 | 7.25 | k:Bacteria,p:Bacteroidota,c:Bacteroidia,o:Bacteroidales,f:Muribaculaceae |
| OTU_628 | 0 | 0 | 12.5 | k:Bacteria,p:Firmicutes,c:Clostridia,o:Lachnospirales,f:Lachnospiraceae,g:Roseburia,s:uncultured |
| OTU_629 | 0 | 0 | 2.25 | k:Bacteria,p:Proteobacteria,c:Alphaproteobacteria,o:Rhizobiales,f:Xanthobacteraceae |
| OTU_630 | 0.5 | 0 | 7 | k:Bacteria,p:Firmicutes,c:Bacilli,o:Bacillales,f:Bacillaceae,g:Halalkalibacterium,s:Halalkalibacterium halodurans |
| OTU_636 | 0 | 0 | 6.25 | k:Bacteria,p:Bacteroidota,c:Bacteroidia,o:Bacteroidales,f:Tannerellaceae,g:Parabacteroides,s:uncultured |
| OTU_63 | 301.5 | 41.5 | 2.75 | k:Bacteria,p:Proteobacteria,c:Gammaproteobacteria,o:Enterobacterales,f:Enterobacteriaceae,g:Citrobacter,s:Citrobacter koseri |
| OTU_638 | 0 | 0.25 | 3.5 | k:Bacteria,p:Firmicutes,c:Clostridia,o:Oscillospirales,f:Oscillospiraceae |
| OTU_639 | 0 | 0 | 2.25 | k:Bacteria,p:Firmicutes,c:Clostridia,o:Oscillospirales,f:Ruminococcaceae,g:uncultured |
| OTU_640 | 0 | 0 | 2.25 | k:Bacteria,p:Proteobacteria,c:Alphaproteobacteria,o:Rhodobacterales,f:Rhodobacteraceae,g:Rubellimicrobium |
| OTU_64 | 0 | 0 | 133.75 | k:Bacteria,p:Bacteroidota,c:Bacteroidia,o:Bacteroidales,f:F082 |
| OTU_641 | 0 | 0 | 6.25 | k:Bacteria,p:Firmicutes,c:Clostridia,o:Clostridiales,f:Lachnospiraceae,g:Hominiventricola,s:uncultured |
| OTU_642 | 0 | 0 | 6 | k:Bacteria,p:Firmicutes,c:Bacilli,o:RF39 |
| OTU_65 | 0.5 | 2.5 | 219.5 | k:Bacteria,p:Firmicutes,c:Clostridia,o:Borkfalkiales,f:Borkfalkiaceae,g:PAC001371 |
| OTU_660 | 0 | 2 | 4.25 | k:Bacteria,p:Firmicutes,c:Clostridia,o:Clostridiales,f:Lachnospiraceae,g:PAC001092 |
| OTU_661 | 0 | 14.75 | 0 | k:Bacteria,p:Bacteroidota,c:Bacteroidia,o:Bacteroidales,f:Muribaculaceae |
| OTU_66 | 0.5 | 146.75 | 13 | k:Bacteria,p:Firmicutes,c:Clostridia,o:Clostridiales,f:Lachnospiraceae,g:PAC001516 |
| OTU_662 | 0 | 0 | 6.5 | k:Bacteria,p:Bacteroidota,c:Bacteroidia,o:Bacteroidales,f:Muribaculaceae |
| OTU_667 | 0 | 3 | 0 | k:Bacteria,p:Bacteroidota,c:Bacteroidia,o:Bacteroidales,f:Muribaculaceae |
| OTU_669 | 0 | 2.5 | 0 | k:Bacteria,p:Firmicutes,c:Negativicutes,o:Veillonellales-Selenomonadales,f:Selenomonadaceae,g:Mitsuokella |
| OTU_670 | 0 | 2 | 0 | k:Bacteria,p:Cyanobacteria,c:Cyanobacteriia,o:Cyanobacteriales,f:Chroococcidiopsaceae,g:Aliterella |
| OTU_671 | 0 | 1.75 | 0 | k:Bacteria,p:Proteobacteria,c:Alphaproteobacteria,o:Rhodobacterales,f:Rhodobacteraceae |
| OTU_672 | 0 | 1.25 | 3.75 | k:Bacteria,p:Firmicutes,c:Clostridia,o:Oscillospirales,f:[Eubacterium] coprostanoligenes group |
| OTU_676 | 0 | 2 | 8 | k:Bacteria,p:Firmicutes,c:Clostridia,o:Lachnospirales,f:Lachnospiraceae,g:uncultured |
| OTU_677 | 0 | 0 | 9.5 | k:Bacteria,p:Firmicutes,c:Clostridia,o:Borkfalkiales,f:Borkfalkiaceae,g:PAC002169 |
| OTU_678 | 0 | 2.25 | 0 | k:Bacteria,p:Firmicutes,c:Bacilli,o:Erysipelotrichales,f:Erysipelotrichaceae,g:Dubosiella,s:uncultured |
| OTU_679 | 0 | 2.25 | 1.75 | k:Bacteria,p:Actinobacteriota,c:Actinobacteria,o:Corynebacteriales,f:Corynebacteriaceae,g:Lawsonella,s:Lawsonella clevelandensis |
| OTU_680 | 0 | 0 | 2.25 | k:Bacteria,p:Proteobacteria,c:Alphaproteobacteria,o:Rhodobacterales,f:Rhodobacteraceae,g:Gemmobacter |
| OTU_683 | 0 | 5.75 | 2 | k:Bacteria,p:Firmicutes,c:Clostridia,o:Oscillospirales,f:Ruminococcaceae,g:uncultured |
| OTU_686 | 0 | 0 | 11.5 | k:Bacteria,p:Firmicutes,c:Clostridia,o:Clostridia UCG-014 |
| OTU_689 | 0 | 0 | 1.75 | k:Bacteria,p:Firmicutes,c:Clostridia,o:Lachnospirales,f:Lachnospiraceae,g:uncultured |
| OTU_69 | 0 | 0 | 142.5 | k:Bacteria,p:Bacteroidota,c:Bacteroidia,o:Bacteroidales,f:Prevotellaceae,g:Alloprevotella,s:uncultured |
| OTU_690 | 3.5 | 8.5 | 99 | k:Bacteria,p:Firmicutes,c:Bacilli,o:Lactobacillales,f:Lactobacillaceae,g:Lactobacillus,s:uncultured |
| OTU_692 | 0 | 6 | 0 | k:Bacteria,p:Actinobacteriota,c:Actinobacteria,o:Micrococcales,f:Micrococcaceae,g:Rothia |
| OTU_694 | 0 | 0.5 | 4.75 | k:Bacteria,p:Firmicutes,c:Clostridia,o:Lachnospirales,f:Lachnospiraceae,g:uncultured |
| OTU_696 | 0 | 2.5 | 0.25 | k:Bacteria,p:Firmicutes,c:Clostridia,o:Lachnospirales,f:Lachnospiraceae,g:uncultured |
| OTU_697 | 0 | 0 | 4 | k:Bacteria,p:Bacteroidota,c:Bacteroidia,o:Bacteroidales,f:Muribaculaceae |
| OTU_699 | 0 | 0.25 | 2 | k:Bacteria,p:Firmicutes,c:Clostridia,o:uncultured |
| OTU_700 | 0 | 0.5 | 2.5 | k:Bacteria,p:Firmicutes,c:Clostridia,o:Oscillospirales,f:Ruminococcaceae |
| OTU_701 | 0 | 0 | 2.5 | k:Bacteria,p:Actinobacteriota,c:Actinobacteria,o:Micrococcales,f:Micrococcaceae,g:Nesterenkonia |
| OTU_702 | 6.5 | 42.5 | 0 | k:Bacteria,p:Proteobacteria,c:Gammaproteobacteria,o:Pasteurellales,f:Pasteurellaceae,g:Rodentibacter |
| OTU_705 | 0 | 2 | 0 | k:Bacteria,p:Bacteroidota,c:Bacteroidia,o:Bacteroidales,f:Muribaculaceae |
| OTU_708 | 0 | 16.5 | 0 | k:Bacteria,p:Firmicutes,c:Bacilli,o:Erysipelotrichales,f:Erysipelotrichaceae,g:Turicibacter,s:Turicibacter sp. LA61 |
| OTU_712 | 0 | 1.25 | 1.5 | k:Bacteria,p:Firmicutes,c:Clostridia,o:Monoglobales,f:Monoglobaceae,g:Monoglobus |
| OTU_714 | 5 | 0 | 0 | k:Bacteria,p:Actinobacteriota,c:Actinobacteria,o:Propionibacteriales,f:Propionibacteriaceae,g:Cutibacterium |
| OTU_715 | 0 | 3.5 | 0 | k:Bacteria,p:Firmicutes,c:Clostridia,o:Oscillospirales,f:UCG-010 |
| OTU_719 | 0 | 0 | 3 | k:Bacteria,p:Actinobacteriota,c:Actinobacteria,o:Micromonosporales,f:Micromonosporaceae,g:Stackebrandtia,s:uncultured |
| OTU_72 | 0 | 0.25 | 180.5 | k:Bacteria,p:Bacteroidota,c:Bacteroidia,o:Bacteroidales,f:Marinifilaceae,g:Odoribacter,s:uncultured |
| OTU_720 | 0 | 2.25 | 0 | k:Bacteria,p:Firmicutes,c:Clostridia,o:Clostridiales,f:Lachnospiraceae,g:PAC001124 |
| OTU_721 | 0 | 7.5 | 0 | k:Bacteria,p:Firmicutes,c:Clostridia,o:Lachnospirales,f:Lachnospiraceae,g:Tyzzerella,s:uncultured |
| OTU_723 | 0 | 10.5 | 0 | k:Bacteria,p:Actinobacteriota,c:Actinobacteria,o:Micrococcales,f:Intrasporangiaceae |
| OTU_73 | 0 | 67 | 4.25 | k:Bacteria,p:Firmicutes,c:Clostridia,o:Lachnospirales,f:Lachnospiraceae,g:uncultured |
| OTU_732 | 0 | 2.25 | 0 | k:Bacteria,p:Actinobacteriota,c:Coriobacteriia,o:Coriobacteriales,f:Atopobiaceae,g:Coriobacteriaceae-UCG-002,s:Leptogranulimonas caecicola |
| OTU_734 | 0 | 0.75 | 4 | k:Bacteria,p:Firmicutes,c:Clostridia,o:Clostridiales,f:Lachnospiraceae,g:PAC001563 |
| OTU_735 | 0 | 0 | 5.75 | k:Bacteria,p:Firmicutes,c:Clostridia,o:Borkfalkiales,f:Borkfalkiaceae,g:PAC001371 |
| OTU_738 | 0 | 5.25 | 0 | k:Bacteria,p:Bacteroidota,c:Bacteroidia,o:Bacteroidales,f:Muribaculaceae |
| OTU_7 | 2825 | 2536.75 | 3716.75 | k:Bacteria,p:Firmicutes,c:Bacilli,o:Lactobacillales,f:Lactobacillaceae,g:Lactobacillus,s:Lactobacillus johnsonii |
| OTU_74 | 0 | 3 | 150.75 | k:Bacteria,p:Bacteroidota,c:Bacteroidia,o:Bacteroidales,f:Rs-E47 termite group |
| OTU_745 | 0 | 4.25 | 1 | k:Bacteria,p:Firmicutes,c:Clostridia,o:Oscillospirales,f:Ruminococcaceae,g:Candidatus Soleaferrea,s:uncultured |
| OTU_748 | 9.5 | 0 | 0 | k:Bacteria,p:Bacteroidota,c:Chitinophagia,o:Chitinophagales,f:Chitinophagaceae,g:Segetibacter,s:uncultured |
| OTU_751 | 0 | 2.5 | 1.5 | k:Bacteria,p:Bacteroidota,c:Bacteroidia,o:Bacteroidales,f:Muribaculaceae |
| OTU_757 | 0 | 1.75 | 0 | k:Bacteria,p:Desulfobacterota,c:Desulfovibrionia,o:Desulfovibrionales,f:Desulfovibrionaceae,g:Desulfovibrio,s:Desulfovibrio porci |
| OTU_761 | 2.5 | 0 | 0 | k:Bacteria,p:Proteobacteria,c:Gammaproteobacteria,o:Cardiobacteriales,f:Cardiobacteriaceae |
| OTU_768 | 0.5 | 2 | 1.25 | k:Bacteria,p:Firmicutes,c:Clostridia,o:Clostridiales,f:Clostridiaceae,g:Clostridium,s:Clostridium colicanis |
| OTU_77 | 0 | 12.25 | 188.25 | k:Bacteria,p:Bacteroidota,c:Bacteroidia,o:Bacteroidales,f:Muribaculaceae |
| OTU_772 | 6 | 0 | 0 | k:Bacteria,p:Firmicutes,c:Bacilli,o:Lactobacillales,f:Lactobacillaceae,g:Lactobacillus,s:Lacticaseibacillus mingshuiensis |
| OTU_773 | 0 | 2 | 0 | k:Bacteria,p:Firmicutes,c:Bacilli,o:Erysipelotrichales,f:Erysipelotrichaceae,g:uncultured |
| OTU_778 | 0 | 3 | 0.75 | k:Bacteria,p:Proteobacteria,c:Alphaproteobacteria,o:Rhizobiales,f:Beijerinckiaceae,g:Methylobacterium,s:Methylobacterium gossipiicola |
| OTU_78 | 0 | 119.5 | 0.5 | k:Bacteria,p:Firmicutes,c:Bacilli,o:Bacillales,f:Planococcaceae,g:Lysinibacillus |
| OTU_787 | 6 | 0 | 0 | k:Bacteria,p:Bacteroidota,c:Bacteroidia,o:Bacteroidales,f:Porphyromonadaceae,g:Porphyromonas |
| OTU_79 | 0 | 170.5 | 1.25 | k:Bacteria,p:Firmicutes,c:Bacilli,o:Lactobacillales,f:Streptococcaceae,g:Streptococcus,s:Peptostreptococcus anaerobius |
| OTU_793 | 0 | 1 | 0.75 | k:Bacteria,p:Firmicutes,c:Clostridia,o:Oscillospirales,f:[Eubacterium] coprostanoligenes group |
| OTU_796 | 5 | 0 | 0 | k:Bacteria,p:Proteobacteria,c:Deltaproteobacteria,o:PAC001922 |
| OTU_801 | 0 | 0.25 | 3.75 | k:Bacteria,p:Firmicutes,c:Clostridia,o:Lachnospirales,f:Lachnospiraceae,g:uncultured |
| OTU_802 | 0 | 2 | 0 | k:Bacteria,p:Proteobacteria,c:Gammaproteobacteria,o:Burkholderiales,f:Comamonadaceae,g:Comamonas,s:uncultured |
| OTU_805 | 3 | 0 | 0 | k:Bacteria,p:Actinobacteriota,c:Actinobacteria,o:Corynebacteriales,f:Corynebacteriaceae,g:Corynebacterium,s:Corynebacterium camporealensis |
| OTU_806 | 0 | 0.75 | 5.75 | k:Bacteria,p:Firmicutes,c:Clostridia,o:Clostridiales,f:Lachnospiraceae,g:PAC001588 |
| OTU_807 | 0 | 3.75 | 0 | k:Bacteria,p:Bacteroidota,c:Bacteroidia,o:Bacteroidales,f:Rikenellaceae,g:Alistipes,s:uncultured |
| OTU_810 | 0 | 3.25 | 1 | k:Bacteria,p:Firmicutes,c:Clostridia,o:Clostridiales,f:Oscillospiraceae,g:PAC002409 |
| OTU_81 | 0 | 0.75 | 140 | k:Bacteria,p:Firmicutes,c:Clostridia,o:Clostridiales,f:Lachnospiraceae,g:PAC001166 |
| OTU_811 | 0 | 0 | 8 | k:Bacteria,p:Firmicutes,c:Clostridia,o:Oscillospirales,f:Oscillospiraceae,g:NK4A214 group |
| OTU_812 | 0 | 4.75 | 0 | k:Bacteria,p:Desulfobacterota,c:Desulfovibrionia,o:Desulfovibrionales,f:Desulfovibrionaceae,g:Desulfovibrio,s:uncultured |
| OTU_814 | 0 | 0 | 6.5 | k:Bacteria,p:Bacteroidota,c:Bacteroidia,o:Bacteroidales,f:Muribaculaceae |
| OTU_816 | 0 | 1.25 | 2.25 | k:Bacteria,p:Bacteroidota,c:Bacteroidia,o:Bacteroidales,f:Rikenellaceae,g:Rikenella |
| OTU_817 | 0 | 0.25 | 5.5 | k:Bacteria,p:Firmicutes,c:Clostridia,o:Lachnospirales,f:Lachnospiraceae,g:uncultured |
| OTU_818 | 0 | 2.25 | 7.25 | k:Bacteria,p:Firmicutes,c:Clostridia,o:Lachnospirales,f:Lachnospiraceae,g:uncultured |
| OTU_82 | 0.5 | 34 | 133.75 | k:Bacteria,p:Firmicutes,c:Clostridia,o:Lachnospirales,f:Lachnospiraceae,g:Lachnospiraceae NK4A136 group |
| OTU_825 | 0 | 0 | 4.25 | k:Bacteria,p:Firmicutes,c:Clostridia,o:Lachnospirales,f:Lachnospiraceae,g:Lachnospiraceae NK4A136 group,s:uncultured |
| OTU_826 | 0 | 2.5 | 0 | k:Bacteria,p:Firmicutes,c:Bacilli,o:Lactobacillales,f:Lactobacillaceae,g:Lactobacillus,s:Ligilactobacillus ruminis |
| OTU_827 | 0 | 0 | 3.25 | k:Bacteria,p:Actinobacteriota,c:Coriobacteriia,o:Coriobacteriales,f:Eggerthellaceae,g:Enterorhabdus,s:uncultured |
| OTU_8 | 14230.5 | 240.75 | 1445.5 | k:Bacteria,p:Firmicutes,c:Bacilli,o:Lactobacillales,f:Lactobacillaceae,g:Lactobacillus,s:Limosilactobacillus |
| OTU_829 | 0 | 1 | 0.75 | k:Bacteria,p:Firmicutes,c:Clostridia,o:Lachnospirales,f:Lachnospiraceae,g:Roseburia |
| OTU_830 | 0 | 1.25 | 1.5 | k:Bacteria,p:Firmicutes,c:Clostridia,o:Lachnospirales,f:Lachnospiraceae,g:Eubacterium,s:uncultured |
| OTU_831 | 0 | 6.5 | 0 | k:Bacteria,p:Bacteroidota,c:Bacteroidia,o:Bacteroidales,f:Muribaculaceae |
| OTU_83 | 0.5 | 43.5 | 33.5 | k:Bacteria,p:Firmicutes,c:Bacilli,o:Lactobacillales,f:Aerococcaceae,g:Globicatella,s:uncultured |
| OTU_832 | 0 | 0.25 | 3.25 | k:Bacteria,p:Bacteroidota,c:Bacteroidia,o:Bacteroidales,f:Muribaculaceae |
| OTU_833 | 0 | 1.75 | 0 | k:Bacteria,p:Proteobacteria,c:Gammaproteobacteria,o:Burkholderiales,f:Alcaligenaceae,g:Alcaligenes,s:Alcaligenes aquatilis |
| OTU_834 | 0 | 0.5 | 1 | k:Bacteria,p:Actinobacteriota,c:Coriobacteriia,o:Coriobacteriales,f:Eggerthellaceae |
| OTU_835 | 0 | 3.25 | 1.75 | k:Bacteria,p:Patescibacteria,c:Saccharimonadia,o:Saccharimonadales |
| OTU_839 | 5 | 0.25 | 0 | k:Bacteria,p:Proteobacteria,c:Alphaproteobacteria,o:Rhizobiales,f:Devosiaceae |
| OTU_84 | 0 | 139 | 0 | k:Bacteria,p:Bacteroidota,c:Bacteroidia,o:Bacteroidales,f:Porphyromonadaceae,g:Porphyromonas,s:uncultured |
| OTU_841 | 0 | 3 | 0 | k:Bacteria,p:Firmicutes,c:Clostridia,o:Lachnospirales,f:Lachnospiraceae,g:Oribacterium,s:uncultured |
| OTU_851 | 12 | 0 | 0.75 | k:Bacteria,p:Firmicutes,c:Bacilli,o:Lactobacillales,f:Lactobacillaceae,g:Lactobacillus,s:Lacticaseibacillus zeae |
| OTU_852 | 0 | 0.5 | 3.25 | k:Bacteria,p:Firmicutes,c:Clostridia,o:Clostridiales,f:Lachnospiraceae,g:Frisingicoccus,s:uncultured |
| OTU_853 | 0 | 6.25 | 0 | k:Bacteria,p:Firmicutes,c:Clostridia,o:Oscillospirales,f:Oscillospiraceae,g:UCG-005,s:uncultured |
| OTU_856 | 0 | 2.75 | 1 | k:Bacteria,p:Actinobacteriota,c:Coriobacteriia,o:Coriobacteriales,f:Eggerthellaceae,g:DNF00809,s:uncultured |
| OTU_86 | 0 | 43.25 | 62 | k:Bacteria,p:Bacteroidota,c:Bacteroidia,o:Bacteroidales,f:Marinifilaceae,g:Odoribacter |
| OTU_861 | 0 | 6 | 0 | k:Bacteria,p:Firmicutes,c:Bacilli,o:Erysipelotrichales,f:Erysipelotrichaceae,g:Allobaculum |
| OTU_867 | 0 | 0 | 1.75 | k:Bacteria,p:Fusobacteriota,c:Fusobacteriia,o:Fusobacteriales,f:Fusobacteriaceae,g:Cetobacterium |
| OTU_868 | 0 | 4.75 | 2 | k:Bacteria,p:Bacteroidota,c:Bacteroidia,o:Bacteroidales,f:Muribaculaceae |
| OTU_87 | 0.5 | 2.75 | 89.25 | k:Bacteria,p:Firmicutes,c:Clostridia,o:Borkfalkiales,f:PAC001219,g:PAC001219 |
| OTU_872 | 4.5 | 0 | 0 | k:Bacteria,p:Firmicutes,c:Bacilli,o:Lactobacillales,f:Lactobacillaceae,g:Lactobacillus,s:Lacticaseibacillus porcinae |
| OTU_873 | 0 | 1 | 0 | k:Bacteria,p:Proteobacteria,c:Gammaproteobacteria,o:Xanthomonadales,f:Xanthomonadaceae,g:Thermomonas |
| OTU_874 | 0 | 2.25 | 2.25 | k:Bacteria,p:Bacteroidota,c:Bacteroidia,o:Bacteroidales,f:Muribaculaceae |
| OTU_878 | 0 | 0 | 1.5 | k:Bacteria,p:Proteobacteria,c:Alphaproteobacteria,o:Ferrovibrionales,g:Taonella,s:Taonella mepensis |
| OTU_879 | 0 | 1.75 | 0.25 | k:Bacteria,p:Firmicutes,c:Clostridia,o:Oscillospirales,f:Ruminococcaceae,g:Pygmaiobacter,s:uncultured |
| OTU_881 | 0 | 1.25 | 1.75 | k:Bacteria,p:Firmicutes,c:Clostridia,o:Oscillospirales,f:Ruminococcaceae,g:Harryflintia,s:uncultured |
| OTU_882 | 0 | 1.5 | 0 | k:Bacteria,p:Bacteroidota,c:Bacteroidia,o:Bacteroidales,f:Muribaculaceae |
| OTU_884 | 0 | 0 | 3 | k:Bacteria,p:Actinobacteriota,c:Actinomycetia,o:Micrococcales,f:Micrococcaceae,g:Pseudoglutamicibacter,s:Pseudoglutamicibacter cumminsii |
| OTU_889 | 0 | 0 | 5.75 | k:Bacteria,p:Bacteroidota,c:Bacteroidia,o:Bacteroidales,f:Muribaculaceae |
| OTU_890 | 0 | 0.75 | 0.75 | k:Bacteria,p:Actinobacteriota,c:Coriobacteriia,o:Coriobacteriales,f:Coriobacteriales Incertae Sedis,g:uncultured |
| OTU_89 | 0 | 1.25 | 103.75 | k:Bacteria,p:Firmicutes,c:Clostridia,o:Lachnospirales,f:Lachnospiraceae,g:uncultured |
| OTU_892 | 0 | 2.5 | 0.75 | k:Bacteria,p:Firmicutes,c:Clostridia,o:Oscillospirales,f:Ruminococcaceae,g:uncultured |
| OTU_894 | 0 | 2.75 | 0.25 | k:Bacteria,p:Bacteroidota,c:Bacteroidia,o:Bacteroidales,f:Muribaculaceae |
| OTU_898 | 0 | 0 | 6.5 | k:Bacteria,p:Firmicutes,c:Clostridia,o:Clostridiales,f:Clostridiaceae,g:Clostridium,s:Clostridium facile |
| OTU_90 | 0 | 1.25 | 86.75 | k:Bacteria,p:Firmicutes,c:Clostridia,o:Lachnospirales,f:Lachnospiraceae,g:Lachnospiraceae NK4A136 group,s:uncultured |
| OTU_902 | 0 | 4.25 | 10.25 | k:Bacteria,p:Firmicutes,c:Clostridia,o:Lachnospirales,f:Lachnospiraceae,g:uncultured |
| OTU_903 | 0 | 2.5 | 2.5 | k:Bacteria,p:Firmicutes,c:Clostridia,o:Peptostreptococcales-Tissierellales,f:Anaerovoracaceae,g:XIII UCG-001,s:uncultured |
| OTU_905 | 0 | 0 | 1.75 | k:Bacteria,p:Bacteroidota,c:Bacteroidia,o:Bacteroidales,f:Muribaculaceae,g,uncultured |
| OTU_907 | 0 | 0 | 5 | k:Bacteria,p:Firmicutes,c:Clostridia,o:Clostridia UCG-014 |
| OTU_909 | 0 | 0 | 1.25 | k:Bacteria,p:Desulfobacterota,c:Desulfovibrionia,o:Desulfovibrionales,f:Desulfovibrionaceae,g:Bilophila,s:uncultured |
| OTU_91 | 0 | 0 | 88.25 | k:Bacteria,p:Bacteroidota,c:Bacteroidia,o:Bacteroidales,f:Muribaculaceae |
| OTU_912 | 0 | 1 | 0 | k:Bacteria,p:Actinobacteriota,c:Actinobacteria,o:Bifidobacteriales,f:Bifidobacteriaceae,g:Bifidobacterium |
| OTU_914 | 0 | 0 | 2 | k:Bacteria,p:Firmicutes,c:Clostridia,o:Oscillospirales,f:UCG-010 |
| OTU_9 | 7454.5 | 17.75 | 12 | k:Bacteria,p:Campilobacterota,c:Campylobacteria,o:Campylobacterales,f:Helicobacteraceae,g:Helicobacter,s:Helicobacter muridarum |
| OTU_916 | 0 | 0 | 1.25 | k:Bacteria,p:Firmicutes,c:Clostridia,o:Oscillospirales |
| OTU_917 | 0 | 0 | 1.5 | k:Bacteria,p:Firmicutes,c:Clostridia,o:Clostridiales,f:Christensenellaceae,g:Guopingia |
| OTU_918 | 0 | 3.25 | 2.75 | k:Bacteria,p:Firmicutes,c:Clostridia,o:Oscillospirales,f:Oscillospiraceae,g:Colidextribacter,s:uncultured |
| OTU_919 | 0 | 0 | 2 | k:Bacteria,p:Firmicutes,c:Clostridia,o:Clostridiales,f:Lachnospiraceae,g:PAC001296 |
| OTU_920 | 0 | 0 | 1.75 | k:Bacteria,p:Firmicutes,c:Clostridia,o:Borkfalkiales,f:Borkfalkiaceae,g:PAC001371 |
| OTU_92 | 0 | 46.5 | 0.5 | k:Bacteria,p:Bacteroidota,c:Bacteroidia,o:Bacteroidales,f:Bacteroidaceae,g:Bacteroides,s:Bacteroides finegoldii |
| OTU_922 | 0 | 6.75 | 0 | k:Bacteria,p:Proteobacteria,c:Gammaproteobacteria,o:Burkholderiales,f:Neisseriaceae,g:Neisseria,s:Neisseria weixii |
| OTU_924 | 0 | 27.25 | 107.5 | k:Bacteria,p:Firmicutes,c:Clostridia,o:Oscillospirales,f:Oscillospiraceae,g:Colidextribacter,s:uncultured |
| OTU_926 | 0 | 0 | 10.25 | k:Bacteria,p:Bacteroidota,c:Bacteroidia,o:Bacteroidales,f:Muribaculaceae |
| OTU_927 | 0 | 0.75 | 0 | k:Bacteria,p:Actinobacteriota,c:Coriobacteriia,o:Coriobacteriales,f:Eggerthellaceae |
| OTU_929 | 0 | 0 | 1.5 | k:Bacteria,p:Firmicutes,c:Clostridia,o:Lachnospirales,f:Lachnospiraceae,g:Blautia,s:uncultured |
| OTU_930 | 0 | 3.75 | 3 | k:Bacteria,p:Bacteroidota,c:Bacteroidia,o:Bacteroidales,f:Muribaculaceae |
| OTU_931 | 0.5 | 39.75 | 165.75 | k:Bacteria,p:Firmicutes,c:Clostridia,o:Lachnospirales,f:Lachnospiraceae,g:Lachnospiraceae NK4A136 group,s:uncultured |
| OTU_933 | 0 | 1 | 0.25 | k:Bacteria,p:Firmicutes,c:Clostridia,o:Clostridiales,f:Lachnospiraceae,g:PAC002765 |
| OTU_934 | 0 | 0 | 2.5 | k:Bacteria,p:Firmicutes,c:Clostridia,o:Clostridiales,f:Lachnospiraceae,g:PAC001165 |
| OTU_935 | 2 | 1.5 | 0 | k:Bacteria,p:Proteobacteria,c:Alphaproteobacteria,o:Sphingomonadales,f:Sphingomonadaceae,g:Sphingomonas |
| OTU_936 | 0 | 6.25 | 3.75 | k:Bacteria,p:Bacteroidota,c:Bacteroidia,o:Bacteroidales,f:Muribaculaceae |
| OTU_937 | 0 | 0 | 1 | k:Bacteria,p:Firmicutes,c:Clostridia,o:Clostridiales,f:Lachnospiraceae,g:Sporofaciens,s:uncultured |
| OTU_938 | 0 | 1 | 0.25 | k:Bacteria,p:Bacteroidota,c:Bacteroidia,o:Bacteroidales,f:Muribaculaceae |
| OTU_939 | 0 | 0 | 2.5 | k:Bacteria,p:Firmicutes,c:Clostridia,o:Clostridia UCG-014 |
| OTU_941 | 0 | 1.5 | 1.25 | k:Bacteria,p:Firmicutes,c:Clostridia,o:Monoglobales,f:Monoglobaceae,g:Monoglobus,s:uncultured |
| OTU_945 | 3 | 0 | 0 | k:Bacteria,p:Proteobacteria,c:Alphaproteobacteria,o:Rhizobiales,f:Beijerinckiaceae,g:Methylobacterium-Methylorubrum |
| OTU_946 | 0 | 0 | 0.75 | k:Bacteria,p:Firmicutes,c:Clostridia,o:Borkfalkiales,f:PAC001219,g:PAC001301 |
| OTU_947 | 0 | 1.75 | 1.5 | k:Bacteria,p:Firmicutes,c:Clostridia,o:Lachnospirales,f:Lachnospiraceae,g:Roseburia,s:uncultured |
| OTU_951 | 0.5 | 253 | 0 | k:Bacteria,p:Proteobacteria,c:Gammaproteobacteria,o:Enterobacterales,f:Enterobacteriaceae,g:Superficieibacter,s:Superficieibacter electus |
| OTU_952 | 0 | 2.5 | 0 | k:Bacteria,p:Acidobacteriota,c:Blastocatellia,o:Pyrinomonadales,f:Pyrinomonadaceae,g:RB41,s:uncultured |
| OTU_955 | 0 | 0.25 | 3.25 | k:Bacteria,p:Firmicutes,c:Clostridia,o:Lachnospirales,f:Lachnospiraceae,g:Lachnospiraceae FCS020 group |
| OTU_957 | 0 | 0 | 3 | k:Bacteria,p:Firmicutes,c:Clostridia,o:Borkfalkiales,f:Borkfalkiaceae,g:Borkfalkia,s:Borkfalkia ceftriaxoniphila |
| OTU_96 | 0 | 17.25 | 62.75 | k:Bacteria,p:Bacteroidota,c:Bacteroidia,o:Bacteroidales,f:Marinifilaceae,g:Odoribacter |
| OTU_962 | 0 | 0 | 0.75 | k:Bacteria,p:Acidobacteriota,c:Blastocatellia,o:Pyrinomonadales,f:Pyrinomonadaceae,g:RB41 |
| OTU_966 | 5 | 0 | 0 | k:Bacteria,p:Bacteroidota,c:Bacteroidia,o:Bacteroidales,f:Porphyromonadaceae,g:Porphyromonas |
| OTU_969 | 0 | 0 | 1.75 | k:Bacteria,p:Firmicutes,c:Clostridia,o:Clostridia UCG-014 |
| OTU_970 | 0 | 0 | 3 | k:Bacteria,p:Bacteroidota,c:Bacteroidia,o:Bacteroidales,f:Muribaculaceae |
| OTU_97 | 0 | 46.75 | 10 | k:Bacteria,p:Firmicutes,c:Clostridia,o:Oscillospirales,f:Ruminococcaceae,g:uncultured |
| OTU_971 | 0 | 0 | 0.75 | k:Bacteria,p:Firmicutes,c:Clostridia,o:Clostridiales,f:Lachnospiraceae,g:PAC001118 |
| OTU_974 | 0 | 0 | 0.75 | k:Bacteria,p:Firmicutes,c:Clostridia,o:Clostridiales,f:Lachnospiraceae,g:PAC001118 |
| OTU_975 | 0 | 7.5 | 2.5 | k:Bacteria,p:Bacteroidota,c:Bacteroidia,o:Bacteroidales,f:Muribaculaceae,g:RIAY |
| OTU_976 | 0 | 3.75 | 1 | k:Bacteria,p:Bacteroidota,c:Bacteroidia,o:Bacteroidales,f:Muribaculaceae,g:PAC001765 |
| OTU_978 | 0 | 0 | 2 | k:Bacteria,p:Bacteroidota,c:Bacteroidia,o:Bacteroidales,f:Prevotellaceae,g:Prevotellaceae UCG-003 |
| OTU_980 | 0 | 1.75 | 1 | k:Bacteria,p:Bacteroidota,c:Bacteroidia,o:Bacteroidales,f:Muribaculaceae,g:PAC001765 |
| OTU_98 | 1.5 | 0.25 | 251.75 | k:Bacteria,p:Firmicutes,c:Clostridia,o:Lachnospirales,f:Lachnospiraceae,g:NK4A136 group,s:uncultured |
| OTU_983 | 0 | 4 | 0 | k:Bacteria,p:Firmicutes,c:Bacilli,o:Acholeplasmatales,f:Acholeplasmataceae,g:Anaeroplasma,s:uncultured |
| OTU_985 | 0 | 0.25 | 1 | k:Bacteria,p:Firmicutes,c:Clostridia,o:Oscillospirales |
| OTU_987 | 0 | 0 | 4 | k:Bacteria,p:Firmicutes,c:Clostridia,o:Lachnospirales,f:Lachnospiraceae,g:Blautia,s:uncultured |
| OTU_988 | 0 | 1.75 | 14.25 | k:Bacteria,p:Firmicutes,c:Clostridia,o:Lachnospirales,f:Lachnospiraceae,g:GCA-900066575,s:uncultured |
| OTU_991 | 0 | 1.5 | 1.5 | k:Bacteria,p:Actinobacteriota,c:Coriobacteriia,o:Coriobacteriales,f:Eggerthellaceae |
| OTU_994 | 0 | 0.75 | 1.5 | k:Bacteria,p:Actinobacteriota,c:Coriobacteriia,o:Coriobacteriales,f:Eggerthellaceae,g:Enterorhabdus,s:uncultured |
| OTU_999 | 0 | 2.25 | 0 | k:Bacteria,p:Actinobacteriota,c:Actinobacteria,o:Micrococcales,f:Micrococcaceae,g:Glutamicibacter,s:Glutamicibacter creatinolyticus |

**Figure S3. Bacterial classification at Phylum, Order, Family, and Genus levels among the three groups**

| **Phylum** | **Lianhe** | **Nujiang** | **Ruili** |
| --- | --- | --- | --- |
| Proteobacteria | 0.753118 | 0.518013 | 0.690631 |
| Firmicutes | 0.205699 | 0.452857 | 0.211021 |
| Campilobacterota | 0.002964 | 0.003281 | 0.096358 |
| Bacteroidota | 0.034606 | 0.014041 | 0.000484 |
| Actinobacteriota | 0.002284 | 0.008709 | 0.000436 |
| Desulfobacterota | 0.000243 | 0.002037 | 0 |
| Fusobacteriota | 4.62E-05 | 0.000312 | 0.000962 |
| Spirochaetota | 0.000549 | 5.45E-05 | 0 |
| Deferribacterota | 0 | 0.000416 | 0 |
|  |  |  |  |
| **Order** |  |  |  |
| Burkholderiales | 0.715767 | 0.329776 | 0.662107 |
| Lactobacillales | 0.106380 | 0.415623 | 0.186689 |
| Enterobacterales | 0.002474 | 0.169119 | 0.004569 |
| Lachnospirales | 0.058064 | 0.014485 | 0.000239 |
| Campylobacterales | 0.002964 | 0.003281 | 0.096358 |
| Bacteroidales | 0.034577 | 0.013932 | 0.000356 |
| Pseudomonadales | 0.030404 | 0.008167 | 0.014612 |
| Oscillospirales | 0.023296 | 0.005329 | 6.38E-05 |
| Clostridia_vadinBB60_group | 0.011648 | 0.000369 | 5.32E-05 |
| Mycoplasmatales | 0.000107 | 3.22E-05 | 0.022820 |
| Pasteurellales | 0 | 0.009636 | 0.000138 |
| Bacillales | 9.24E-05 | 0.006863 | 1.06E-05 |
| Veillonellales-Selenomonadales | 0 | 0.006303 | 0 |
| Rhizobiales | 0.001442 | 0.000203 | 0.008282 |
| Micrococcales | 0.001252 | 0.002158 | 0.000202 |
| Bifidobacteriales | 1.03E-05 | 0.002614 | 0 |
| Peptostreptococcales-Tissierellales | 0.001537 | 0.000755 | 0.000723 |
| Actinomycetales | 0.000328 | 0.002116 | 9.58E-05 |
| Sphingomonadales | 0.001845 | 0.000349 | 0.000627 |
| Desulfovibrionales | 0.000243 | 0.002037 | 0 |
|  |  |  |  |
| **Family** |  |  |  |
| Alcaligenaceae | 0.534761 | 0.273018 | 0.538210 |
| Lactobacillaceae | 0.104722 | 0.239891 | 0.185402 |
| Burkholderiaceae | 0.180077 | 0.056093 | 0.123839 |
| Streptococcaceae | 0.000865 | 0.174815 | 0.000633 |
| Enterobacteriaceae | 0.002441 | 0.166581 | 0.004479 |
| Lachnospiraceae | 0.058064 | 0.014485 | 0.000239 |
| Helicobacteraceae | 0.002964 | 0.001467 | 0.096358 |
| Pseudomonadaceae | 0.029867 | 0.004892 | 0.014591 |
| (Unassigned) | 0.015416 | 0.004699 | 0.000356 |
| Muribaculaceae | 0.010408 | 0.005068 | 0 |
| Ruminococcaceae | 0.012885 | 0.001340 | 2.66E-05 |
| Oscillospiraceae | 0.009463 | 0.002488 | 3.72E-05 |
| Mycoplasmataceae | 0.000107 | 3.22E-05 | 0.022820 |
| Pasteurellaceae | 0 | 0.009636 | 0.000138 |
| F082 | 0.009381 | 7.44E-06 | 1.06E-05 |
| Bacteroidaceae | 0.002731 | 0.005262 | 0.000228 |
| Veillonellaceae | 0 | 0.006278 | 0 |
| Prevotellaceae | 0.005480 | 0.000282 | 0 |
| Rhizobiaceae | 0.001111 | 0.000131 | 0.008000 |
| Bacillaceae | 8.73E-05 | 0.004781 | 1.06E-05 |
|  |  |  |  |
| **Genus** |  |  |  |
| Achromobacter | 0.534756 | 0.272530 | 0.538210 |
| Lactobacillus | 0.104722 | 0.239891 | 0.185402 |
| Burkholderia-Caballeronia-Paraburkholderia | 0.113747 | 0.048905 | 0.105066 |
| Streptococcus | 0.000636 | 0.174629 | 0.000436 |
| Escherichia-Shigella | 0.002312 | 0.163165 | 0.000638 |
| Ralstonia | 0.060356 | 0.006717 | 0.016793 |
| (Unassigned) | 0.050728 | 0.019945 | 0.004117 |
| Helicobacter | 0.002964 | 0.001467 | 0.096358 |
| Lachnospiraceae_NK4A136_group | 0.038710 | 0.004709 | 0.000117 |
| Pseudomonas | 0.029867 | 0.004892 | 0.014591 |
| uncultured | 0.013701 | 0.004964 | 0.000313 |
| Ruminococcus | 0.010834 | 0.000156 | 2.66E-05 |
| Mycoplasma | 2.57E-05 | 3.22E-05 | 0.021740 |
| Bacteroides | 0.002731 | 0.005262 | 0.000228 |
| Cupriavidus | 0.005967 | 0.000468 | 0.001978 |
| Veillonella | 0 | 0.006137 | 0 |
| Bacillus | 8.73E-05 | 0.004781 | 1.06E-05 |
| Acinetobacter | 0.000513 | 0.003274 | 2.13E-05 |
| Odoribacter | 0.003193 | 0.000622 | 0 |
| Prevotella | 0.003665 | 0.000109 | 0 |

**Table S4. Bray Curtis distances between intestinal microbial community profiles**

| Samples | NO2 | RO2 | RO4 | LO2 | LO4 | PO2 | PO4 | H03 | JO2 | JO4 |
| --- | --- | --- | --- | --- | --- | --- | --- | --- | --- | --- |
| NO2 (Nujiang) | 0 | 0.59907 | 0.078834 | 0.062374 | 0.033754 | 0.956893 | 0.731991 | 0.996244 | 0.221493 | 0.986541 |
| RO2 (Ruili) | 0.59907 | 0 | 0.597375 | 0.583818 | 0.593747 | 0.95807 | 0.774125 | 0.934441 | 0.576633 | 0.872254 |
| RO4 (Ruili) | 0.078834 | 0.597375 | 0 | 0.073639 | 0.054388 | 0.967629 | 0.715415 | 0.977193 | 0.229695 | 0.975878 |
| LO2 (Lianhe) | 0.062374 | 0.583818 | 0.073639 | 0 | 0.035101 | 0.949084 | 0.734275 | 0.989544 | 0.190931 | 0.986091 |
| LO4 (Lianhe) | 0.033754 | 0.593747 | 0.054388 | 0.035101 | 0 | 0.953587 | 0.728999 | 0.996552 | 0.199541 | 0.986434 |
| PO2 (Nujiang) | 0.956893 | 0.95807 | 0.967629 | 0.949084 | 0.953587 | 0 | 0.96485 | 0.992328 | 0.949796 | 0.945766 |
| PO4 (Nujiang) | 0.731991 | 0.774125 | 0.715415 | 0.734275 | 0.728999 | 0.96485 | 0 | 0.877557 | 0.764138 | 0.742673 |
| H03 (Nujiang) | 0.996244 | 0.934441 | 0.977193 | 0.989544 | 0.996552 | 0.992328 | 0.877557 | 0 | 0.995935 | 0.90215 |
| JO2 (Lianhe) | 0.221493 | 0.576633 | 0.229695 | 0.190931 | 0.199541 | 0.949796 | 0.764138 | 0.995935 | 0 | 0.986326 |
| JO4 (Lianhe) | 0.986541 | 0.872254 | 0.975878 | 0.986091 | 0.986434 | 0.945766 | 0.742673 | 0.90215 | 0.986326 | 0 |

**Table S5. Euclidean distances between intestinal microbial community profiles**

| Samples | NO2 | RO2 | RO4 | LO2 | LO4 | PO2 | PO4 | H03 | JO2 | JO4 |
| --- | --- | --- | --- | --- | --- | --- | --- | --- | --- | --- |
| NO2 (Nujiang) | 0 | 0.9389901 | 0.0693858 | 0.0596411 | 0.0324689 | 1.3994794 | 1.1909314 | 1.4135106 | 0.3353568 | 1.4000468 |
| RO2 (Ruili) | 0.9389901 | 0 | 0.9355431 | 0.9376693 | 0.9385291 | 1.4035255 | 1.2855865 | 1.404846 | 0.944558 | 1.2250833 |
| RO4 (Ruili) | 0.0693858 | 0.9355431 | 0 | 0.0693568 | 0.0594607 | 1.3993903 | 1.1900277 | 1.4117246 | 0.3448845 | 1.3889521 |
| LO2 (Lianhe) | 0.0596411 | 0.9376693 | 0.0693568 | 0 | 0.0368645 | 1.398399 | 1.1912336 | 1.4134666 | 0.3167941 | 1.3998768 |
| LO4 (Lianhe) | 0.0324689 | 0.9385291 | 0.0594607 | 0.0368645 | 0 | 1.3992259 | 1.191 | 1.4135354 | 0.3293223 | 1.400135 |
| PO2 (Nujiang) | 1.3994794 | 1.4035255 | 1.3993903 | 1.398399 | 1.3992259 | 0 | 1.4054107 | 1.4133856 | 1.388635 | 1.4100474 |
| PO4 (Nujiang) | 1.1909314 | 1.2855865 | 1.1900277 | 1.1912336 | 1.191 | 1.4054107 | 0 | 1.3208469 | 1.2037799 | 0.8831163 |
| H03 (Nujiang) | 1.4135106 | 1.404846 | 1.4117246 | 1.4134666 | 1.4135354 | 1.4133856 | 1.3208469 | 0 | 1.4135689 | 1.3031968 |
| JO2 (Lianhe) | 0.3353568 | 0.944558 | 0.3448845 | 0.3167941 | 0.3293223 | 1.388635 | 1.2037799 | 1.4135689 | 0 | 1.3995923 |
| JO4 (Lianhe) | 1.4000468 | 1.2250833 | 1.3889521 | 1.3998768 | 1.400135 | 1.4100474 | 0.8831163 | 1.3031968 | 1.3995923 | 0 |

**Table S6. The mean decrease in accuracy and mean decrease in the Gini coefficient in the important OTU distribution using Random forest test**

| **Taxon** | **MeanDecreaseAccuracy** |  | **Taxon** | **MeanDecreaseGini** |
| --- | --- | --- | --- | --- |
| OTU_330 | 2.239492 |  | OTU_22 | 0.055623 |
| OTU_46 | 1.960102 |  | OTU_17 | 0.048526 |
| OTU_17 | 1.822437 |  | OTU_29 | 0.043985 |
| OTU_2 | 1.669110 |  | OTU_46 | 0.042666 |
| OTU_146 | 1.669110 |  | OTU_52 | 0.040185 |
| OTU_931 | 1.417050 |  | OTU_239 | 0.039266 |
| OTU_222 | 1.417050 |  | OTU_6 | 0.038222 |
| OTU_1907 | 1.417050 |  | OTU_16 | 0.035928 |
| OTU_1685 | 1.402752 |  | OTU_519 | 0.034836 |
| OTU_951 | 1.402752 |  | OTU_19 | 0.034036 |
| OTU_52 | 1.389425 |  | OTU_10 | 0.033774 |
| OTU_50 | 1.344062 |  | OTU_146 | 0.033171 |
| OTU_291 | 1.2070415 |  | OTU_51 | 0.033066 |
| OTU_9 | 1.0010015 |  | OTU_493 | 0.032066 |
| OTU_18 | 1.0010015 |  | OTU_489 | 0.031666 |
