# Supplementary figures and images for: Comparative analysis of the intestinal microbiome in Rattus norvegicus from different geographies
Source: Front Microbiol. 2023 Nov 3;14:1283453. doi: 10.3389/fmicb.2023.1283453 (PMC10655115; doi:10.3389/fmicb.2023.1283453)

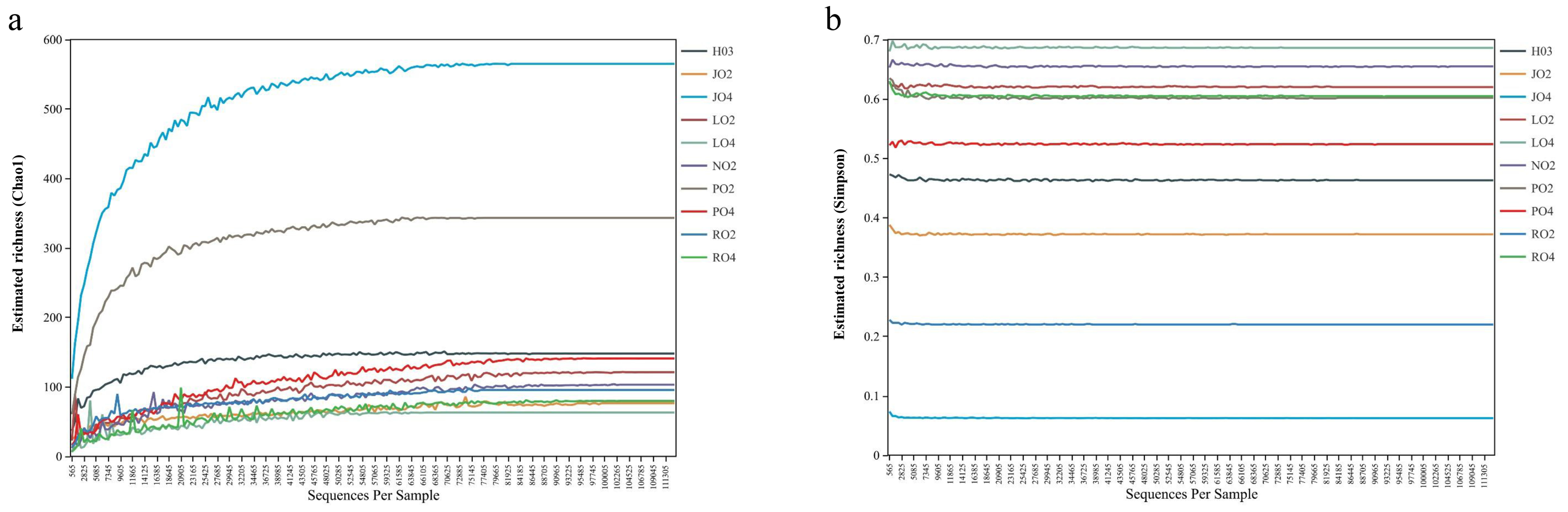

Supplement: Supplementary file 2 [file Image_1.JPEG]

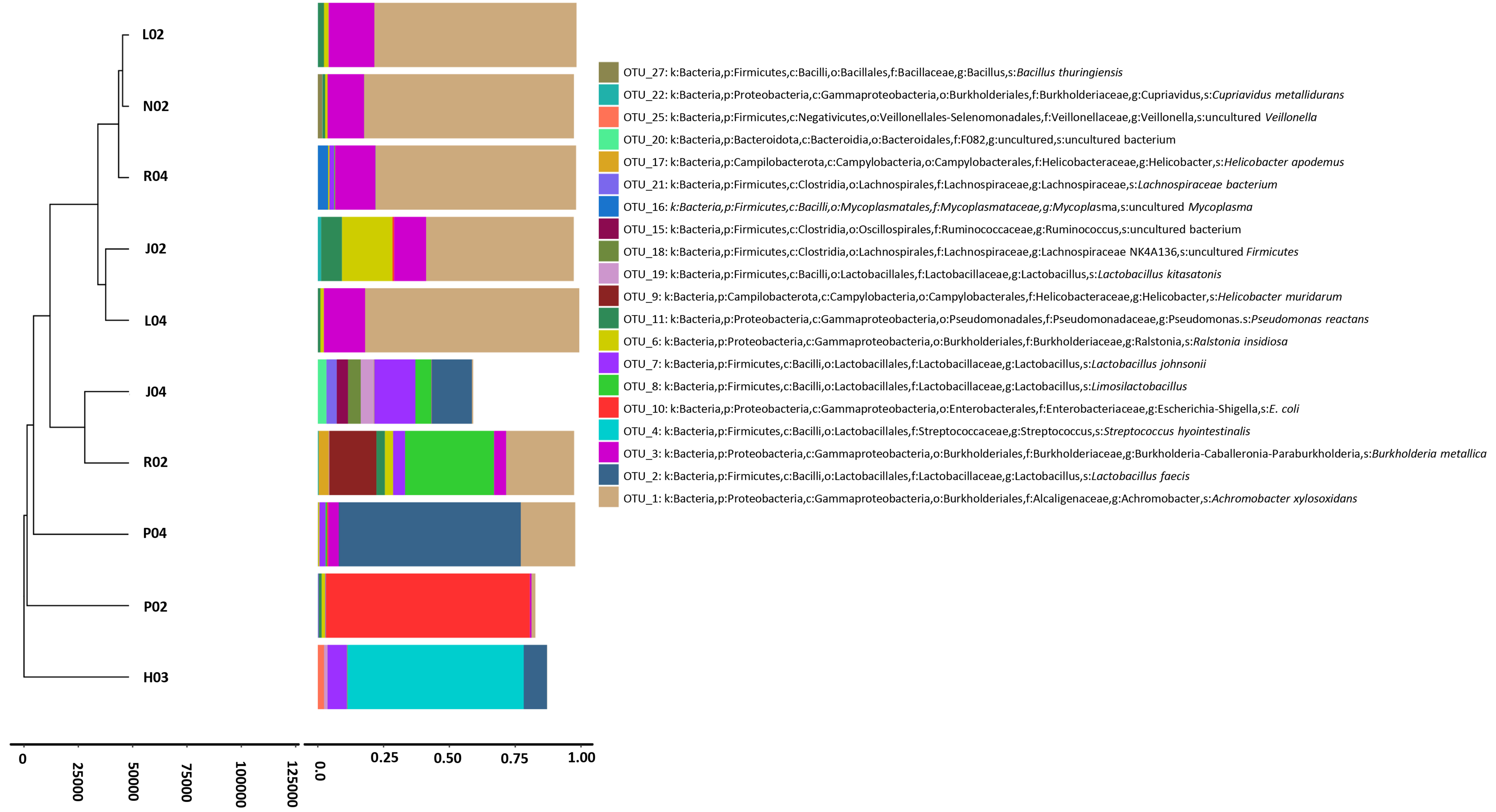

Supplement: Supplementary file 3 [file Image_2.JPEG]

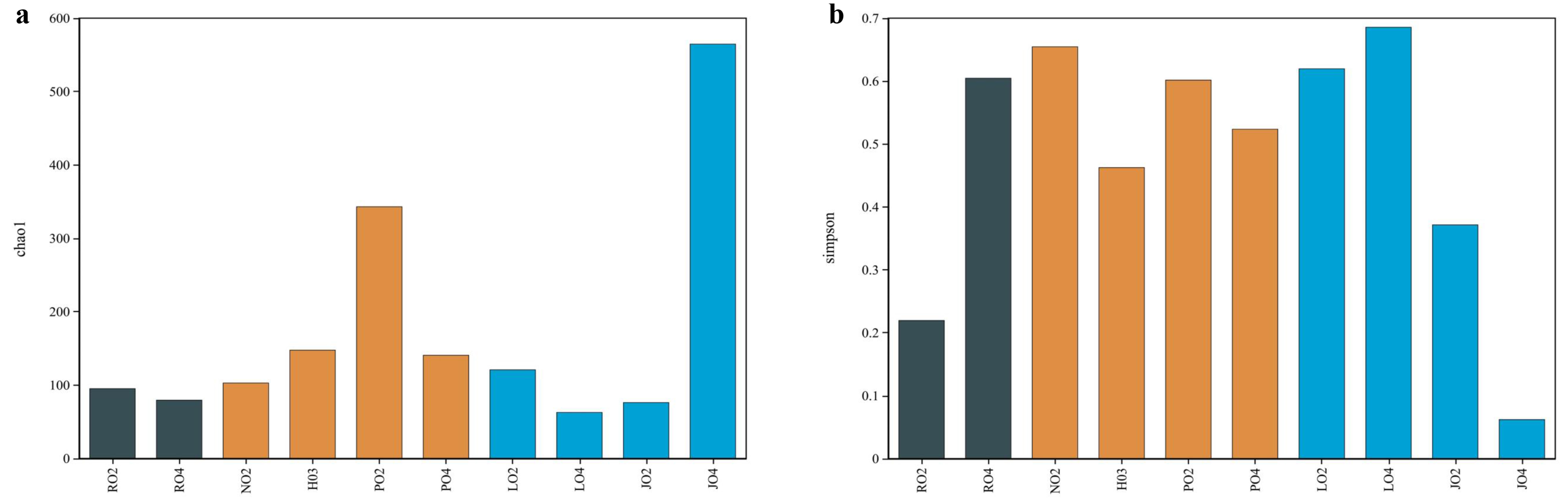

Supplement: Supplementary file 4 [file Image_3.JPEG]
